# Market Integration Pathways for Enhanced Geothermal Systems in Europe

**DOI:** 10.1038/s41467-026-77893-3

**Published:** 2026-10-16

**Authors:** Lukas Franken, Elisabeth Zeyen, Orestis Angelidis, Tom Brown, Daniel Friedrich

**Affiliations:** 1 School of Engineering, Institute for Energy Systems, University of Edinburgh, Edinburgh, UK; 2 Department of Digital Transformation in Energy Systems, Technische Universität Berlin, Berlin, Germany; 3 James Watt School of Engineering, University of Glasgow, Glasgow, UK

**Keywords:** Geothermal energy, Energy grids and networks, Energy economics

## Abstract

Enhanced geothermal systems can provide constant, reliable electricity and heat with minimal emissions, but high drilling costs and uncertain cost reductions leave their future unclear. We explore scenarios for the future adoption of these systems in a carbon-neutral, multi-sector European energy system. Here we show that in a net-zero system, heat (co-)generating enhanced geothermal plants at current cost can support 20–30 gigawatts (GW) of thermal capacity in Europe, primarily driven by district heating demands. If drilling costs were to decrease by approximately 60% relative to 2020 costs, the technology would become competitive in electricity markets, greatly expanding its market opportunity. However, the spatially dispersed rollout of district heating stands against the confined overlap of high geological potential with low potential for other renewables, which conditions the competitiveness of electricity-generating plants. This results in a challenge where the majority of the market potential depends on pan-European technology learning for cost reductions, emphasising that coordination is crucial in stakeholders’ efforts.

## Abstract (web summary)

The authors map how enhanced geothermal systems could enter a carbon-neutral European energy market: district heating sustains deployment at today’s drilling costs, while cost reductions of around 60% would unlock far larger electricity markets.

## Introduction

Geothermal energy has the potential to serve as a firm and renewable energy source by harnessing the abundant heat from the subsurface^1^. However, there is high uncertainty about future drilling costs, especially for Enhanced Geothermal Systems (EGS), resulting in varying projections of the geothermal contribution (1-4%) to the future energy mix^2–4^.

One of the primary differences between these estimates is the uncertain role of EGS. In EGS, “Enhanced” refers to the synthetic increase in deep, hot rock’s permeability to facilitate the heating of a working fluid for heat and/or electricity generation^5^. Unlike traditional geothermal systems, EGS is not restricted to regions with exceptional geological suitability but has broader applicability. However, high drilling costs currently make EGS economically uncompetitive, despite its significant untapped potential^2^. Should drilling costs decrease, EGS could play a crucial role in future energy supply.

Existing EGS projects often improve their economic viability by supplying heat in addition to electricity^5^. In these cases, thermal energy can be distributed to end users with high efficiencies compared to the much lower efficiencies achievable in electricity generation (approximately 10-20%, depending on rock formation temperature)^6,7^. This approach has enabled the development of over ten smaller EGS projects in Europe, producing between 1 MW_th_ (thermal) and 11 MW_th_, all of which generate or co-generate heat^8–10^. Heat generation is particularly beneficial as it improves EGS’ economic prospects^7^, contributes low-carbon energy in the heating sector^11^, better accommodates suboptimal geothermal fluid temperatures^12^, and is often supported by additional policy incentives^13^.

Recent technical advances highlight the ongoing cost reductions of EGS technology. A shortcoming of conventional reservoir-stimulation methods in EGS is the high probability that the induced fracture network will lack sufficient permeability for economically viable circulation. Field campaigns conducted between 2020 and 2025 at the Blue Mountain and UtahFORGE sites have validated a multistage “plug-and-perf” strategy: Instead of propagating a single massive fracture, each horizontal well is treated in roughly a dozen discrete stages, generating a laterally extensive fracture network. This approach has delivered greater injectivity and improved thermal sweep efficiency^14–16^ and is projected to reduce the Levelized Cost of Electricity (LCOE) by about 25%^17^.

Existing modelling efforts evaluate the potential of EGS either by using geophysical data or modelling its market competitiveness in the whole energy system. The estimates of EGS potential and LCOE vary significantly between studies. A recent study estimates the global EGS potential to be around 1.5 TW_el_ (electric) at or below an LCOE of approximately 150€${{{{\rm{MWh}}}}}_{{{{\rm{el}}}}}^{-1}$ (euros per megawatt-hour of electricity). Current technological capabilities allow for the extraction of 227 GW_el_ of this potential in Europe^2^. In contrast, an earlier study estimated that the opportunity for EGS in Europe by 2050 could reach 522 GW_el_ at an LCOE below 100€${{{{\rm{MWh}}}}}_{{{{\rm{el}}}}}^{-1}$^18^. Longa et al.^4^ inserted this potential into an integrated assessment model and showed a cost-optimal EGS capacity of around 25 GW_el_ when competing with other technologies. The exploration of EGS potential in an investment and dispatch power model for the United States West Coast highlights the importance of modelling EGS features that are only apparent at high temporal resolution, such as flexible operation^19^. More recent research suggests that the economic prospects of EGS could be further enhanced by extracting lithium alongside with heat and/or power generation^20^. However, broader and often influential energy outlooks that consider the entire energy mix–rather than geothermal specifically–arrive at more pessimistic projections for its deployment, notably the International Energy Agency (IEA)’s World Energy Outlook 2023^1^, the 2050 Energy Outlook of the International Renewable Energy Agency (IRENA)^21^, and the Intergovernmental Panel on Climate Change’s Sixth Assessment Report (2022)^22^.

Existing literature leaves open some of the questions most relevant to industry stakeholders and policymakers looking to invest in or promote EGS deployment. First, previous studies estimate the volume of EGS potential in Europe but offer limited insight into how that pathway could materialise. Without an assessment of the spatial distribution of market opportunities across varying levels of technological progress, stakeholders are left without the insight needed to support that development effectively. This shortcoming of past work arises from the computational burden associated with multi-year simulation horizons, which are commonly employed in integrated assessment models, such as those used, for instance, in Longa et al.^4^. As a result, the intricate interaction between the spatial distribution of energy demand, renewable generation opportunities, and energy transport–constrained by the transmission grid–is overlooked. Thus, while an estimate of overall EGS potential has been established, the findings of Dalla Longa et al.^4^ need to be expanded to provide a detailed projection of how a European energy future with EGS might manifest.

Second, it also remains unclear which level of cost reduction would permit EGS to enter the European energy market. The binary framing of cost development–either optimistic or conservative–applied in Dalla Longa et al.^4^ and Ricks et al.^19^ leaves limited room for this aspect of the analysis. A detailed account of the market opportunity associated with different levels of EGS costs, both in terms of volume and spatial distribution, would help indicate the ultimate value of potential technical advances. Meanwhile, studies that do not focus on geothermal supply–such as the IEA World Energy Outlook 2023^1^–favour a pessimistic EGS cost outlook, although, as a technology with limited current roll-out, it still offers considerable scope for learning-driven cost reductions. These two modelling approaches leave policymakers and investors uncertain about the likelihood that investing in EGS will pay off.

**Fig. 1:**
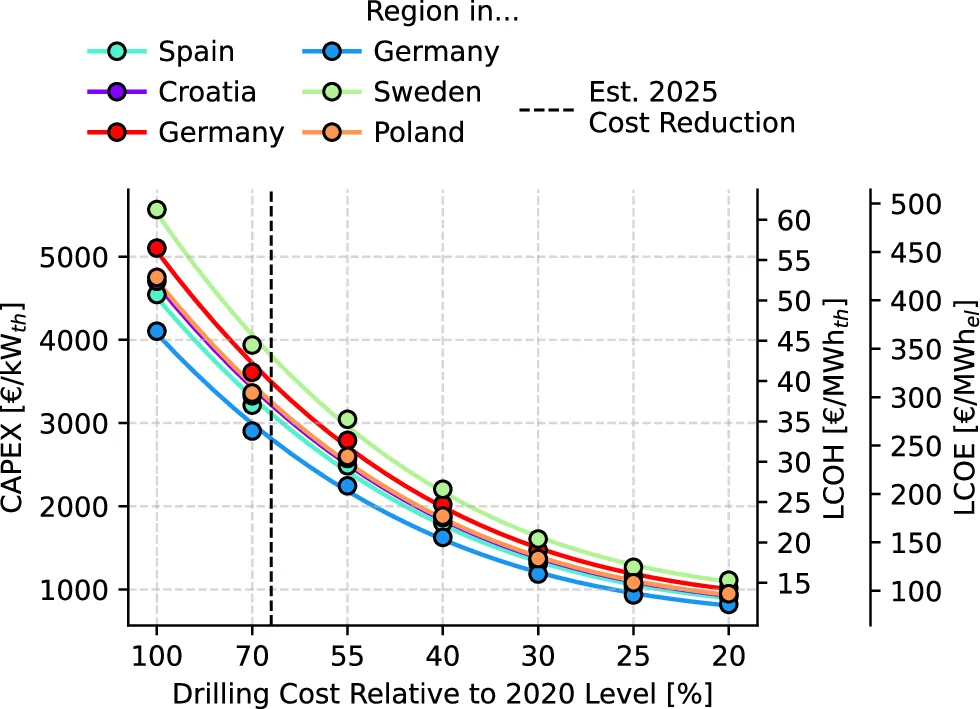
Drilling cost reduction scenarios evaluated in this study. The scenarios of potential drilling cost reductions are shown here for six out of 72 regions. All costs are assumed to decrease, however preserve the relative magnitude between regions, reflecting geological conditions. The scenarios cover the span of the cost reductions evaluated in Aghahosseini and Breyer^2^. The estimate of cost reductions achieved in 2025 (around 25%) is based on Aljubran and Horne^17^. Note the capital expenditure (CAPEX) values on the left refer to heat, not power capacity, i.e. before application of the efficiency factor. The levelized cost of heat (LCOH) and levelized cost of electricity (LCOE) values are computed directly from the CAPEX assuming a capacity factor of 95%, for a detailed overview we refer to the Methods.

**Fig. 2:**
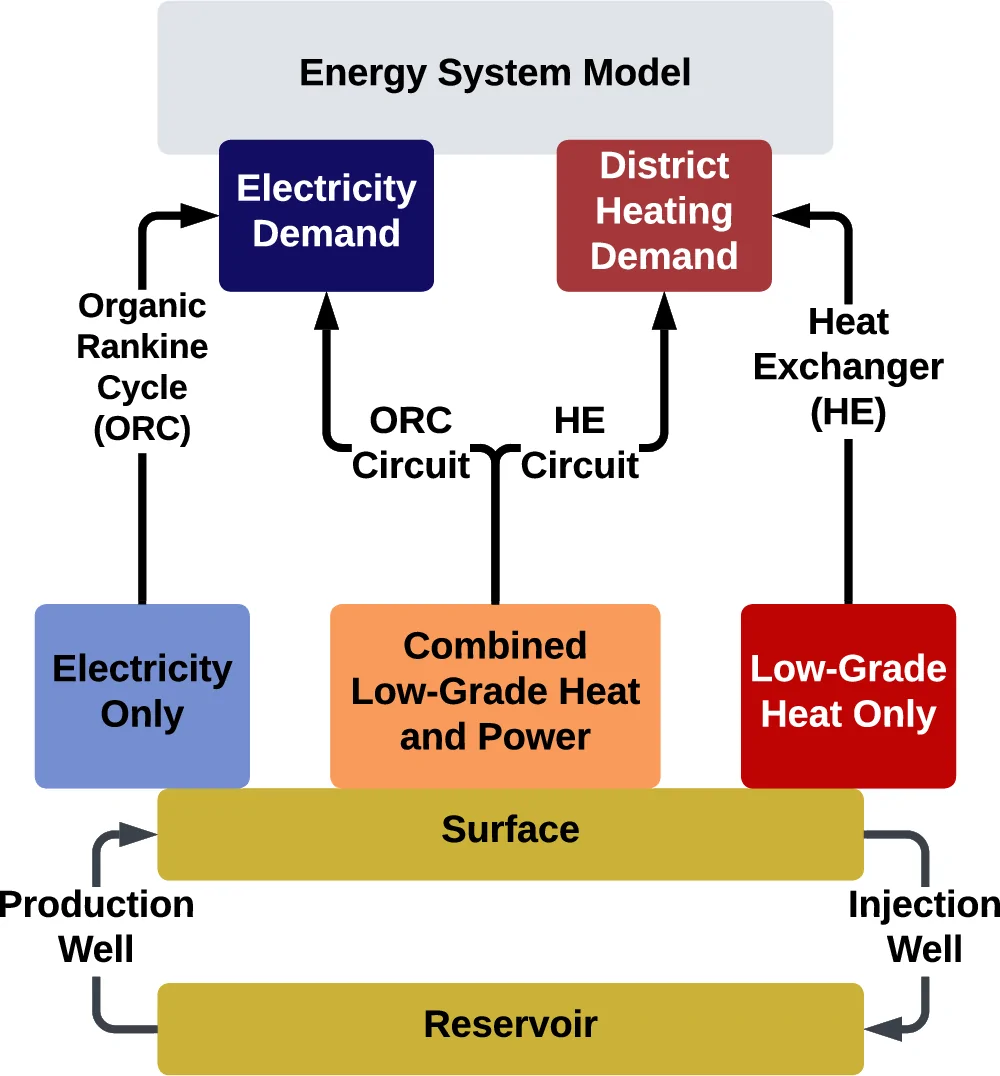
Model representation of Enhanced Geothermal Systems (EGS). The flowchart shows a simplified representation of the EGS model. Note that all model runs include one of the three modes where EGS generates only electricity, combined low-grade heat and power or low-grade heat only. While different in the technologies they represent on a surface level, the subsurface modelling remains the same between them. ORC: Organic Rankine Cycle.

Here, we address these gaps by analysing EGS’ future market opportunities in Europe with a high spatiotemporal resolution. We incorporate EGS at varying levels of drilling cost reduction (Fig. 1) into a sector-coupled, carbon-neutral European energy system model based on the open-source framework PyPSA-Eur, in three distinct modes of generation: (i) electricity only, (ii) combined low-grade heat and power, or (iii) low-grade heat only (Fig. 2, see Methods). In the latter two modes, the model supplies low-grade heat to district- and low-temperature industrial heat demands, and drilling costs follow existing estimates of geological suitability^2^. For each level of cost reduction, we characterise the EGS market opportunity in terms of volume, spatial distribution and the sectors supplied, and identify two phases of rollout: a first phase in which heat (co-)generation is crucial for EGS to be competitive, and a second phase in which a tipping point in drilling costs enables EGS to compete directly with wind and solar, unlocking a much larger market. We further estimate the learning rates that would make this rollout largely self-sustaining, with each step of cost reduction driving the subsequent one, and explore the cost-optimal spatial distribution of deployment, identifying barriers and opportunities that can accelerate or hinder its rollout. These findings provide context for both short-term EGS development and long-term investment and subsidy strategies that aim to support EGS deployment.

## Results

### Heat Generating EGS as Market Entry

**Fig. 3:**
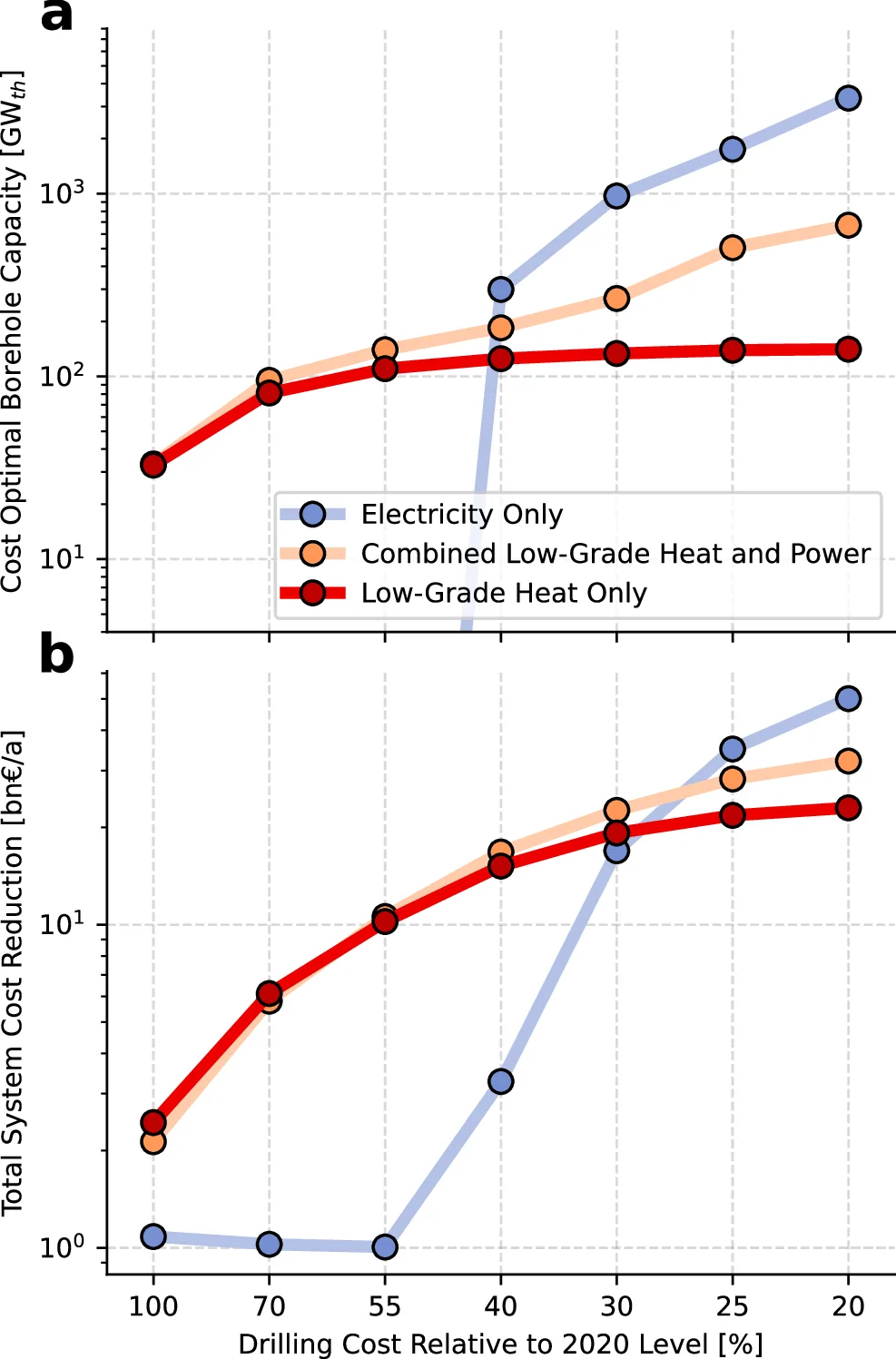
European market potential of Enhanced Geothermal Systems (EGS) by mode of operation. **a** Demand curves for borehole thermal capacity of EGS in Europe and **b** resulting total system cost reduction. The *y*-axis in **a** refers to heat capacity.

The cost competitiveness of EGS depends strongly on which energy service is provided (heat, electricity, or both) as well as the anticipated reduction in drilling cost (Fig. 3). Heat-generating EGS, at 2020 costs, is already cost-competitive in a carbon-neutral energy system, leading to a reduction in overall system costs by less than 1% ( <  5 billion € per year). Co-generation of power and heat offers greater savings, with total system cost reductions ranging from  <  1% to around 4% when compared to heat generation alone, which achieves savings of  < 1% to around 3%. For EGS to become competitive as a source of electricity generation alone, drilling costs would need to decrease to around 40% of their 2020 levels. This scenario yields the largest total system cost reduction, with costs decreasing by about 50 billion € per year ( ≈ 6%) if EGS costs were to reduce to 20% of their 2020 levels. However, the cost reduction per installed EGS unit is lowest for electricity generation, indicating that much of the installed electricity-generating EGS capacity is only marginally more cost-effective than alternative supply options. In terms of borehole heat capacity, electricity generation peaks at approximately 4000 GW_th_ (gigawatts thermal), whereas co-generation peaks at 700 GW_th_, and heat generation reaches a peak of 150 GW_th_. As EGS penetration increases, marginal energy prices at both electricity and district-heating nodes decrease. For power, the system-wide load-weighted average electricity price is around 88 €MWh^−1^ without EGS and, consistent with the reduction in system costs, falls to 70 €MWh^−1^ under the strongest EGS cost reductions. District-heating prices start at around 37 €MWh^−1^ and decline to roughly 20 €MWh^−1^ for medium cost reductions and under extreme EGS cost reductions to about 10 €MWh^−1^ (Supplementary Fig. 19). We note these are model-implied prices in a carbon-neutral system. They fall within the range reported in related literature (see for instance 2040 electricity price in Mann et al.^23^), but are subject to larger inter-study variation.

Scenarios with higher drilling cost reductions, find larger EGS market opportunities. By connecting each level of cost reduction with a market opportunity, we can estimate the technology learning rate needed to reach the subsequent next level of cost reduction given the available market size (see Methods). We find that the learning rate to be around 20% (Supplementary Fig. 2). For low cost reductions, where learning opportunity is limited due to smaller heating demand and inaccessible electricity markets, a slightly higher learning rate of approximately 25% is needed. As soon as electricity generation becomes the main application for EGS, the learning rate stabilises at around 20%. For context, historical learning-rate estimates are commonly reported at roughly 20 − 30% for solar photovoltaics (PV), 10 − 20% for onshore wind, and 10 − 15% for shale gas drilling (per doubling of cumulative deployment)^24,25^. Therefore, the learning rates estimated here, 20 − 25%, appear high but plausible. We further note that this is a local estimate; if technology learning occurs at a pan-European scale, the process could draw on the much larger market opportunities outside Europe, thereby substantially lowering the required learning rates (see Discussion).

**Fig. 4:**
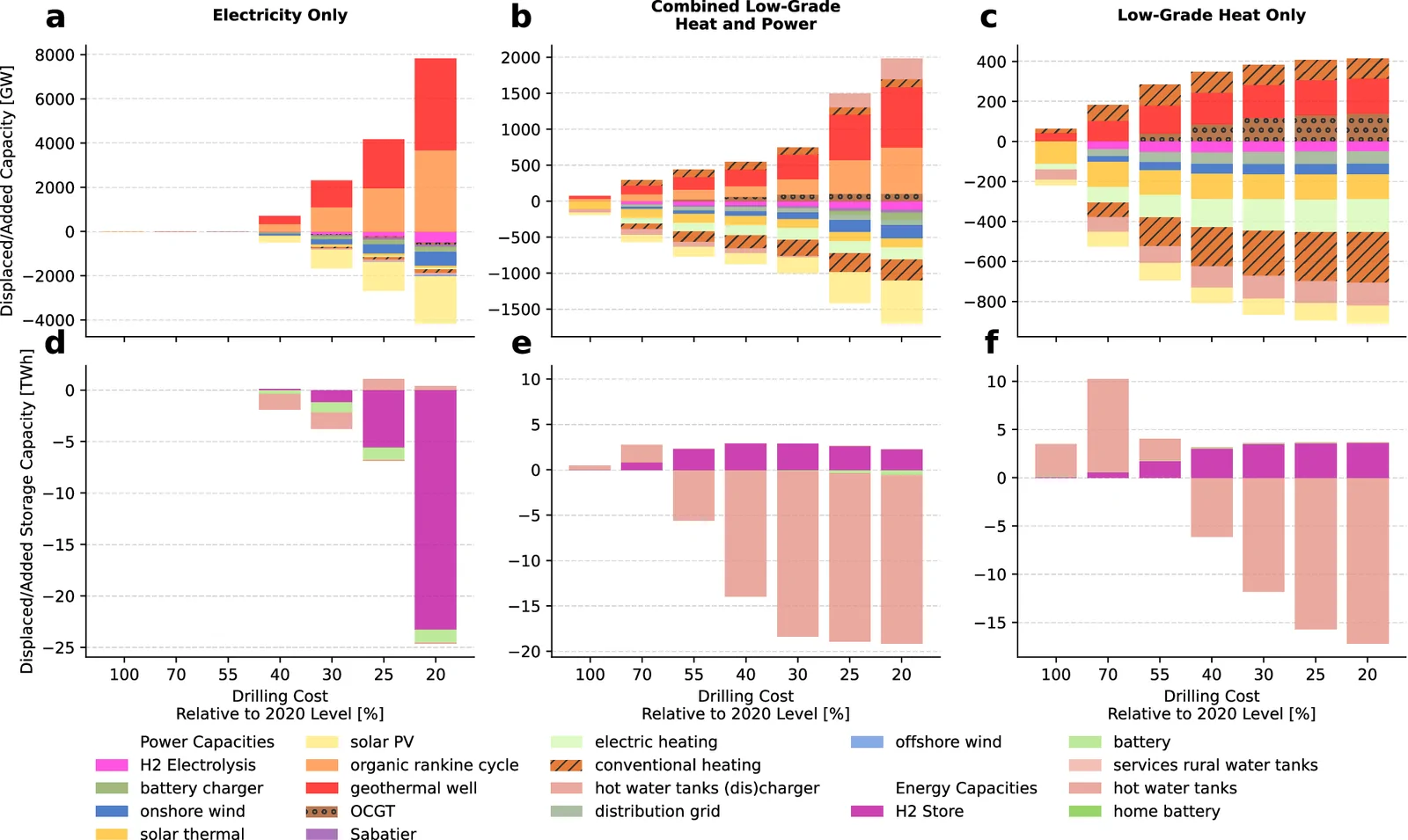
Technology capacities displaced by Enhanced Geothermal Systems (EGS) at increasing cost reductions. Changes of installed capacities are shown for different EGS cost reduction levels. Panels **a**–**c** show power capacities and **d**–**f** energy storage capacities. Positive values are added, negative ones are subtracted relative to a counterfactual where EGS is not available. PV: photovoltaics; OCGT: open-cycle gas turbine; H_2_: hydrogen.

As EGS capacity gradually increases in higher cost reduction scenarios, it displaces growing volumes of alternative generation capacities, primarily wind, solar, and fossil-fuel heat generators (Fig. 4). In district heating, EGS at low to medium cost reduction changes the composition of supply drastically, replacing most solar thermal, gas, and biomass heating (Supplementary Figs. 4, 5). The freed-up biomass is reallocated to heating in industry, and the emission budget is used for open-cycle gas turbine (Open-Cycle Gas Turbine (OCGT)) electricity generation instead. For electricity-generating EGS, displacement is more gradual, with 5 − 11% of solar and wind generation displaced at medium cost reductions and 16 − 40% displaced at extreme cost reductions (Supplementary Fig. 3). For power capacities, the displacement varies in volume, but its proportionality remains largely consistent as EGS costs decrease. In terms of energy capacity, however, displacement depends on the level of cost reduction; for higher-cost EGS, storage is added to accommodate baseload generation, while for lower-cost EGS, it is removed in favour of operating EGS capacity at lower capacity factors.

For electricity generation at high cost reductions, the model uses the dispatchable nature of EGS by removing around 22 TWh (terawatt-hours) hydrogen storage capacity (equals 40% of hydrogen storage capacity in EGS-free model), otherwise needed for balancing. For heat (co-)generating EGS at low cost reduction, hot water storages are initially added to the model to provide flexibility for the more base-load operation of EGS. In scenarios with more extensive cost reductions progress, these hot water storages are subsequently withdrawn along with solar thermal, leading to a decrease in installed thermal storage capacity of 20 TWh (63%) for EGS generating combined low-grade heat and power and 16 TWh (58%) for only low-grade heat. The thermal storage that the model displaces along with solar thermal exhibits a seasonal charging pattern rather than an intra-day one. Therefore, it is primarily seasonal balancing that becomes less valuable as dispatchable electrified heating, enabled by EGS, expands.

Interestingly, a similar but opposite dynamic is observed for hydrogen storage in heat (co-)generating applications, where the model consistently installs additional H_2_ storage capacity along with EGS. Its increase appears to be favoured over the hydrogen supply chain from variable renewables with electrolysis in the EGS-free model. The introduction of EGS also leads to substantial removal of fossil-fuel heating. This reduction of emissions within the heating sector allows the model to favour more fossil fuel generation in the power sector, building up to nearly 140 GW_th_ (approximately 50%) of additional OCGT capacity, depending on the EGS cost reduction factor.

### Spatial Aspects of EGS Adoption

The spatial distribution of EGS adoption results from the interplay of geological suitability, electricity prices in the incumbent system, and the distribution of energy demands. This interplay turns out to vary between electricity and heat (co-)generating EGS. For the former, both geological suitability and low renewable capacity factors are crucial for EGS to be competitive. These conditions are met only in a small subset of regions, primarily around Central Europe, the Benelux countries, and the Northern Balkans. In contrast, heat (co-)generating EGS is economically viable across a broader range of regions, even for low cost reductions. These regions include Central, Eastern, and Southeastern Europe, as well as Scandinavia. This distribution is mainly driven by the magnitude of district heating demands and only to a lesser extent by geological suitability.

**Fig. 5:**
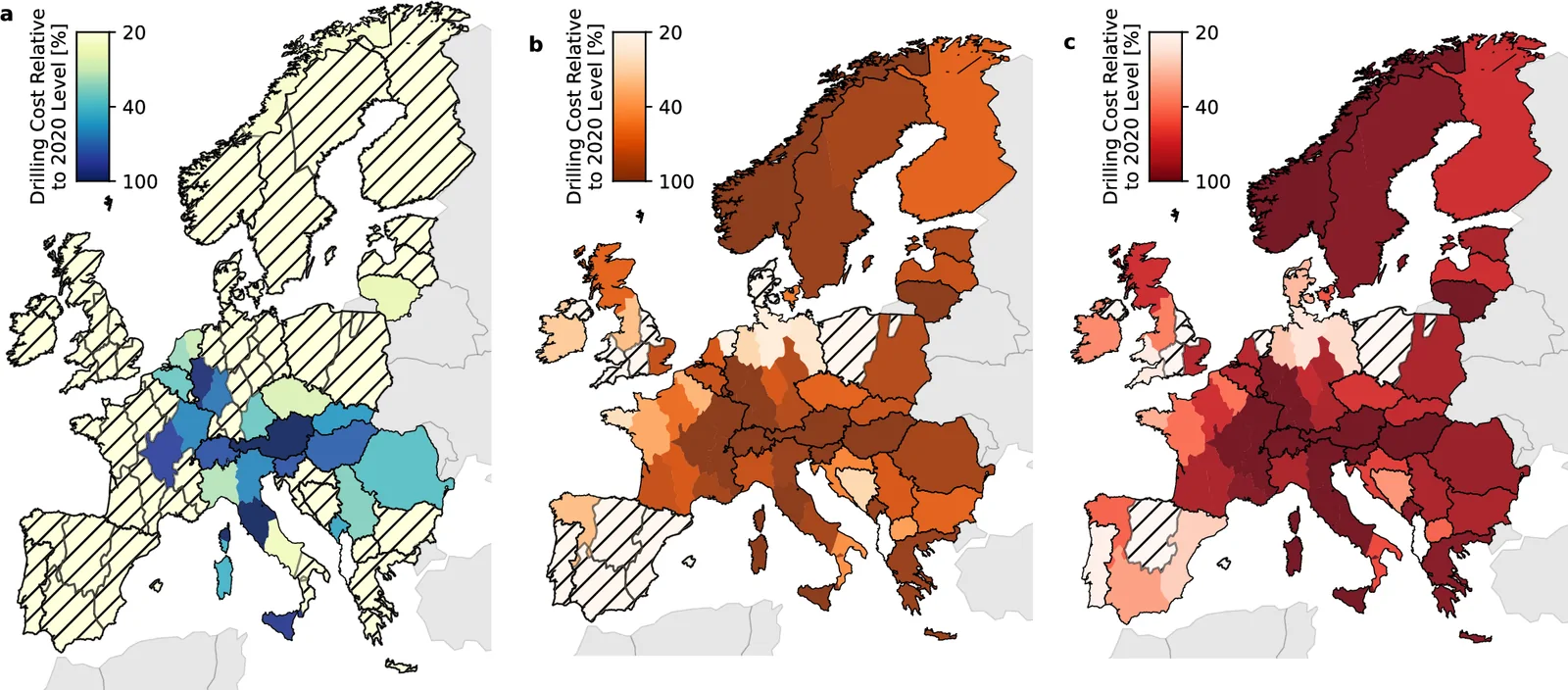
Regional drilling cost reductions required for Enhanced Geothermal Systems (EGS) competitiveness. Shown is the cost reduction relative to 2020's estimated cost required for EGS to contribute 10% of the respective demand per region. The maps show EGS generating **a** electricity, **b** combined low-grade heat and power and **c** low-grade heat only.

**Fig. 6:**
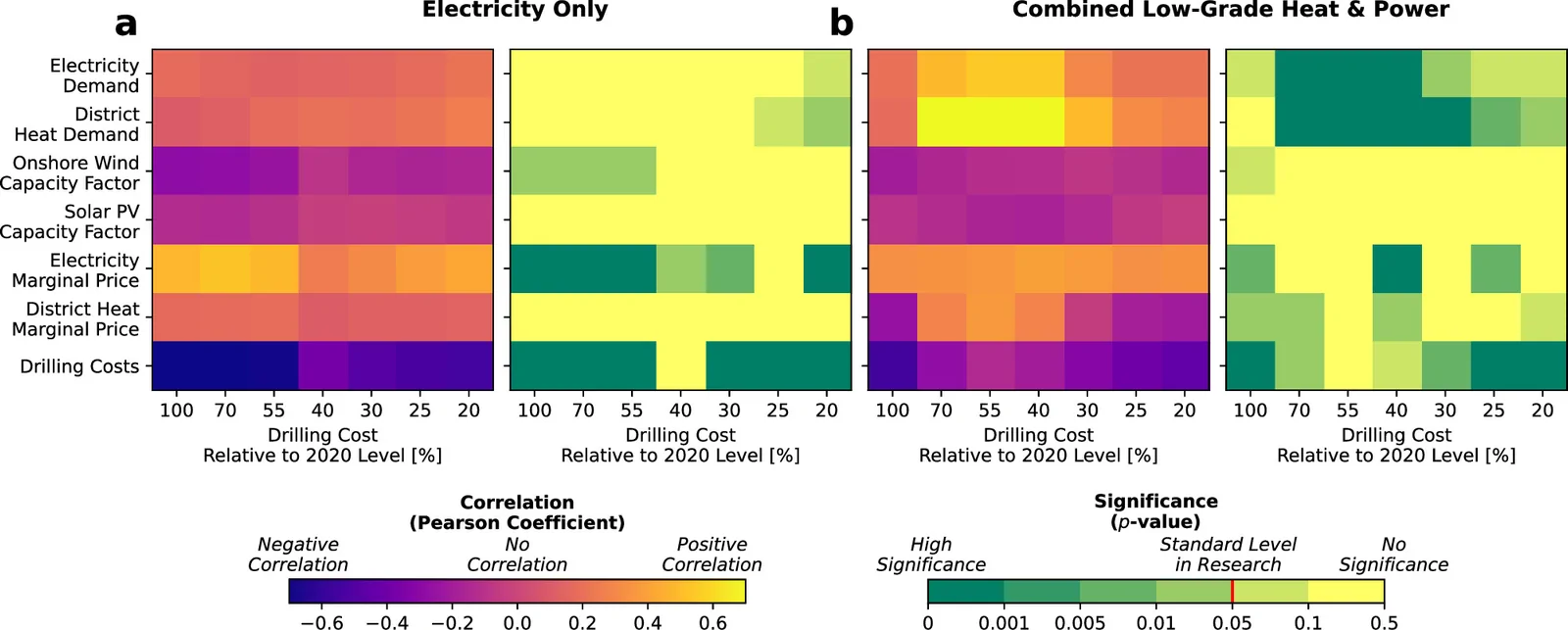
Regional features driving cost-optimal Enhanced Geothermal Systems (EGS) deployment. Correlation between cost-optimal borehole capacity and various features for **a** electricity-generating EGS and **b** combined low-grade heat and power EGS. In each panel, the left heatmap shows the correlations and the right heatmap their statistical significance. For the rows representing electricity and district heat marginal price, the values refer to marginal prices in a system without EGS. The respective figure for heat-generating EGS is provided in Supplementary Fig. 12. The statistical tests employed here are two-sided, the sample size is the numbers of regions in the model 72 which yields 70 degrees of freedom. The upper and lower 95% confidence intervals of Pearson Coefficients are reported in Supplementary Tables 2, 3, 4, 5. PV: photovoltaics.

Echoing the findings of the previous section, electricity-generating EGS is only cost-competitive in scenarios with significant cost reductions (Fig. 5). However, the spatial concentration of electricity-generating EGS in a small subset of Central European regions is surprising, given the much larger cost-optimal EGS borehole capacities observed in the first part of the results. Even at an extremely low cost of 20% relative to 2020’s levels, most regions do not see substantial capacity expansion. Looking at the correlation between region’s properties and their EGS uptake, we find that geological suitability is the main driver behind the concentrated adoption of electricity-producing EGS (Fig. 6). This is indicated by strong negative correlations between -0.6 and -0.3. However, a secondary predictor is the marginal price of electricity, with a correlation of around 0.4. High marginal prices are partially caused by low renewable capacity factors, a relationship also reflected in the minor negative correlations between EGS adoption and onshore wind capacity factors (of around -0.2).

EGS with heat generation is installed in more regions, even at only minor cost reductions of less than 50% of 2020’s level. These regions include Central Europe, mainly around the Alps, parts of the Balkans and the Baltics, as well as Norway and Sweden. If costs of around 40% of 2020’s levels are undercut, most regions adopt heat-using EGS; only Iberia, England, Northern Germany, and Western Poland remain mostly unfavourable to EGS even under high cost reductions (as indicated by hashed regions in Fig. 5). Here, the statistical assessment shows that the distribution of demands plays a significantly larger role in driving EGS market opportunity, with Pearson correlations between 0.4 and 0.6 at high significance (Fig. 6). Surprisingly, the correlation with solar capacity factors is negligible, despite solar thermal being one of the main technologies displaced by heat (co-)generating EGS. Likely, the influence of demand dominates this interaction, overshadowing the more subtle relationship with solar resources. For the spatial distribution of the predictors of EGS market opportunity, see Supplementary Fig. 9 for district heating demands and EGS suitability, and Supplementary Fig. 10 for solar and wind capacity factors.

The lens of cost reductions highlights the technological progress that would be needed for a large share of EGS viability. Supplementary Fig. 6 shows a more system-centric perspective by showing the drilling Capital Expenditure (CAPEX) at which EGS becomes economically competitive. While this perspective aligns largely for electricity-, noticeable deviations appear for heat (co-)generating EGS. In Sweden, Finland, and the Baltics, EGS capacity is built at very high drilling costs of 4500-5500 €kW^−1^ (euros per kilowatt). Conversely, more Central European regions require drilling costs to decrease to 2,000-4000 €kW^−1^. Only minor cost reductions are required to achieve both of these cost levels. However, the difference in CAPEX reveals that EGS adoption in Scandinavia and the Baltics is driven not by geological suitability but by the inefficiency of alternative low-carbon supply.

### Higher Renewable Costs and Larger Roll-Out of District Heating Maximize Market Opportunity

In the following, we consider six sensitivities: Based on the previous analysis with high correlation of EGS adoption with electricity prices and heating demand, we (i) test increased and decreased costs of renewable generation by 20%, (ii) vary the district heating rollout, and (iii) disable the system’s capacity to utilise waste heat for district heating. We further investigate the impact of (iv) enabling flexible operation for EGS, (v) the role played by transmission network capacity expansion and (vi) relaxing emission constraints.

We find the volume of district heating demand to be the most important factor in the rollout of heat-producing EGS. If district heating demand remains around current 2020 levels, rather than experiencing the moderate increases assumed in the base case, the market opportunity of EGS roughly halves, significantly hindering early-stage EGS technology learning. Meanwhile, the market opportunity of electricity generating EGS is more directly enhanced by any factor that improves its competitiveness with wind and solar. The same is true for *flexible EGS*, where the geothermal reservoir is treated as storage, allowing the EGS plant to accommodate renewable intermittency better and thereby improve its market prospects. A more detailed overview of all tested sensitivities is shown in Supplementary Table 1.

**Fig. 7:**
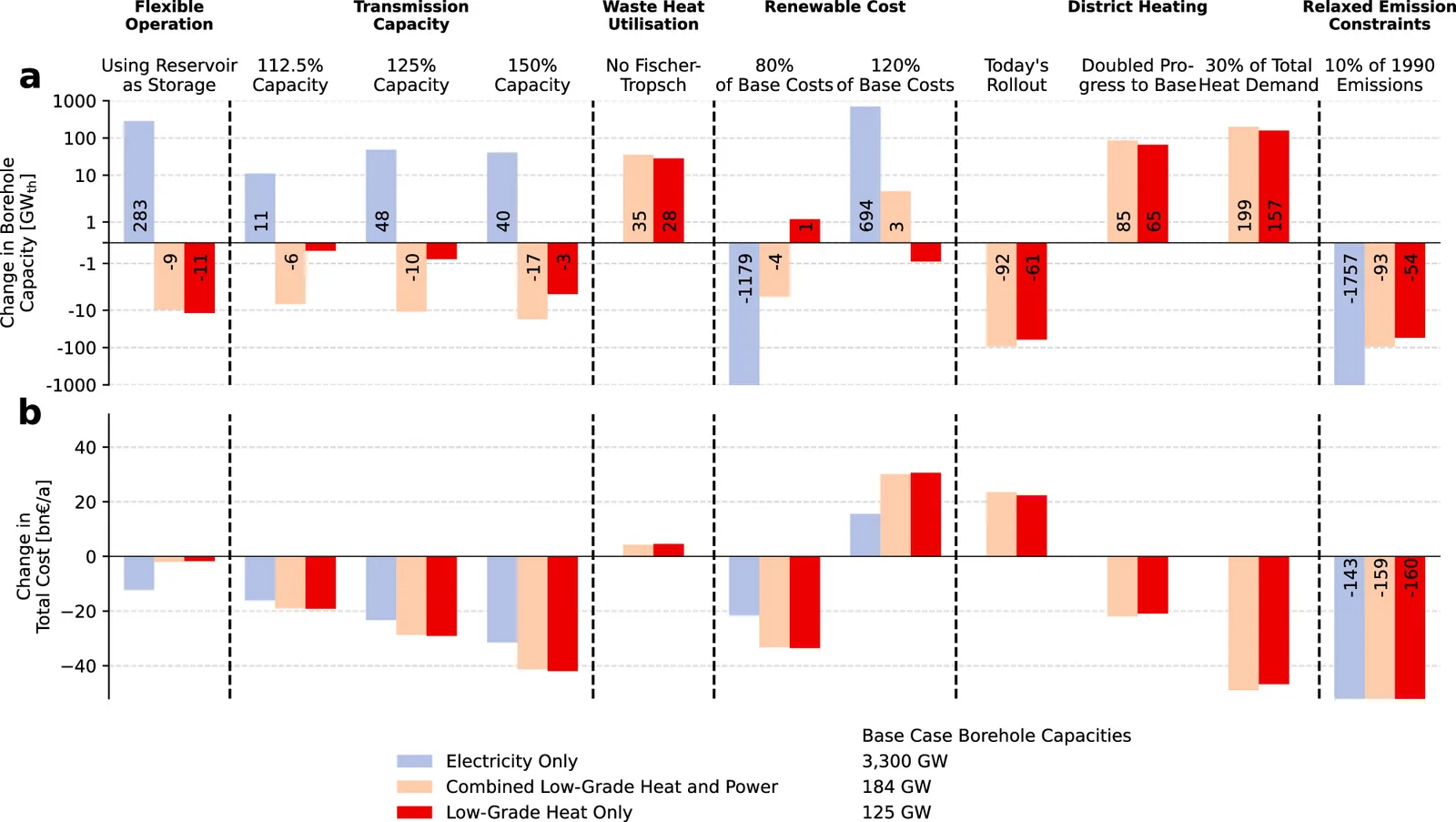
Sensitivity of Enhanced Geothermal Systems (EGS) buildout and system cost to model assumptions. The change in borehole capacity **a** and total system cost **b** is evaluated for given sensitivities relative to EGS base cases, which include EGS under the same system assumptions as outlined in the previous sections. The base cases for heat-generating EGS are scenarios with a cost level of 40% relative to 2020 for heat (co-)generating EGS, and 20% for electricity-generating EGS.

Quantitatively, we find that for electricity-generating EGS, both flexible operation and a 20% increase in renewable costs increase cost-optimal borehole capacity by approximately 1000 GW_th_, compared to a *base* capacity of roughly 3300 GW_th_ in the base case (Fig. 7). A 20% decrease in renewable costs results in nearly matched reduction in borehole capacity. This high sensitivity stems from the often minimal cost-advantage that electricity generating EGS has over other renewables. In contrast, under the same variation of renewable cost, the market opportunity for heat-producing EGS changes by less than 5%, emphasising its substantial cost advantage over the next-best generation technology.

The utility of flexible operation has already been shown in the United States to substantially improve EGS market opportunities^19^, and a similar dynamic is evident here. Flexible operation differs from regular, (assumed to be) dispatchable EGS^17^ by allowing operation at a lower partial load during periods of high levels of intermittent generation and then, after pressure and temperature in the reservoir have increased, generate beyond the plant’s nameplate capacity. However, the resulting increase in reservoir pressure has, in the past, been associated with the risk of inducing seismicity^26^. However, more recent work has shown that gentler ramping of injection rates and other interventions can mitigate this risk^27,28^, and it therefore appears highly likely that present and future EGS plants will be able to modulate back-pressure to temporarily boost generation. The same interventions can also be applied to dispatchable EGS, where sudden ramping can likewise be a risk factor.

With flexibility, we observe an increase of around 300 GW_th_ (around 10%) in borehole capacity, which is lower than the average 30% increase reported in Ricks et al.^19^. This difference could be attributed to various factors, including more pessimistic assumptions about reservoir performance in this analysis or differences in geological or other renewable geographic suitability. For heat-generating EGS, flexible operation decreases borehole capacity. This is because EGS capacity has already saturated the limited district heating demand, and the system uses flexible operation to provide the same energy volume at higher capacity factors. For broader context, the effect of flexible operation on EGS adoption is shown for all evaluated levels of reductions. Operationally, we find that both seasonal and intra-day arbitrage contribute to EGS’ market opportunity (Supplementary Fig. 16), comparable to the behaviour found in Ricks et al.^19^. Both effects are resolved by the model’s 3-hour temporal resolution. However, while these effects are meaningful, they are small relative to the overall magnitude of both EGS capacity and cost reduction (Supplementary Fig. 13).

Further, flexible operation elevates EGS capacity factors close to their technical limit, optimising its dispatch around renewable availability (Supplementary Fig. 17). Thereby, flexible EGS improves on already high capacity factors of inflexible EGS (95 − 99%). We find these differences in electricity generating EGS dispatch mostly to occur in times of low marginal electricity price (Supplementary Fig. 18). During those times, inflexible EGS ceases production while flexible EGS does not. It appears renewable cannibalisation squeezes inflexible EGS out of the market while flexible EGS can shift output to later markets, increasing market value and resulting in the observed increased capacity expansion. In the model, which renewable power source–wind, solar, hydro or EGS–dispatches during abundant renewable availability is determined by their marginal price which might not replicate real marginal prices. Therefore in the real system, dispatch outcomes during abundant renewable generation could be different. However during these times, as the marginal price vanishes, system value vanishes too, and dispatch does not affect these technologies’ investment case. Conversely, flexible EGS’s ability to shift generation out of a long market into a short one is a real effect that enhances EGS market value. The lowest capacity factors are observed for heat-only EGS under extreme cost reductions, where EGS supply saturates heat demand and constrains supply to the profile of demand.

Heat-producing EGS is most affected by the rollout of district heating and the removal of Fischer-Tropsch process waste heat as a competing source of low-grade heat. This work uses a *base case*, in which 30% of the total potential to increase district heating demand to meet 60% of all urban heat demand is met.

For regions that currently have low rollout, this would mean that around 20% of urban heating demand is met by district heating. Conversely, for regions that already have high roll-out, such as the Nordic and Eastern European countries^29–31^, the model essentially assumes today’s levels. The 30% value is therefore mainly relevant for countries with substantial district-heating adoption plans, such as Germany or the UK, where it approximates near- to medium-term targets^32,33^ (see Supplementary Fig. 11 and Methods for details).

It turns out that switching from today’s district heating rollout to a highly optimistic scenario increases cost-optimal borehole capacity by around 200 GW_th_. In this optimistic scenario, it is assumed that 60 % of all urban residential heat demand is met through district heating networks. In many regions, EGS is significantly more cost-efficient than alternative carbon-free heat supplies, making it unsurprising that further demand increases its market opportunity. However, the direct and almost linear relationship between district heating rollout and demand for heat-based EGS is noteworthy, as it directly influences the volume of scarce ground available for technology learning towards electricity generating EGS.

Transmission capacity expansion would likely benefit EGS roll-out by facilitating the transport of EGS-generated power from regions of high geological suitability. By default, however, for two reasons we have opted to use the incumbent grid capacity and explore additional expansion only as a sensitivity. First, allocating grid investment is difficult to align with our modelling approach; the present model does not explicitly assign the relevant share of future grid capacity investment to onshore-offshore grid connection, which constitutes a large share of investment, complicating a precise tracking of the leeway future grid planning should afford the model. We further assume that past trends of onshore grid reinforcement lagging behind renewable roll-out will continue, and therefore examine sensitivities with an additional 12.5%, 25%, and 50% grid capacity (expressed in gigawatt-kilometres, GWkm), in line with expectations for the magnitude of future changes^34^. This addition results in a 1–3% capacity increase for electricity-generating EGS and a 5% decrease for combined-heat-and-power EGS, leading us to conclude that our findings are largely robust to future transmission-capacity expansion, also under a  + 50% expansion; in that case, EGS build-out even slightly decreases relative to a  + 25% expansion, likely because other renewables similarly profit from improved siting enabled by higher transmission capacity.

Default model runs assume a fully carbon neutral system. As a low-carbon technology, this constraint benefits EGS, but we find that it does so in a somewhat surprising way. In a sensitivity that relaxes the carbon-neutrality target, allowing emissions up to 10% of 1990 levels (instead of 0%), we find EGS capacity expansion reduced by roughly 1800, 90, and 50 GW_th_ for electricity-, co-generating, and heat-only EGS, respectively–eliminating about half of the market opportunity. However, in all three cases we find that this is not the result of stronger competition for the demands EGS serves; rather, its role as a facilitator of deep decarbonisation diminishes. Specifically, the low-grade heat that EGS supplies inexpensively lowers the cost of direct air capture, while electricity demand is amplified by H_2_ electrolysis–both downstream effects of the perfect-decarbonisation assumption in the default setup.

We have further considered competition from alternative dispatchable, carbon-neutral sources, in particular gas generators with Carbon Capture and Storage (CCS). However, because gas CCS is not constrained by geographic suitability, shares dispatchability characteristics with EGS, and is subject to large cost uncertainty, we opt for a simpler LCOE comparison (Supplementary Fig. 15). A straightforward LCOE calculation for a 30-year lifetime, 7% discount rate, 47% efficiency, and 50% capacity factor–together with CAPEX assumptions of 2000-2900€kW^−1^^35,36^–places such dispatchable generation at values comparable to EGS, with additional sensitivity to gas price. In a low-gas-price scenario (15€MWh^−1^^37^) gas CCS could generate carbon-neutral power at a cost similar to that required for EGS adoption in the model. Under a more pessimistic price assumption (30€MWh^−1^^38^), however, gas CCS appears largely uncompetitive with the EGS capacities adopted by the model. Similar to EGS, gas CCS is mostly at the demonstration stage, and substantial uncertainties remain regarding its feasibility–particularly its currently much lower-than-expected capture rates^39^, with the additional cost of rectifying these shortcomings still unclear. In summary, gas generators with CCS could plausibly compete with EGS as a source of firm, carbon-neutral power; however, the combined uncertainties surrounding both technologies complicate any direct comparison, and future work should track the performance of gas CCS demonstration projects relative to those for EGS.

## Discussion

Our results quantify how cost reductions in EGS influence its market potential, both in terms of overall volume and the sectors it can supply. These costs are expressed relative to 2020 levels estimated in Aghahosseini and Breyer^2^. However, in light of the recently demonstrated advanced stimulation techniques at the Blue Mountain and UtahFORGE sites, both conducted after 2020, it appears plausible that as of 2025 already, 20-25 % cost reductions have already been achieved^17^.

We find that in scenarios of drilling cost reductions of less than around 60% (in the following referred to as *first phase*), heat (co-)generation is crucial for EGS to be competitive. However, in scenarios with cost reductions of more than 60% of current costs (in the following referred to as *second phase*), a tipping point is reached, after which electricity-generating EGS can compete directly with wind and solar generation, unlocking an exponentially larger market opportunity. We refer to the periods before and after this tipping point as the first and second phases of EGS deployment.

The first phase is crucial with regard to EGS technology learning. Our results show that district heating demands enable around 100 GW_th_ of EGS capacity expansion that is already cost-competitive with alternative supply in a carbon-neutral system. As such, this capacity requires only minor financial aid, as subsidies would primarily be needed to account for its carbon neutrality. Based on this, we estimate that a technology learning rate of around 20% during this phase (Supplementary Fig. 2) could lead to self-sustaining growth into a second phase.

In the second phase, electricity generating EGS competes directly with wind and solar generation. Competition in electricity markets entails a significantly larger market opportunity, estimated at approximately 250 GW_th_ (equalling 25–30 GW_el_) if costs decrease to around 40% of 2020 levels, and about 900 GW_th_ when reduced to 20%. This finding broadly aligns with previous results for electricity-generating EGS under optimistic drilling-cost scenarios reported by Longa et al.^4^, which project around 25 GW_el_ of capacity, while also offering additional context on its spatial distribution and the conditions under which this market opportunity arises. The findings also align with a comparison between current European electricity prices^40^ and the EGS LCOE used here from Aghahosseini and Breyer^2^. In 2020–2021, central European prices have been around 30–60€MWh^−1^, for which, to compete, EGS would need cost reductions more extreme than the scenarios assessed here (see Supplementary Fig. 15). However, both the European near-complete embargo of Russian gas since 2022 (since then, electricity prices have settled between 50 and 150€MWh^−1^^40^) and carbon phase-out have led to higher electricity prices^23^. As of March 2026, the German baseload futures for 2027–2030 are in the range between 70 and 100€MWh^−1^^41^. As a result, the roughly 60% cost reduction, deemed required here, would likely hold in the real system, both in the gas-reliant current system and a future renewable one.

It remains unclear whether the EGS cost reductions evaluated here can be realised. The range of cost trajectories analysed reflects the values deemed plausible in Aghahosseini and Breyer^2^, where technology learning is driven by projected exponential growth in EGS deployment and a 7.5% cost decrease for each doubling of installed capacity. A qualitative comparison to alternative cost estimates suggests that our trajectories are conservative relative to some optimistic benchmarks. For example, a recent IEA report^42^ suggests that, under an optimistic trajectory, EGS could reach LCOEs as low as 50 USD MWh^−1^ by 2035 and 35 USD MWh^−1^ by 2050. While these values are global and are therefore not directly comparable to our Europe-focused results, they lie below most of the LCOEs assessed in the present study (see Supplementary Fig. 15). For context, the bespoke market expansion case with a 60% cost reduction for electricity-generating EGS corresponds to LCOEs of roughly 75–120 €MWh^−1^. The relatively modest learning rate (7.5% per capacity doubling) underpinning our input data and therefore our assessed cost reductions^2^ is likely responsible for this difference. Note that this is distinct from the in this paper estimated technology learning rate required for EGS roll-out to be self-sustaining (i.e., where market opportunity at one level of cost reduction results in the technology learning that unlocks the next level of cost reduction) of 20 − 25%–a much steeper requirement than assumed in the input data (of 7.5 %)^2^, but comparable, for instance, to reported solar PV learning rates (20 − 30%^24^). Technology-learning spillovers from outside Europe would reduce the value estimated here; conversely, as shown in the Sensitivities, less stringent emissions constraints in Europe or a business-as-usual district heating roll-out could significantly increase it.

Further, the data used here assumes idealised learning from increased global uptake but does not yet consider the additional potential of EGS to supply heat. Our present analysis–showing that most early, high-cost EGS market opportunity lies in the heating sector–therefore highlights a learning potential that is under-emphasised in the existing literature. However, our baseline 2020 estimate of EGS cost^2^ anticipated a roughly 25 % reduction by 2025–a decline that data from active demonstration sites suggests has indeed materialised^17^.

Additional revenue streams via electricity market frameworks could ease the need for drastic cost reductions by rewarding EGS for its ability to provide firm electricity^43^. The modelling used here implicitly assumes an energy-only market. However, the ongoing renewable roll-out can create unfavourable market conditions for dispatchable resources (through highly unstable spot prices), especially before demand-side flexibility has caught up^44^. To ensure the availability of such resources, countries have introduced mechanisms, such as capacity markets, that reward firm generators for complementing variable renewables^45^. Such incentives would benefit electricity-generating EGS. Additional revenue streams could also arise for EGS if field trials demonstrate sufficiently rapid dispatch adjustments to support grid stability through frequency response^43^.

In the context of heating, European regulation is already moving to shape district-heat markets in ways that support the roll-out of EGS. For instance, district-heat markets are regulated local monopolies, and market access for new heat generators differs from the electricity sector. However, recent work shows that, across varying degrees of national regulatory stringency, renewable generation sources can still diffuse into district-heat markets^46^. Moreover, policy support for heat-generating EGS is gaining momentum: the European Union’s Renewable Energy Directive 2023/2413 prohibits district-heating systems  > 25 MWth from monopolistic gate-keeping and encourages instruments such as heat-purchase agreements^47^. In addition, national policies already exist in some district-heating–heavy countries—for example, Sweden’s District Heating Act includes conditional third-party access for injecting heat into networks, and cross-country reviews document substantial heterogeneity in analogous access rules across other EU member states^48^. As of summer 2025, the German government is preparing legislation to accelerate geothermal project planning^49^.

Further policy support should protect investors against the risks inherent in engineering geothermal reservoirs and developing exploratory, first-of-a-kind projects, similar to the de-risking strategies employed during the early roll-out of wind generation. Such policies are increasingly being implemented across European countries, including several large heat markets such as France, the Netherlands, Poland and Germany ^50–54^. These incentives could serve as a powerful lever to break the investment trap, whereby high geothermal costs hinder widespread adoption and limited adoption in turn slows technology learning.

The market potential opportunity discussed here exceeds the cumulative installed capacity of European EGS projects many times. Even under the more conservative cost-reduction scenarios, the identified 20-30 GW_th_ opportunity exceeds the roughly 100 MW_th_ operating EGS capacity in Europe^55–59^ by orders of magnitude. Assuming each well doublet provides around 10 MW_th_, realising that capacity would require thousands of wells. Drilling times vary considerably; for instance, Utah reached a depth of 5 km in 16 days^60^, whereas a Bavarian project in 2003 required almost 150 days^61^. As a back-of-the-envelope calculation, one drilling rig with an average well-drilling time of 60 days could add about 30 MW_th_ per year (assuming one well doublet provides  ~ 10 MW_th_). At that rate, developing 30 GW_th_ would demand roughly 1000 rig-years, i.e. on the order of 100 years with 10 rigs. While Europe hosts around 120 active drilling rigs overall^62^, stakeholder interviews suggest that only a small subset is suitable for deep onshore geothermal drilling; for example, a recent supply-chain consultation reported only  ~ 13 geothermal-capable rigs across Europe^63^. This underscores both the practical importance of rig and service-sector availability in any large-scale EGS roll-out and the critical role of technology and skill transfer from the oil-and-gas industry.

Our analysis highlights the stark differences in the spatial distribution of market opportunities for heat- and electricity-generating EGS. However, we find that only a small number of factors classify regions into four distinct roles with regard to EGS’ pathway to market.

First are potential early EGS adopters. Surprisingly, in this case, the primary driver for adoption is not geological suitability but rather the high cost of alternative sources of carbon-neutral energy for district heating schemes. As a result, Scandinavia and the Baltics could see substantial investment in EGS at current cost levels of 4500-5500€${{{{\rm{kW}}}}}_{{{{\rm{th}}}}}^{-1}$. The second role includes the majority of heat-generating EGS adopters, such as France, Benelux, Southern Germany, and the Balkans. In these regions, a market opportunity of approximately 10-30 GW_th_ is already accessible at current cost levels, increasing to about 100 GW_th_ if EGS costs decrease by up to 40%. At this stage, EGS outcompetes most alternative carbon-neutral district heating options, making the demand for district heating itself the primary predictor of EGS adoption. The third kind of region sees investment into electricity-generating EGS if drilling costs were to reduce by around 60%. These regions, which include Central-Eastern Europe, mostly see EGS adoption due to high geological suitability. The remaining regions-namely Iberia, England, Northern Germany, Southern Italy, and Western Poland-are either geologically unsuitable, rich in renewables resulting in low energy costs, or lack district heating infrastructure. As a result, they provide limited opportunities for EGS adoption at any evaluated level of cost reduction.

The results have further implications beyond Europe, where district- and low-temperature industrial heat demands are substantially larger. For instance, China and Russia each exceed 4500 TWh of annual district heating demand, while the rest of the world accounts for more than 1500 TWh^64^. Importantly, the geospatial data layers needed to extend such assessments beyond Europe are increasingly available via publicly accessible geothermal mapping platforms (e.g., GeoMap), which combine subsurface favourability with surface-relevant layers for heating and industrial applications^65^. However, a comprehensive global analysis that explicitly simulates techno-economic competition between geothermal and alternative heat-supply options under network and operational constraints remains to be carried out. Adoption of EGS on a meaningful scale in these regions could boost European adoption since cross-regional knowledge spillovers appear plausible: many advances relevant to next-generation geothermal (e.g., drilling and subsurface engineering methods) are disseminated through the open scientific literature, while additional know-how embodied in specialised equipment and services can diffuse through internationally operating supply chains^66^.

In summary, district heat and low-temperature industry demands, under a carbon-neutral constraint, could offer EGS a geographically widespread market opportunity, even at current or close-to-current drilling cost. Given subsequent cost reductions, this could be followed by a later concentration in regions with high geological suitability around Central Europe via electricity generating EGS. We also observe that the spatial distribution of existing EGS projects^67^ leaves many of the market opportunities identified here untapped. While some of this potential should be realised as carbon constraints increasingly reveal the identified distribution of EGS opportunity, policy interventions could help accelerate this process.

Our analysis quantifies the sensitivities of EGS market opportunities and finds them to be primarily influenced by district heating rollout and the future cost of renewables. In decreasing order of importance, secondary factors include waste heat utilisation, flexible operation, and transmission grid reinforcement.

Regarding district heating, this work assumes a base scenario in which district heating will experience some limited rollout beyond current levels. For comparison, we also test a scenario where district heating remains at current levels and find that the market for high-cost EGS decreases drastically, from 200 GW_th_ to around 100 GW_th_. Moreover, the spatial distribution of this reduced demand further exacerbates the disparity between the spatial distribution of heat-producing and electricity-producing EGS market opportunities. This is because, without further rollout, current district heating demand is even more concentrated in Eastern European and Scandinavian countries, while the market opportunity for electricity generation remains in more central regions. Therefore, in the EGS context, district heating expansion not only provides a means to expand demand for EGS, but also serves as a vehicle to prevent regional lock-in effects. Conversely, if the roll-out of district heating surpasses current projections, particularly in countries with large heat markets but limited existing networks, the potential market for EGS could expand substantially. If for instance, 30 % of all heating demand was met by central supply, it likely would increase cost-optimal EGS borehole capacity by around 150 GW_th_ relative to our base scenarios of moderate progress (Supplementary Fig. 11). In short, whether European district-heating deployment falls short of or exceeds expectations, the implications for the EGS market would be significant, and therefore an attempt to track EGS’s future market requires precise tracking of district heating development.

EGS has a unique interaction with transmission capacity. When generating electricity only, it exhibits similar dynamics to other renewables: if transmission capacity expansion is allowed, the model partially pays for grid reinforcement through lower expenses in generation capacity, enabled by better geographic suitability^68^. However, when EGS is used to meet heat demands, this interaction no longer applies. For generation of combined low-grade heat and power, enabling transmission expansion reduces its market opportunity. Due to the difficulty of transporting heat, EGS cannot relocate to take advantage of the spatial flexibility offered by larger transmission capacity. As such, the common association of transmission expansion benefiting renewable energy holds for EGS only to a very limited extent, as other renewables can typically also exploit the additional spatial flexibility. If large cost reductions fail to materialise, transmission expansion should not be considered a catalyst for EGS deployment. The analysis shows that EGS derive much of their value from enabling very deep decarbonisation: In the baseline model, a zero-emissions constraint allows EGS to provide low-grade heat, which lowers the cost of direct air capture, and electricity that supports large-scale hydrogen electrolysis. When the carbon cap is relaxed to 10 % of 1990 levels, EGS capacity falls by half-not because of new competitors, but because the system no longer needs those decarbonisation services. Potential competition from gas turbines with CCS is assessed via a simple LCOE comparison (see Results and Supplementary Fig. 15). At low gas prices gas-CCS can match EGS costs, but under more pessimistic price assumptions it becomes uncompetitive; moreover, real-world capture rates lag expectations, adding further uncertainty. Thus gas-CCS could eventually vie with EGS for firm, carbon-neutral power, but the substantial techno-economic unknowns surrounding both technologies warrant close monitoring of forthcoming demonstration projects.

Beyond the modelling limitations detailed in the Methods, EGS roll-out could face societal constraints that are not captured by the modelling framework. Public opposition to EGS increased notably after fracking-related events in which induced seismicity activated existing fault lines, leading to noticeable ground shaking, widespread building damage, and project abandonment-as in Basel, Switzerland, in 2006^69^, and even more severely in Pohang, South Korea, where a magnitude 5.5 earthquake was triggered^70^. To avert such events, frameworks have been proposed in both Europe and the United States to accompany EGS roll-out; they include seismic-risk pre-screening and the monitoring of risk indicators during drilling, stimulation, and operation^28,71,72^. Across Europe, anti-seismicity protocols span national risk-screening frameworks (e.g., Switzerland), permit-embedded traffic-light systems (e.g., France and the Netherlands), and industry guidance that informs case-by-case permitting (e.g., Germany); by contrast, in the UK induced-seismicity management has largely been handled through local planning conditions only^73–78^.

These protocols are likely to lead to only a minor overall cost increase or, irrespective of such protocols, to discourage EGS deployment in urban settings. On the one hand, these cost additions are small relative to the cost range evaluated here, particularly for heat-generating EGS, which appears to hold a sizeable advantage over alternative carbon-neutral heat supplies. Future work could, however, examine the implications of public opinion restricting EGS adoption to rural areas. To interpret the findings in light of these potential additional costs, we suggest reading, for example, Fig. 3 with an additional shift along the *x*-axis representing the corresponding cost increase.

Beyond additional costs, future seismic events-which may occur again-could deal a substantial blow to EGS roll-out and introduce hurdles that are hard to quantify in monetary terms. Even in its current state, past events have already raised significant practical barriers to EGS deployment. Moreover, public opinion remains largely unformed about EGS owing to its niche status^79^; by contrast, fracking is widely known and a majority of people-particularly within liberal urban cohorts-hold an unfavourable view of it^80^. In this context, rigorous seismic monitoring and environmental-impact assessments are essential to maintain public trust during roll-out. However, despite these measures, a non-zero probability of an EGS-induced seismic event-detrimental to EGS’ public perception-will remain and, similar to excessive regulatory hurdles, cast additional uncertainty over the technology’s future. Further limitations are discussed in the Methods.

In summary, this work outlines the potential trajectory for the large-scale adoption of EGS in a carbon-neutral, multi-sector energy system for Europe. In such a system, we find that EGS could already be cost-competitive for heat generation in regions with highly suitable geology and district heating networks or low-temperature industry heat demands. If drilling costs reduce to around 40% of 2020 levels, EGS that generates only electricity would become cost-competitive with other renewable resources, potentially enabling large-scale adoption in Europe of 20–100 GW_el_ power capacity. To achieve this, supportive policies have already been adopted over recent years and are crucial for further EGS development, yet additional bottlenecks may still impede progress if left unaddressed. Crucially, ensuring open third-party access to district heating networks and stimulating sufficient heat demand are foundational steps that have not yet been rolled out across the European Union. At the same time, carbon markets or targeted subsidies should reward the carbon neutrality of EGS, as an increasing number of countries are beginning to do. Regulators must also rigorously enforce practices that mitigate seismic risk, because any induced tremors could provoke fracking-style public backlash and render EGS politically untenable. Investment instruments and loans should socialise early geological and first-of-a-kind risks to shield investors from poor reservoir performance. Finally, streamlining permitting and other regulatory procedures would cut delays and lower entry barriers for EGS developers. While current European drilling-rig capacity is moderate, a rapid EGS scale-up could strain rigs, expertise, and the wider supply chain, making proactive upscaling essential.

## Methods

### Energy System Model

We conduct our analysis in the sector-coupled model of the European-level energy system PyPSA-Eur^81^. It optimises investment and operation of energy generation, transport, storage and conversion over a full year with a three-hourly resolution, covering demands from the power, heat, transport, industry and agriculture sectors while maintaining a neutral carbon balance. We provide a brief overview of the central features but refer to Neumann et al.^81^ for a more detailed discussion.

To reflect the energy system’s geographical context in terms of renewable potentials, energy transport and demand, the model represents Europe in 72 zones. All region and country geometries shown in maps are derived from Natural Earth data (public domain; https://naturalearthdata.com). Each zone is assigned a weather-based time series of renewable maximum generation output per installed capacity, based on the open-source Python tool *atlite*^82^. For the transmission grid, we have opted, based on the small impact observed in our sensitivity analyses, to prohibit the model from expanding the line capacities in an effort to reduce computational overhead.

In terms of technology roll-out assumptions, the model year is set to 2050 to align with the carbon-neutral target. Specifically, we assume 85 % electrification of road transport, modifications to steel and aluminium production processes, a high degree of building retrofits to reduce space-heating demand, and a complete fuel switch in shipping from oil to methanol. We further assume that renewable-energy costs decline in line with Danish Energy Agency projections for 2030^83^, representing an average reduction of roughly 25 % relative to 2020 levels.

Crucially, the model features EGS-relevant power, district heating, and low-temperature industrial heat demands. Electricity demand is increased relative to today’s levels due to the electrification of other sectors, leading to a total demand of around 11PWh (Supplementary Fig. 1). Heating demand aggregates to a yearly total of 3PWh, only 0.6PWh of which is district heating, with the remaining demands being either rural (with insufficient population density for communal heating) or non-residential sectors. The model can also increase the volume of heat supplied to district heating nodes and use the excess heat for direct air capture. To estimate the magnitude and distribution of heat demands, the model employs the methods outlined in Zeyen et al.^84^.

Yearly total heating demand per region is disaggregated from country-level figures based on the *Integrated Database of the European Energy System* (JRC-IDEES)^85^, Eurostat^86^, complemented by additional figures for Switzerland^87^ and Norway^88^. These aggregate numbers are then spatially distributed by population density. Further, the NUTS3 dataset^89^ is used to assign the share of the population living in urban areas per region. For the demand classified as urban, district heating shares are assigned based on today’s district heating shares at the country level^90^. However, further district heating rollout is widely seen as an ingredient of a cost-efficient residential heat supply in Europe, and as such the model adds our assumptions of future district heating demand to these demands^32,33^. The degree of progress assumed is quantified through the share of urban residential district heat demand that is met by EGS. Our default assumption is a progress of 30% between today’s rollout and the maximum plausible share of district heat. This share is assumed to be 60% of urban residential demand. An overview of country-level district heating demand levels is given in Supplementary Fig. 11. Additionally, the model considers low-temperature industrial heating demands, which are lumped together with district heating demands but, in magnitude, are much smaller. Whenever the present text refers to *district heat*, it includes this additional demand for low-temperature industry. The incumbent technologies that supply these demands are biomass, gas boilers, CHPs, solar thermal or waste heat from fuel synthesis. Additionally, we assume thermal storage to be available for demand shifting.

In terms of investment and operational costs, the model faces the conflict between the single-year optimisation horizon and the need to account for the value of technologies accrued over their lifetimes, which exceed a single year many times over^81^. Therefore, the model is provided with annuitised yearly payments using the annuity factor given as equation (1)

1 $$\alpha=\frac{1-{(1+\tau )}^{-n}}{\tau }$$

where *n* is an asset’s lifetime and *τ* is the discount rate, assumed to be 7% throughout the study. In total, capacity expansion is costed as equation (2)

2 $$(\alpha+\,{\mbox{FOM}})\cdot {\mbox{CAPEX}}\,$$

where FOM is the Fixed Operation and Maintenance Cost [%] and CAPEX is the capital expenditure [€kW^−1^].

Finally, the model in this work optimises, under constraints, investment decisions for all energy generation and conversion technologies, including EGS. This is in contrast to an alternative study design that could simulate varying technology deployments, and assess economic competitiveness through their effect on system cost. Therefore, the optimal deployment per technology in the present study is an endogenous variable, and all capacity that gets built is exactly revenue-adequate, i.e. profits are zero by design^91^. In this framing, the techno-economics of technologies offered to the model already account for the relevant incentives, most importantly for carbon-neutrality through the carbon budget constraint, and the respective level of EGS cost reductions.

### Modelling Enhanced Geothermal Systems

We begin with a discussion of the data inserted, independent of the modelling framework, and only then move to its representation in PyPSA-Eur. We use techno-economic data from Aghahosseini and Breyer^2^ to represent cost and the spatial aspects of geological suitability in the model and refer to that paper for a more elaborate methodological description on how the numbers are derived, but provide a brief overview here. Building on the protocol proposed in Beardsmore et al.^92^, the 1^∘^ × 1^∘^ gridded data provided in Aghahosseini and Breyer^2^ estimates, for each square and based on geological data-maps, the cost-optimal EGS depth by optimising the trade-off between higher costs through deeper drilling and lower surface plant costs achieved by higher temperatures of geological resources.

Further, Aghahosseini and Breyer^2^ makes assumptions about future cost reductions resulting from technology learning. Based on the projected exponential increase in EGS capacity towards 2050, paired with a technology learning rate of 7.5% at each doubling of built capacity, the paper asserts the cost reductions used in the present paper (see Fig. 1, reaching a maximum cost reduction factor of 5 in 2050). To make our results more intuitive, we decouple the cost reduction from the specific year assigned in Aghahosseini and Breyer^2^ and instead express it as a factor relative to costs estimated for 2020. While Aghahosseini and Breyer^2^ is supplemented only by country-level data, courtesy of the author, we were able to access the underlying gridded data for our experiments. Each region in our model was assigned an EGS potential based on the overlay with its geometry.

The technology-learning assumption in Aghahosseini and Breyer^2^ is implemented as a blanket cost reduction of the total EGS capital cost, rather than as separate learning rates for individual sub-components, and that assumption is inherited into the present paper. In particular, the learning factor is multiplied with the aggregated CAPEX (see Supplemental Material Eq. S1 in Aghahosseini and Breyer^2^), which sums well drilling & completion costs and surface power-plant costs (i.e. the ORC plant), alongside other project components. This approach enables readers to map the cost reduction they are interested in, regardless of the component responsible for the reduction, to the findings here. Neither Aghahosseini and Breyer^2^ nor our implementation models seismicity-related costs endogenously; such effects can only be represented via an assumed net CAPEX adjustment.

Analogously, the present paper translates the learning assumption into an aggregated representation: we scale the full EGS cost pipeline, i.e. drilling and the ORC surface plant, by a single cost-reduction factor relative to the 2020 baseline. The results therefore represent net project cost reductions irrespective of which specific technological advances (e.g. improvements in drilling equipment, reduced seismicity-related costs etc.) ultimately deliver them. Policy risk-sharing could change the effective private cost baseline; we note this in Limitations.

Further, for EGS configurations that forgo electricity generation and deliver heat only, we must isolate the ORC-related cost component to subtract it. We assume a default ORC cost in 2020 (see below) and hold the ORC share of total cost constant across the imposed cost-reduction levels. This is consistent with Aghahosseini and Breyer^2^, who attribute a similar learning potential to the ORC plant as to drilling (7.5% per doubling of installed capacity). However, the evidence to support this claim is limited. By contrast, Ricks and Jenkins^93^ assumes stronger learning in drilling than in the ORC plant, though both learning rates are higher than those assumed here (15% for drilling and 10% for ORC).

We include EGS in the energy system model in three different modes each with different energy outputs - electricity, combined low-grade heat and power or low-grade heat only. All three share the same subsurface modelling, but differ in the surface plant’s configuration.

Considering the surface plant first, for electricity generation, we model an organic Rankine cycle (Organic Rankine Cycle (ORC)), which is a standard choice for electricity generation at low temperatures, utilising the lower boiling point of the organic fluid relative to water^2,7,19^. For the efficiencies of the ORCs, we use estimates based on regression of existing plants to be around 7 − 8% at approximately 105^∘^C and plateau at approximately 15% from 200^∘^C onward, which could further increase through modifications to the plant^7,94^. Our model works with a default value of 12% efficiency, aligning with temperature assumptions in the underlying geothermal potential data (see Subsection *Data on Geological Suitability*). Later in this subsection, we discuss the implications of our results for EGS operating at other efficiencies. While ORCs are a mature technology, uncertainty in terms of realistic cost assumptions remains. Various sources assess plant costs to be between 1300€${{{{\rm{kW}}}}}_{{{{\rm{el}}}}}^{-1}$ and 2000€${{{{\rm{kW}}}}}_{{{{\rm{el}}}}}^{-1}$^2,95–97^. In our model, we use EGS capital expenditure of 1500€${{{{\rm{kW}}}}}_{{{{\rm{el}}}}}^{-1}$, which aims to reflect an average of these observed values paired with modest technology learning. The ORC is also assumed to have an average technical availability that allows it to deliver, on average, 93% of nameplate capacity^19^. In general, we assume that the ORC’s ramp rates are large relative to the temporal resolution of the model, effectively imposing no constraints on dispatch between time steps^98^.

For combined low-grade heat and power operation, this setup is enhanced according to Eyerer et al.^67^. In this case, the model can modulate the working fluid flow between an electricity-generating cycle (ORC) and a heat-exchanger cycle, which transfers heat content to meet the required demand temperatures. In this setting, Eyerer et al.^67^ report a broad operational range down to 15.3% partial load. Based on this, we assume that our modelled absence of partial load constraints introduces only a minor error. Using an estimation made in Frey et al.^96^, we cost the additional infrastructure to supply district heat at 25% of the ORC cost. While this value, i.e., the cost of the heat integration, can vary due to a number of factors, we assume a default value here to avoid cluttering the analysis. PyPSA-Eur, without EGS, employs a 15% loss of district heat during distribution. Our model concatenates this with an EGS heat generation loss of 5%.

During capacity expansion, we assume a fixed ratio between heat and electricity capacity generation. The fixed ratio is given by their efficiencies *η*_dh_/*η*_el_ ≈ 8, with the two individual values taken from Tester et al.^7^ and Frey et al.^96^. However, we do not constrain the ratio between heat and electricity generation during dispatch. This decision is informed by Eyerer et al.^6^, which finds that EGS plants in Germany typically generate both heat and power but achieve far higher full-load hours for heat than for electricity, indicating that the systems modulate between the two.

Finally, for the generation of low-grade heat only, the ORC part is removed, leaving only the heat exchanger cycle operating at an assumed 95% efficiency. Our modelling uniformly sets heat generation temperatures to 150 ^∘^C, which helps streamline our analysis but necessitates additional steps to generalise the results to other production temperatures. At 150 ^∘^C working fluid temperature, which is a typical value in existing geothermal projects albeit low for projected future projects, the electricity generation efficiency is around 12%^7^. The formulation of our capital cost (€kW^−1^) allows us to remain agnostic to different temperatures. However, this approach misses how different temperatures change the distribution of CAPEX between the geothermal well and the ORC plant, which can complicate the utilisation of our findings in real EGS projects. To ensure interpretability, we recommend the following protocol to infer the implications of our findings for an arbitrary drilling CAPEX *c*_*x*_ at an arbitrary temperature *x*. Statements made in the paper are formulated through drilling CAPEX ${c}_{15{0}^{\circ }{\mbox{C}}}$, expressed in units €${{{{\rm{kW}}}}}_{{{{\rm{th}}}}}^{-1}$. To interpret the respective findings in terms of the total plant cost, i.e., aggregating subsurface and surface parts, we calculate the overall CAPEX expressed in €${{{{\rm{kW}}}}}_{{{{\rm{el}}}}}^{-1}$ as equation (3):

3 $${c}_{{{{\rm{total}}}}}=\frac{{c}_{15{0}^{\circ }{\mbox{C}}}}{{\eta }_{15{0}^{\circ }{\mbox{C}}}}+{c}_{{{{\rm{orc}}}}},$$

where ${\eta }_{15{0}^{\circ }{\mbox{C}}}$ is the ORC plant’s power generation efficiency at 150^∘^C, which is 12%. At a different temperature level, electricity is generated at efficiency *η*_*x*_, and the respective total cost follows from the analogue of equation (3). It follows that for a geothermal well generating heat at temperature *x*, the relevant CAPEX to compare against in our analysis is given as equation (4)

4 $${c}_{15{0}^{\circ }{\mbox{C}}}={c}_{x}\frac{{\eta }_{15{0}^{\circ }{\mbox{C}}}}{{\eta }_{x}}.$$

Note that this is only relevant for power-generating EGS, since low-grade heat usages convert incoming heat at extremely high efficiencies to the required output temperatures.

While the surface part differs, all three share the same model of borehole operation. The subsurface part of the EGS plant is split into the injection and production wells, with capital expenditure assigned equally between them. We take the perspective of the whole energy system, and as such, the geothermal well is treated as a firm generator of heat, assumed to be capable of modulating its energy output on timescales smaller than the model’s temporal resolution of three hours. If flexible operation is activated, we additionally treat the geothermal reservoir as storage to represent the build-up of pressure and temperature when backpressure is applied to the production well^19^. The physics of that process are not included in the model, but they are assumed to justify the modelled storage capacity. In this, we assume the reservoir can accommodate a build-up of 24 hours, which can be discharged through a generation surplus of up to 25%. This temporal extent of flexible EGS is slightly larger than the temporal extent of flexible operation tested in the field^99^, but smaller than in similar studies^19^. Finally, we model the efficiency of the geothermal heat extraction cycle similarly to Ricks et al.^19^, as a function of local deviations from the ambient temperature at the plant’s design point temperature. The magnitude of the changes in technical availability is informed by measurements taken at the DORA-1 geothermal plant in Turkey^100^.

Investment costs for EGS are processed as outlined in equations (1) and (2). Marginal costs are omitted for EGS plants, as they are assumed to be small relative to the investment costs.

Finally, throughout this work EGS is assumed to operate as a dispatchable generation source by default. Although originally considered a baseload technology, more recent studies show that pausing and resuming production is feasible^19,43,101^. We note that dispatchable operation is distinct from flexible operation, in which the production well ceases to flow while the injection well continues, building reservoir pressure and enabling a boost in generation once production resumes^97^. The economic benefits of such flexible operation are analysed in the sensitivity analysis.

### Technology Learning Rate

The techno-economic inputs in our work are taken from Aghahosseini and Breyer^2^. There, the authors exogenously assume an (exponentially) increasing future roll-out along with a learning rate, and infer the resulting cost reduction that scenario would see. The resulting cost levels are what we use here. Our work in contrast, finds EGS roll-out endogenously as a function of exogenously imposed cost reductions. Therefore, the inserted cost-reduction levels are no longer tied to the technology learning mechanism in Aghahosseini and Breyer^2^. Instead, we merely inherit the range of cost reductions deemed plausible by that paper. Given the pair *(cost reduction, deployment)*, we then infer the learning rate required for deployment to be self-sustaining. This implied learning rate is shown in Supplementary Fig. 2, and this section provides the context for that figure.

We use a standard formulation of technology learning, where a learning rate quantifies the technology cost reduction per doubling of installed capacity of that technology^102^. Starting with the *power law* given as equation (5)

5 $$C(x)={C}_{0}\cdot {x}^{-b}$$

where *C*(*x*) refers to the cost at cumulative installed capacity *x*, *C*_0_ to the initial costs, and *b* to a learning exponent, we obtain the learning rate as equation (6)

6 $${{{\rm{LR}}}}=1-{2}^{-b}.$$

It is this learning rate that is referred to in the text. In the following, the *cost reduction factor* is defined as equation (7)

7 $$\,{\mbox{cost reduction factor}}=\frac{{{\mbox{cost}}}_{{{{\rm{original}}}}}}{{{\mbox{cost}}}_{{{{\rm{reduced}}}}}}\hspace{2.22144pt},$$

i.e. a cost reduction factor of 2 refers to halved costs. For the initial capacity *C*_0_, we use the capacity installed by the model without cost reductions, i.e., at cost reduction factor 1^103^. From this, at each level of cost reduction *y*, we obtain the respective learning rate using the respective installed capacity by the model *C*_*y*_, as equation (8),

8 $$b=\frac{\ln (1/y)}{\ln ({C}_{y}/{C}_{0})}\hspace{2.22144pt},$$

and derive the learning rate LR from equation (6). Note that *y* is larger than 1, which we accommodate by making it the denominator.

### Limitations

We believe our work is subject to a few main limitations. These are the simplified nature of our EGS modelling and potential estimation, the approximations made in the assignment of EGS costs to different regions, and the uncertainty about realistic energy transition pathways.

With regard to the first limitation, the physics that govern heat generation from the geothermal well are neglected in this work to accommodate the computational overhead of the model. Every EGS project is subject to uncertainties regarding the actual reservoir performance once drilling is completed, and rectifying subpar performance may require expensive geological engineering. In both the EGS potential dataset provided by Aghahosseini and Breyer^2^ and this work, this risk factor is not priced into the drilling cost, leading to a potential underestimation of real costs. These cost factors are omitted since they depend on geological characteristics, the analysis of which exceeds what can feasibly be addressed in this study. Moreover, the geology of EGS itself creates further uncertainty in terms of generation over the plant’s lifetime, including sudden structural changes in the reservoir that can lead to capacity reductions^99^. We assess this limitation to be within the standard approximations of large-scale models, which neglect similar nuances for other generation technologies. When comparing results from our work to real-world estimates, we urge the reader to keep this in mind and consider comparing this work against risk-adjusted real-world costs. While subsidies and policy interventions to mitigate this investment risk exist (see Discussion), the model implicitly assumes that such mechanisms are-and remain-available to shield investors.

However, the data underpinning the estimations of suitability for EGS used here are also subject to uncertainties beyond those in analogous datasets for other technologies. This is mainly due to subsurface temperature data, which is often found to differ from pre-drilling estimates. This issue is highlighted, for instance, by two datasets of subsurface temperature in the United States that exhibit substantially varying spatial distributions of values^19,104^. Therefore, our results should be interpreted as preliminary indications based on current knowledge of EGS suitability but may deviate from the true EGS opportunity.

Our cost-reduction scenarios are implemented as a multiplicative factor on aggregated EGS CAPEX (well-field/drilling and, where applicable, the ORC surface plant), following Aghahosseini and Breyer^2^ where learning is applied to total CAPEX (Supplementary Eq. S1). Consequently, neither Aghahosseini and Breyer^2^ nor our implementation endogenously allocates costs between individual mechanisms, such as higher drilling expenditures from deeper wells versus lower expenditures associated with induced-seismicity mitigation; these effects can only be reflected insofar as they change the total project CAPEX captured by the applied factor. Moreover, local regulation could change the effective project cost and thereby complicate comparison to this work. This could for instance apply in case of partial public coverage of unsuccessful exploratory drilling, as implemented in parts of Germany^105^. If this is the case, the relevant comparison to our thresholds is against the developer’s expected, policy-adjusted cost.

A related approximation concerns the split of costs between the subsurface and surface parts of the plant. In heat-only EGS, the capital cost of the organic Rankine cycle (ORC) is removed, leaving only the drilling-related component. The cost split between drilling and the ORC depends on borehole depth and reservoir temperature. However, the modelling assumes a uniform geothermal temperature of 150 ^∘^C (following Aghahosseini and Breyer^2^) and therefore does not account for spatial variation in this split. In highly suitable regions the model likely overestimates the drilling cost share and thus slightly overstates total EGS costs, while the opposite holds in less suitable areas. This effect is most relevant in the low- to moderate-cost-reduction cases, where EGS faces stronger competition.

This work also neglects the gradual nature of transition pathways in the European energy system. The uncertainty regarding how that transition may manifest motivated us to instead opt for a model with the common denominator of carbon neutrality and let the model optimise the energy system’s configuration. The assumption of carbon neutrality lends a competitive advantage to low-emission technologies like EGS, which makes our findings less relevant in the current system. Especially in the heating sector, identified here as crucial for early EGS, marginal prices are much lower than those seen by the model, which primarily assumes conventional heating. However, we argue that, given the energy system’s trajectory towards carbon neutrality, locating the analysis within this framing is the most sensible option, as it allows the model to weigh technologies against one another in the context most likely to reflect future conditions. Further assumptions about the future system, such as technology rollout in fuel synthesis or the adoption of district heating, were qualified through sensitivity analyses (see Results).

Large-scale optimisation models are inherently subject to two kinds of noise: numerical optimisation imprecision and uncertainty arising from modelling assumptions and parameter choices. Numerical noise is negligible here, as PyPSA-Eur is solved to standard feasibility/optimality tolerances of order 10^−6^ (relative), far below the magnitude of the reported  < 1–6% system-cost changes^106^. By contrast, parametric and structural uncertainty (e.g. techno-economic inputs, spatial/temporal aggregation, and omitted constraints) may be comparable to a few percent in long-horizon energy system optimisation. We therefore interpret the reported savings as conditional comparative results within a consistent modelling set-up, and complement them with sensitivity analyses to assess robustness to perturbations.

Our analysis does not model waste-heat supply from CCS-equipped thermal plants or from nuclear facilities as competing heat sources for district heating. The future roll-out and cost of both of these technologies are subject to large uncertainties, and therefore their integration into the model under blanket cost assumptions is confounding rather than insightful. To illustrate this, we have included an illustrative LCOE-based comparison for gas power plants with CCS (Supplementary Fig. 15), highlighting the overlap in their uncertainty bands due to both investment and fuel costs. For nuclear, the potential for district heating is strongly constrained by siting and political feasibility, both of which are difficult to capture in a purely economic model. However, both technologies constitute realistic alternatives to geothermal district heating, and future work could provide an adequate treatment of their potential future competition.

Further approximations had to be made when assigning estimated EGS costs to regions of the network. Subsurface suitability can have a higher spatial resolution than the network, requiring the grouping of different potentials into a single potential assigned to one region. In the present work, each region is assigned the average EGS cost of all patches per region, weighted by heat potential. This method can mix regions of extreme suitability with regions of lower suitability, potentially missing low-cost EGS. While this is imprecise, it is a reasonable option in light of the low spatial granularity of the underlying dataset, which has a resolution of 1^∘^ × 1^∘^, complicating regional assignment to begin with. Future, more granular datasets would capture high-suitability sweet spots more accurately and could enable a demand-side assessment that checks whether demand centres coincide with such sweet spots and feeds these overlaps into the optimisation model.

### Ethics & Inclusion Statement

All input datasets are publicly and freely accessible from their original providers, under open licences or terms permitting free use for research (e.g., ENTSO-E hourly load data, the ERA5 reanalysis provided by the Copernicus Climate Change Service, and Eurostat statistics, each reusable with attribution). The complete modelling framework (code, processed data and result visualisations) has been deposited on Zenodo, with the code under an MIT licence and third-party input data remaining subject to the terms of their original providers, enabling reuse and extension by the European research community.

### Usage of Large-Language Models

The authors of this work have used large language models to improve the grammatical correctness, word choices, and clarity of the text.

## Supplementary information

Supplementary Information

Transparent Peer Review file

## Data Availability

The model results generated in this study have been deposited on Zenodo under https://doi.org/10.5281/zenodo.21778394^107^. The data are openly available under a CC-BY-4.0 licence. This includes both the input data of the modelling, and the modelling outputs as they were used as input for plotting. Third-party input datasets (e.g., ENTSO-E hourly load data, the ERA5 reanalysis provided by the Copernicus Climate Change Service, and Eurostat statistics) are freely available from their original providers under their respective terms of use.

## Code availability

The code to reproduce the experiments is available on GitHub at https://github.com/LukasFrankenQ/egs_market_integrationand has been archived on Zenodo under https://doi.org/10.5281/zenodo.21778394^107^. We further refer to the documentation of PyPSA (https://pypsa.readthedocs.io) and PyPSA-Eur (https://pypsa-eur.readthedocs.io). The code is released under an MIT licence.
