# Supplementary material for: Market Integration Pathways for Enhanced Geothermal Systems in Europe
Source: Nat Commun. 2026 Oct 16;17:11666. doi: 10.1038/s41467-026-77893-3 (PMC13760043; doi:10.1038/s41467-026-77893-3)
Supplement: Supplementary file 1 [file 41467_2026_77893_MOESM1_ESM.pdf]

Supplementary Information

Market Integration Pathways for Enhanced Geothermal Systems in Europe

Lukas Franken[1,2,*], Elisabeth Zeyen[2], Orestis Angelidis[3], Tom Brown[2], Daniel Friedrich[1]

[1] School of Engineering, Institute for Energy Systems, University of Edinburgh, United Kingdom

[2] Department of Digital Transformation in Energy Systems, Technische Universität Berlin, Germany

[3] James Watt School of Engineering, University of Glasgow, United Kingdom

[*] Correspondence: lukas.b.franken@gmail.com

Supplementary Items

Supplementary Figure 1	Energy demand, generation and nodal system cost without EGS
Supplementary Figure 2	Cost-optimal borehole capacity and required learning rate
Supplementary Figure 3	Capacity changes of non-EGS technologies, electricity-only EGS
Supplementary Figure 4	Capacity changes of non-EGS technologies, combined heat and power EGS
Supplementary Figure 5	Capacity changes of non-EGS technologies, low-grade heat EGS
Supplementary Figure 6	Borehole capital expenditure at which EGS contributes 10% of demand
Supplementary Figure 7	Model regions
Supplementary Figure 8	Injection well borehole capacities per region
Supplementary Figure 9	District heating demand and EGS suitability
Supplementary Figure 10	Renewable capacity factors
Supplementary Figure 11	District heating demand for different rollout assumptions
Supplementary Figure 12	Correlation of nodal features with low-grade heat EGS buildout
Supplementary Figure 13	Borehole capacity and system cost under flexible operation
Supplementary Figure 14	Capacity changes when flexible operation is enabled
Supplementary Figure 15	Levelized cost comparison against gas generation with carbon capture
Supplementary Figure 16	Operational pattern of flexible EGS
Supplementary Figure 17	Capacity factors for different EGS modes
Supplementary Figure 18	Dispatch versus marginal price for static and flexible EGS
Supplementary Figure 19	Marginal energy prices for different EGS cost reductions
Supplementary Table 1	Sensitivities tested relative to the base case
Supplementary Table 2	Lower confidence intervals of Pearson *R* values, electricity-generating EGS
Supplementary Table 3	Upper confidence intervals of Pearson *R* values, electricity-generating EGS
Supplementary Table 4	Lower confidence intervals of Pearson *R* values, CHP-generating EGS
Supplementary Table 5	Upper confidence intervals of Pearson *R* values, CHP-generating EGS
Supplementary References	

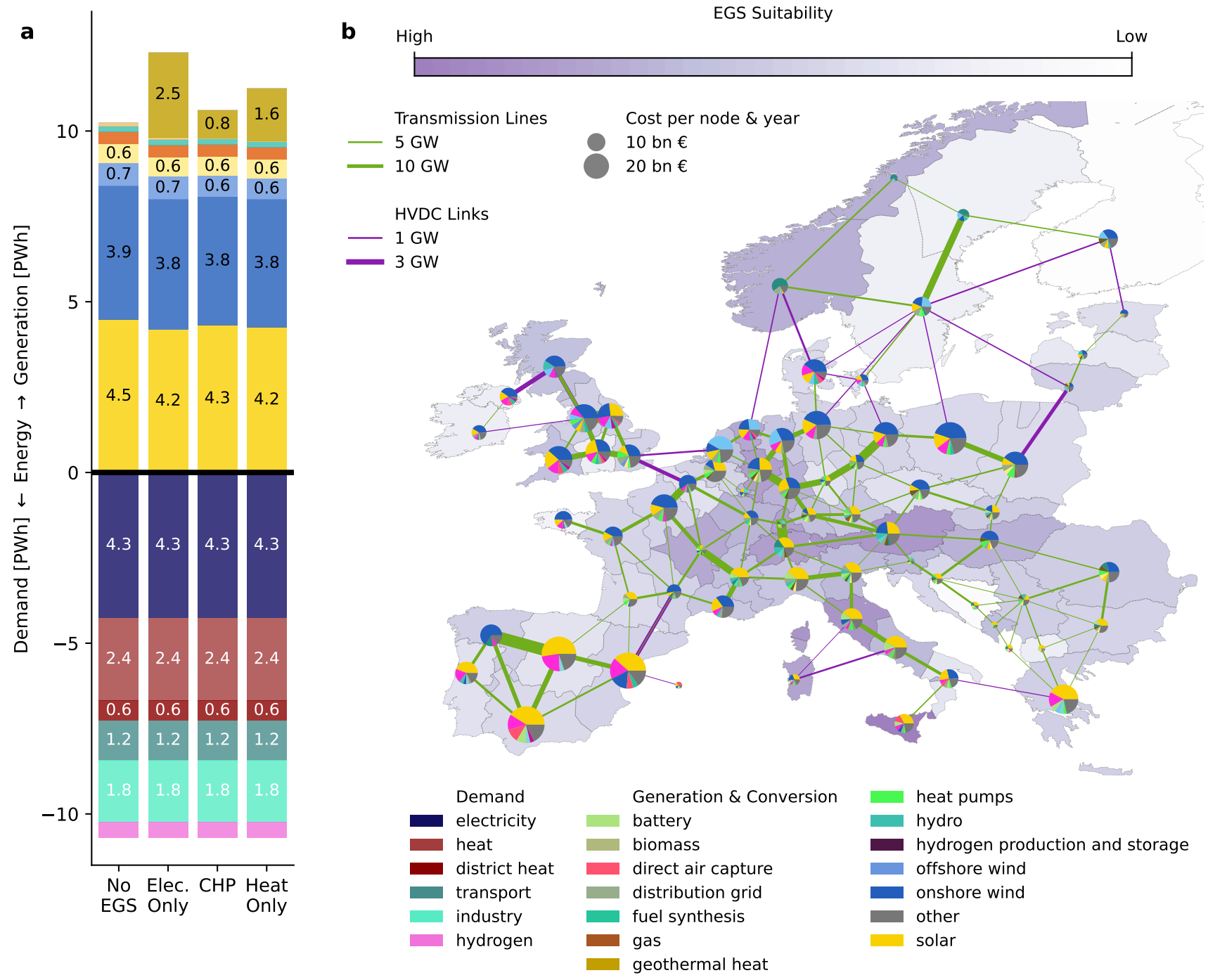

Supplementary Figure 1: **a** Energy demand and generation for model runs without Enhanced Geothermal Systems (EGS), and with EGS generating (i) only electricity, (ii) combined low-grade heat and power and (iii) only low-grade heat. **b** Annual cost of energy generation, conversion and storage for a model without EGS for each node. Note that for electricity only generating EGS, the volume of geothermal heat looks exaggerated as the low efficiency of power generation is yet to be applied. EGS suitability is taken from Aghahosseini and Breyer [1] and mapped to the model geometries. CHP: combined heat and power.

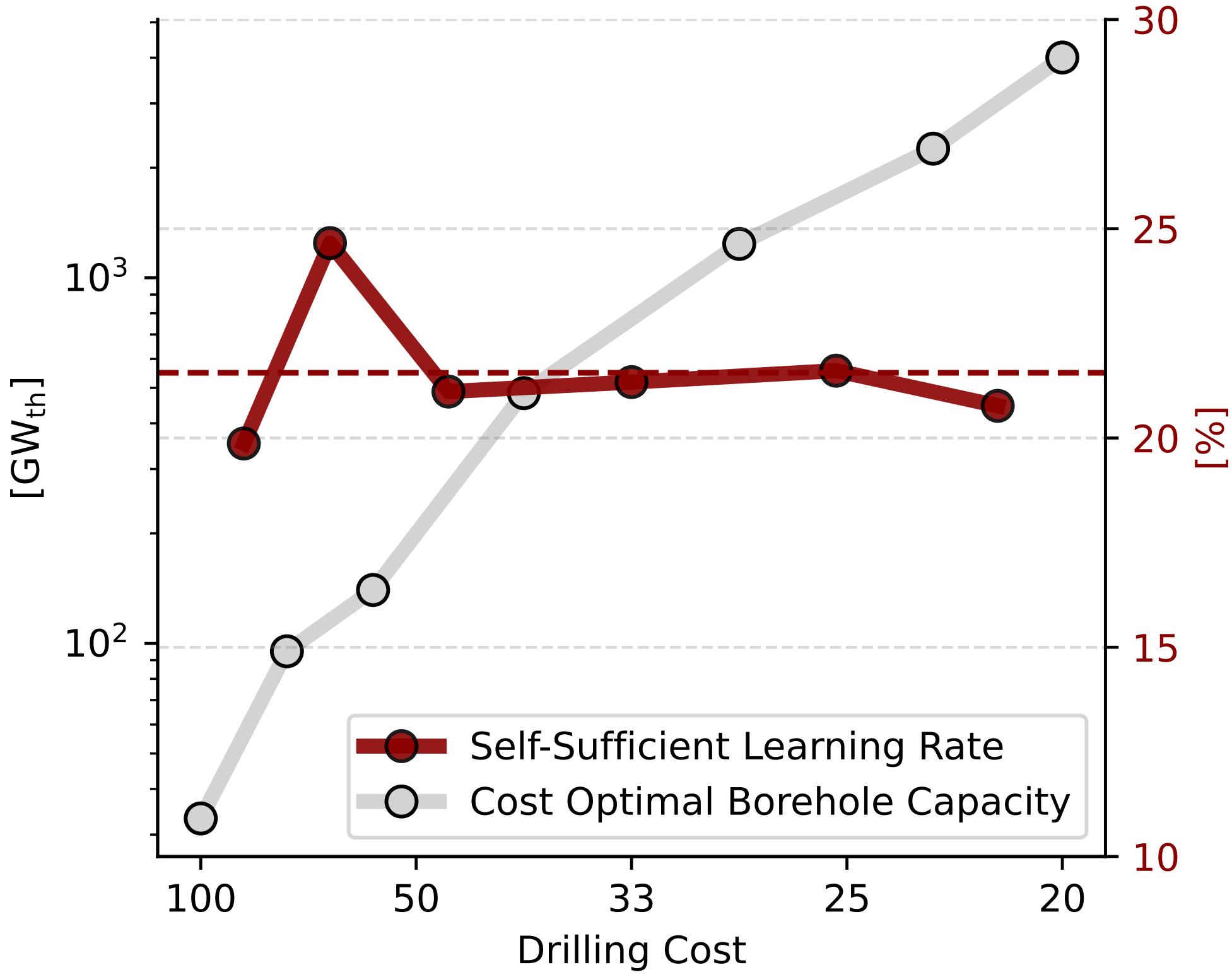

Supplementary Figure 2: Cost-optimal borehole heat capacity, summed for Enhanced Geothermal Systems (EGS) producing electricity only and for combined low-grade heat and power, is evaluated at various levels of cost reduction and learning rate required for EGS itself to enable sufficient technology learning.

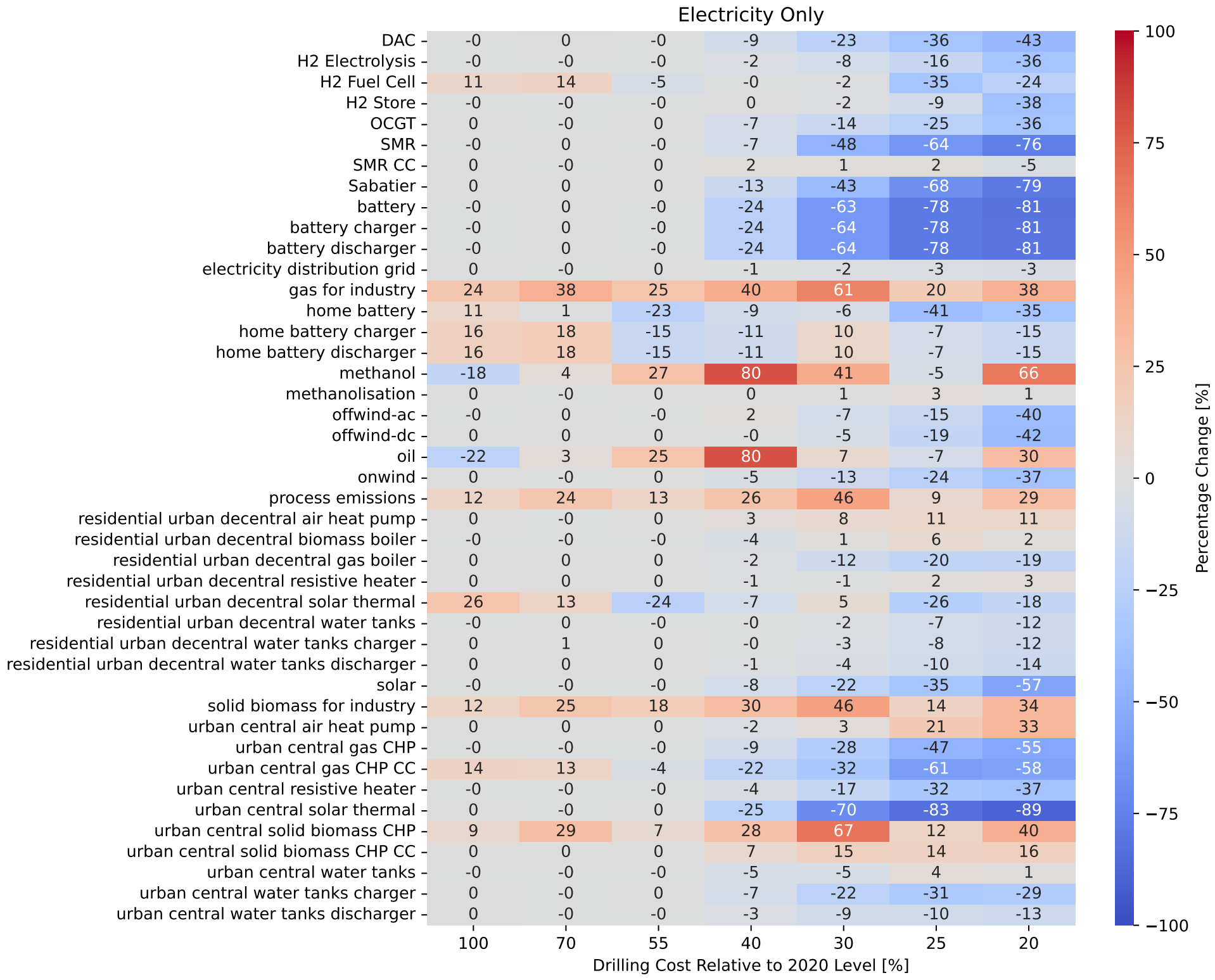

Supplementary Figure 3: Percentages of changes in installed capacities of non-EGS technologies when electricity-only generating Enhanced Geothermal Systems (EGS) is added at different levels of cost reduction. The figure does not show technologies that supply service sector or rural heat demands which we assumed to not be eligible to EGS supply, and therefore of minor relevance to the study. CHP: combined heat and power; CC: carbon capture; OCGT: open-cycle gas turbine; H_2: hydrogen.

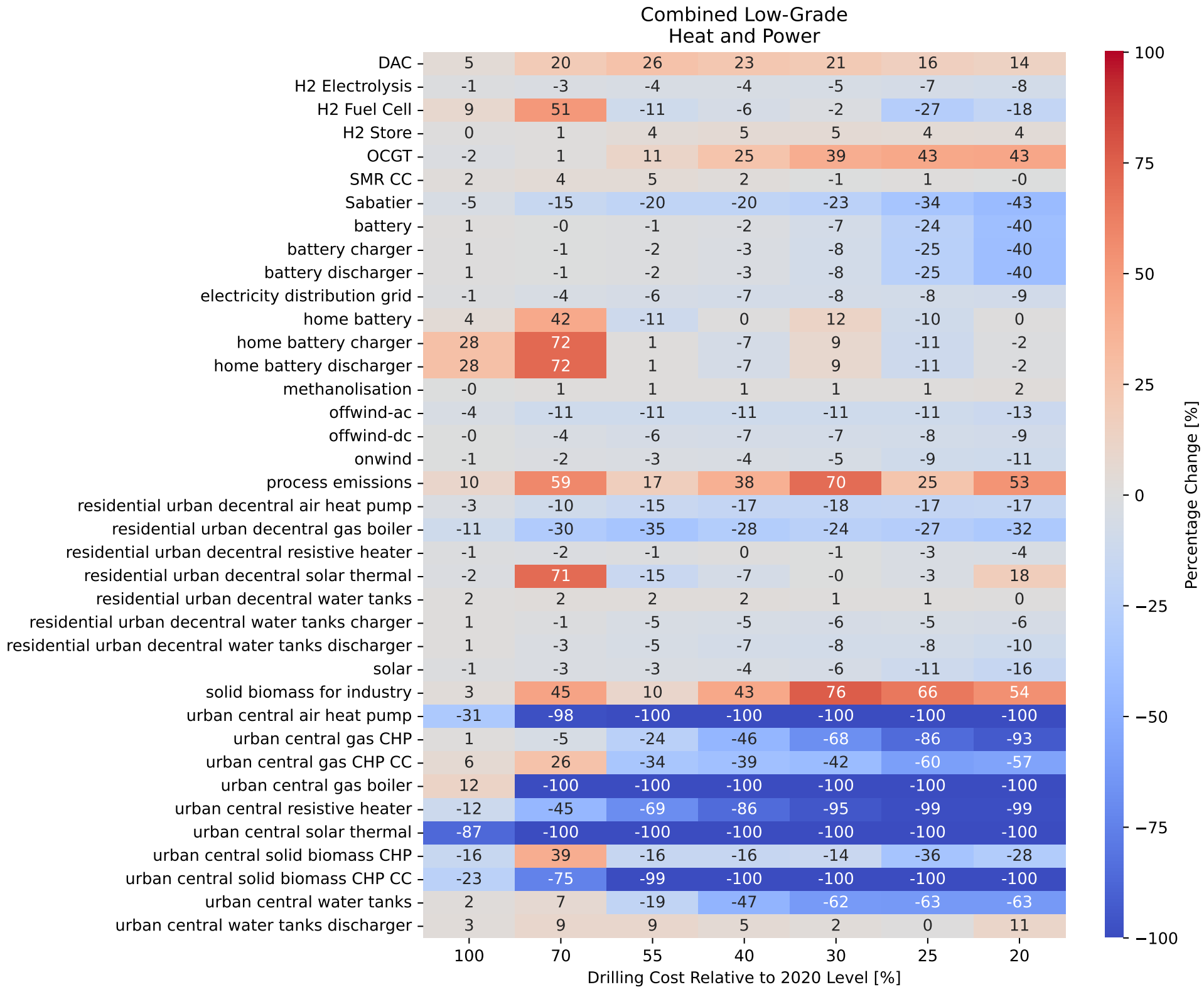

Supplementary Figure 4: Percentages of changes in installed capacities of non-EGS technologies when *Combined Low-Grade Heat and Power* Enhanced Geothermal Systems (EGS) is added at different levels of cost reduction. The figure does not show technologies that supply service sector or rural heat demands which we assumed to not be eligible to EGS supply, and therefore of minor relevance to the study. CHP: combined heat and power; CC: carbon capture; OCGT: open-cycle gas turbine; H_2: hydrogen.

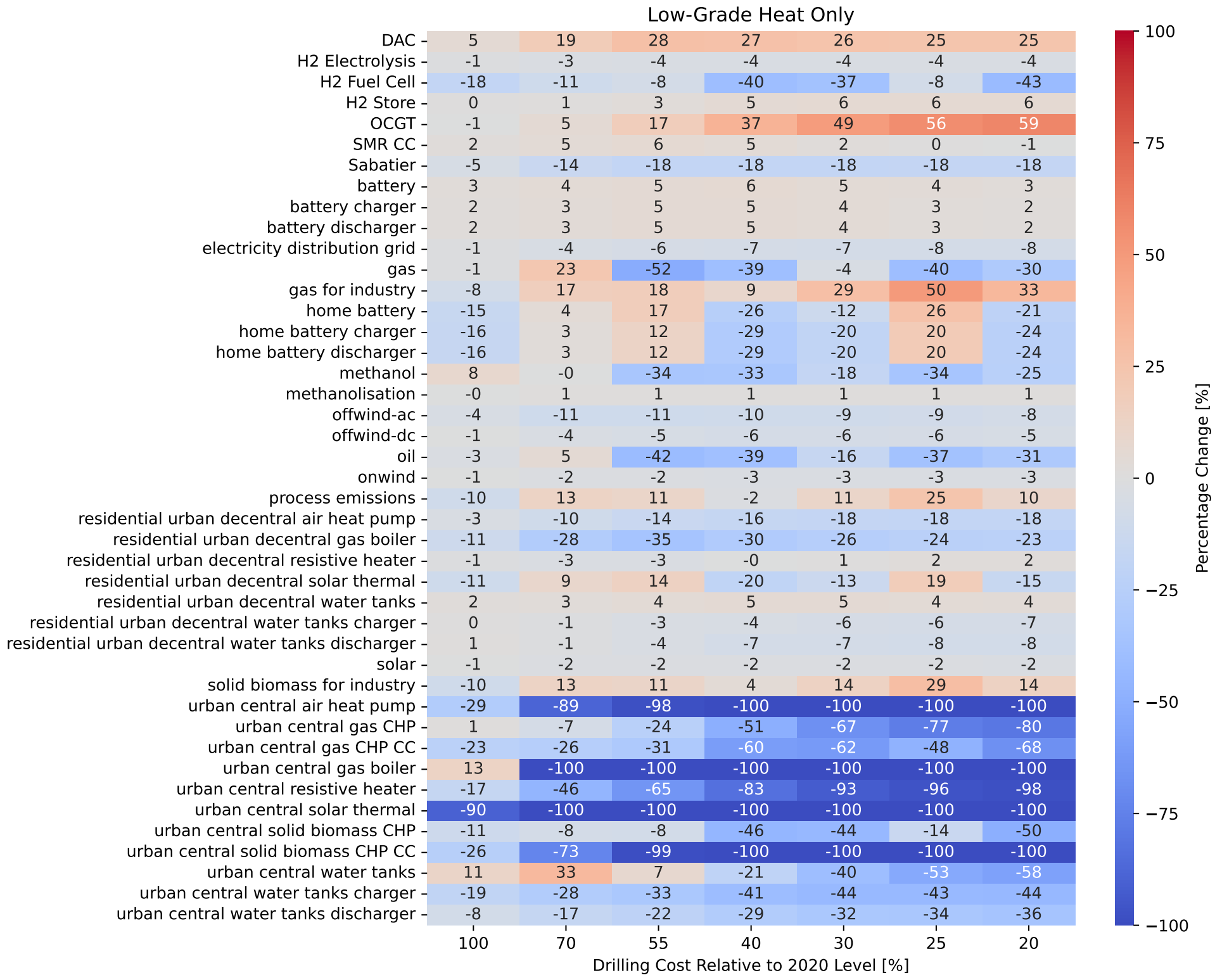

Supplementary Figure 5: Percentages of changes in installed capacities of non-EGS technologies when *Low Grade Heat* Enhanced Geothermal Systems (EGS) is added at different levels of cost reduction. The figure does not show technologies that supply service sector or rural heat demands which we assumed to not be eligible to EGS supply, and therefore of minor relevance to the study. CHP: combined heat and power; CC: carbon capture; OCGT: open-cycle gas turbine; H_2: hydrogen.

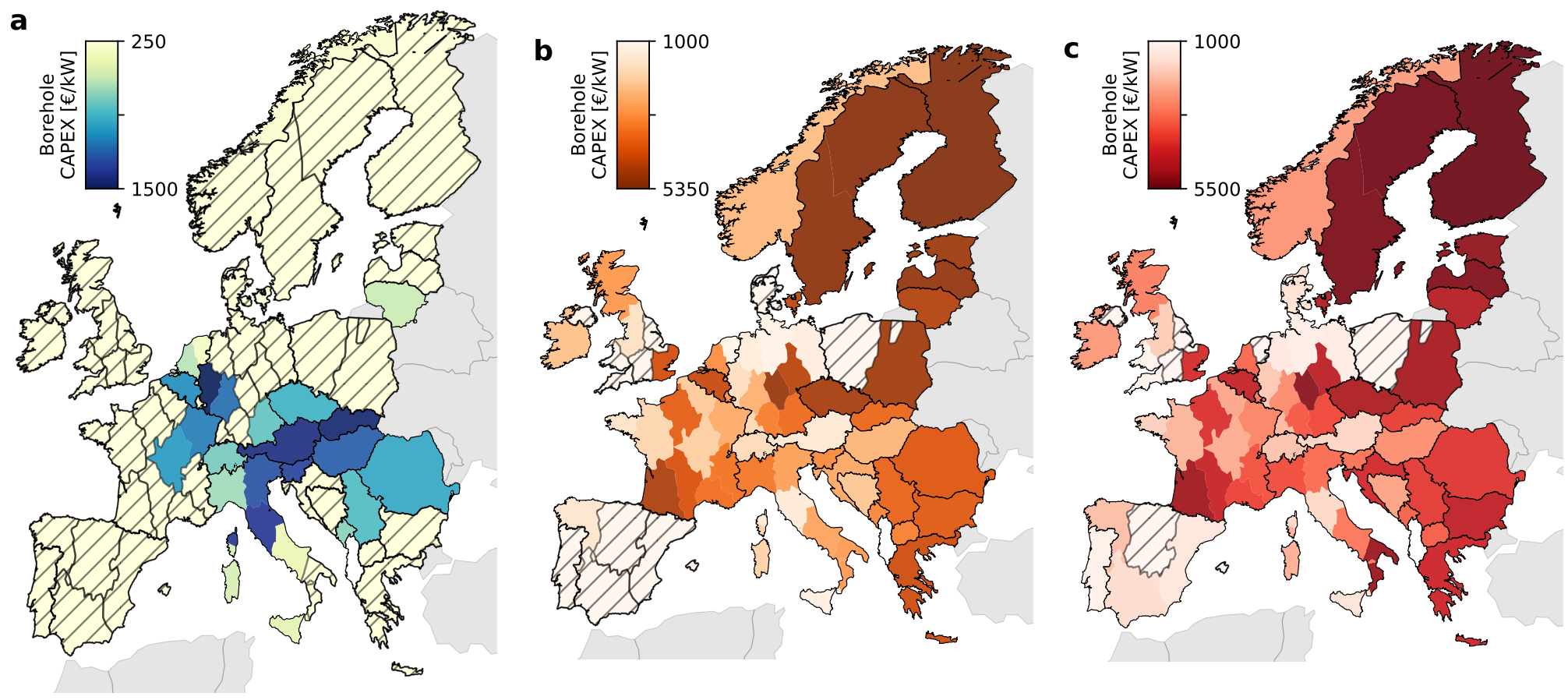

Supplementary Figure 6: Borehole capital expenditure (CAPEX) at which Enhanced Geothermal Systems (EGS) contributes 10% of the respective demand per region. **a** for *electricity only*, **b** for *combined low-grade heat and power* and **c** for *low-grade heat only*. Note the different spans of the respective colourmaps. The hashed region saw no EGS deployment in any modelled cost reduction factor. We note that Albania is empty because the underlying dataset did not contain EGS data for that region.

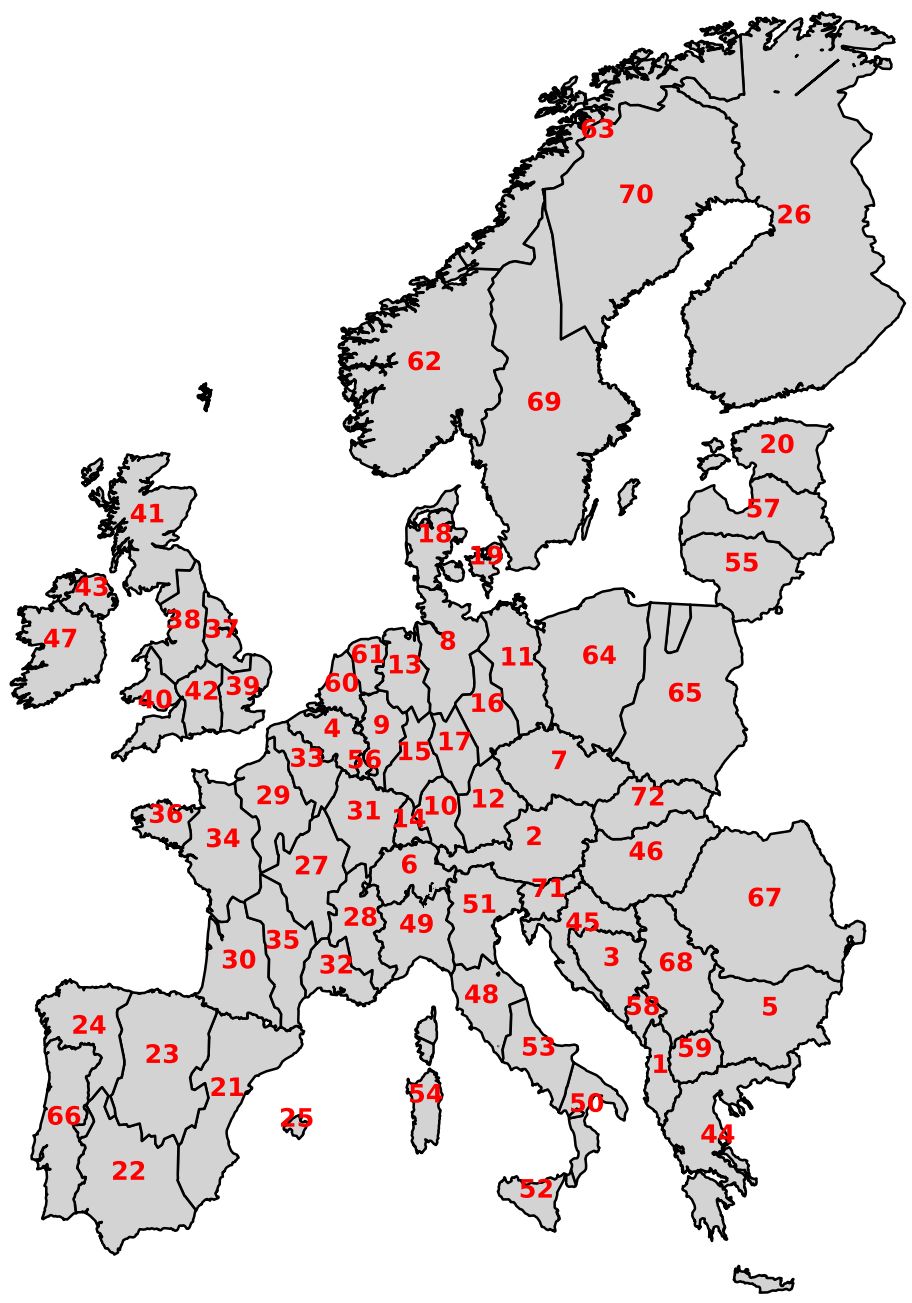

Supplementary Figure 7: All regions modelled in the network with mock-names. The regions result from a within-country clustering method inherent to the model[2,3], not administrative regions.

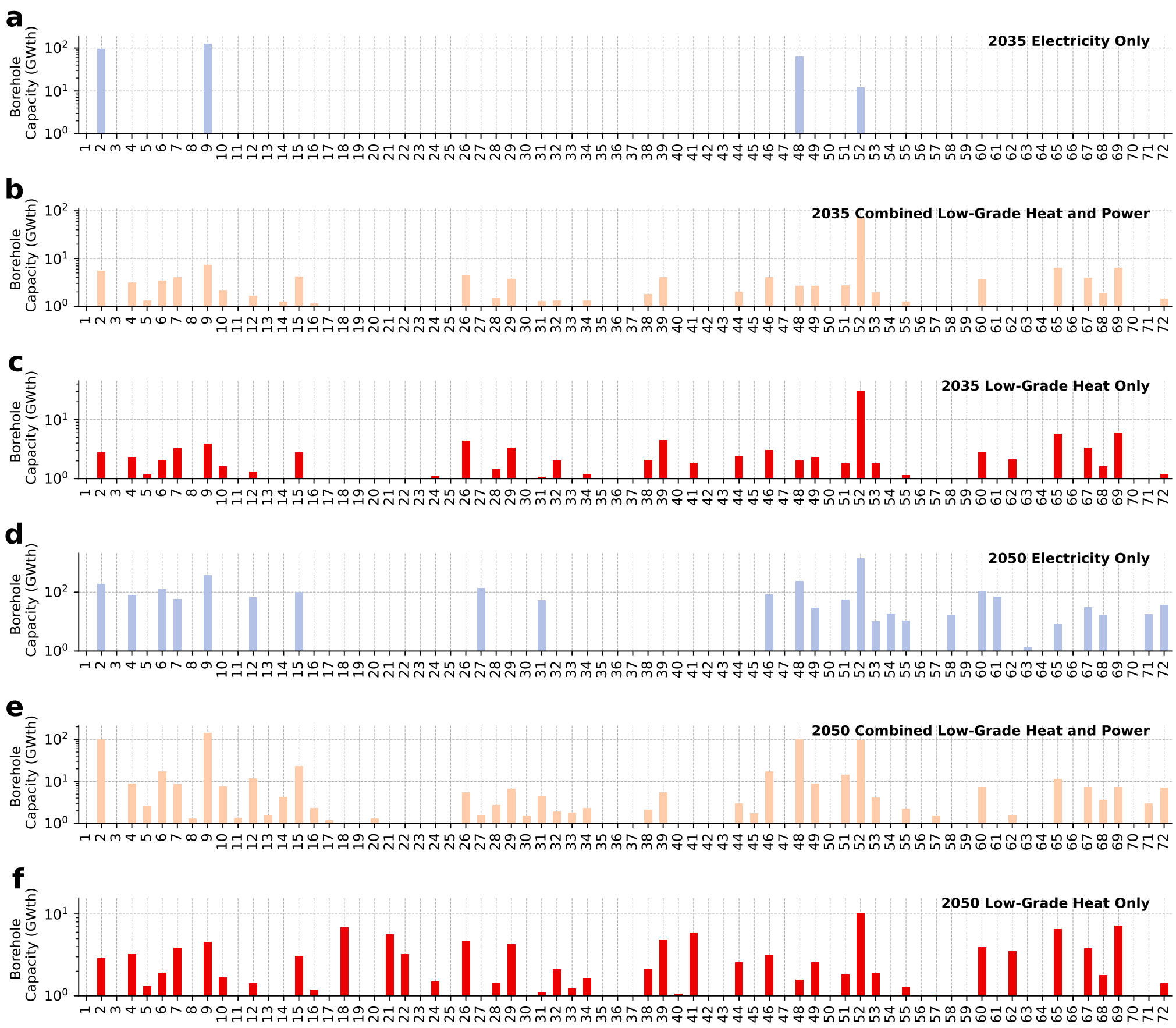

Supplementary Figure 8: Injection well borehole capacities for all individual network regions. Panels **a**–**c** show capacities in 2035 and **d**–**f** in 2050, for Enhanced Geothermal Systems generating electricity only (**a**, **d**), combined low-grade heat and power (**b**, **e**) and low-grade heat only (**c**, **f**). Labelling of regions as in Supplementary Figure 7.

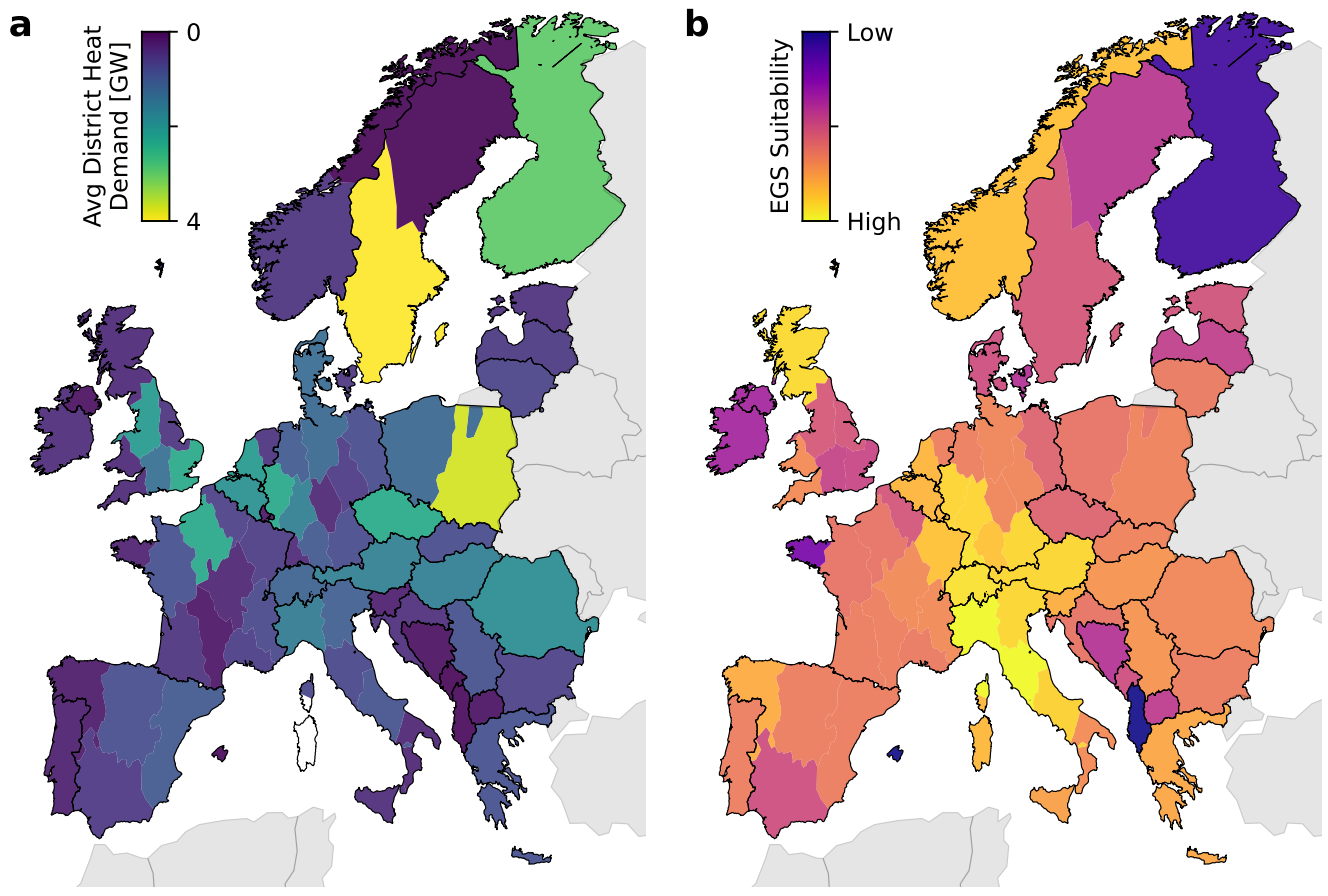

Supplementary Figure 9: **a** Average district heating demand per region assuming a district heating rollout progress of 30% (see Supplementary Figure 11) as assumed in the bulk of experiments and **b** relative Enhanced Geothermal Systems (EGS) suitability.

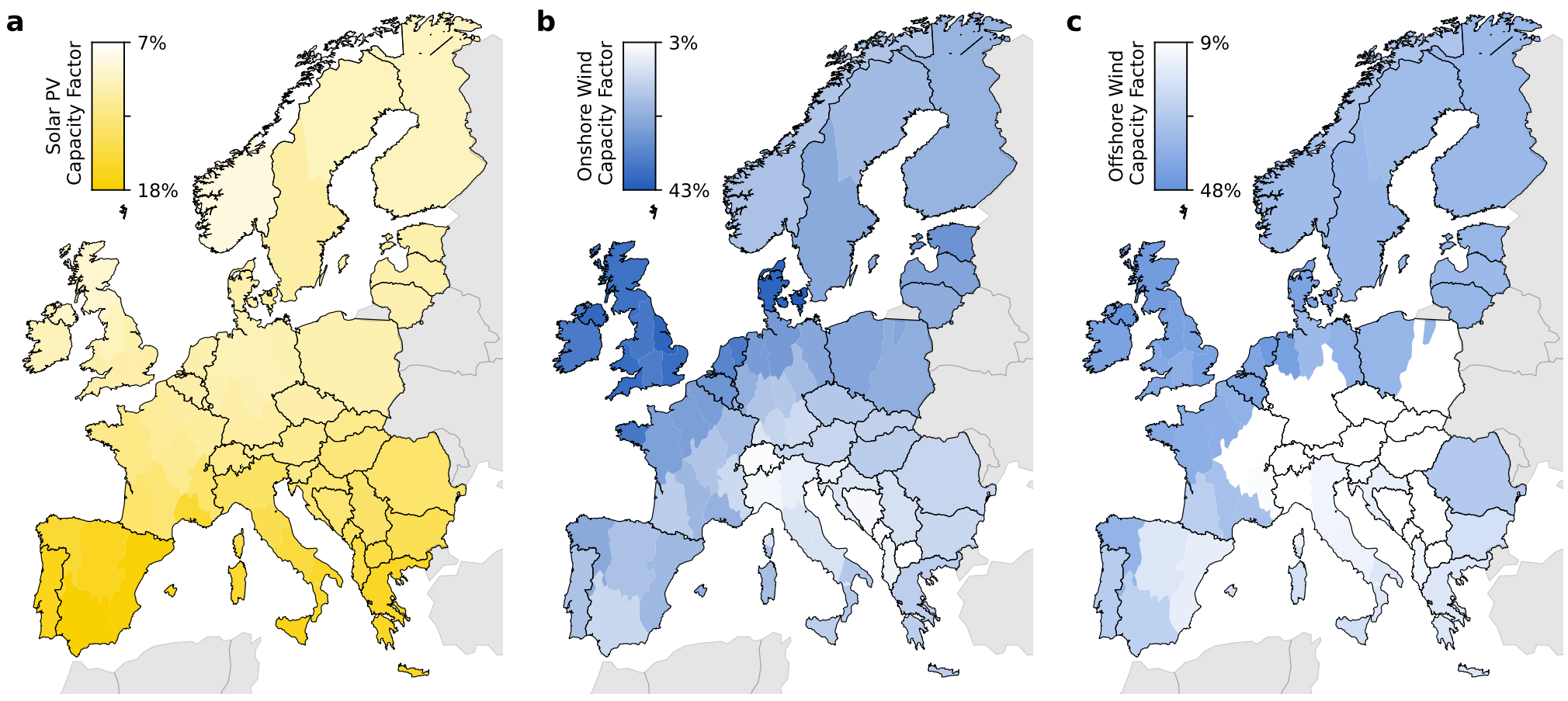

Supplementary Figure 10: Yearly average capacity factors of **a** solar photovoltaics (PV), **b** onshore wind and **c** offshore wind.

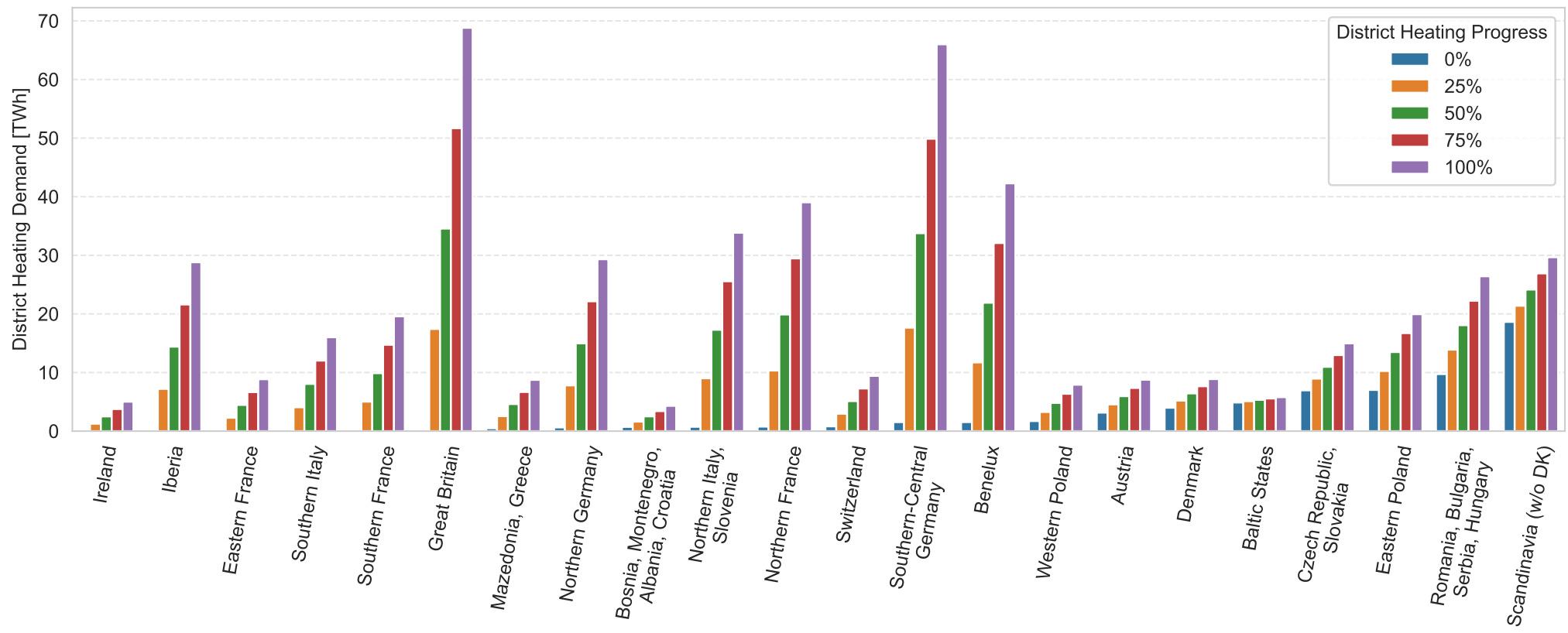

Supplementary Figure 11: Total district heating demand per aggregated regions for different assumptions about district heating rollout.

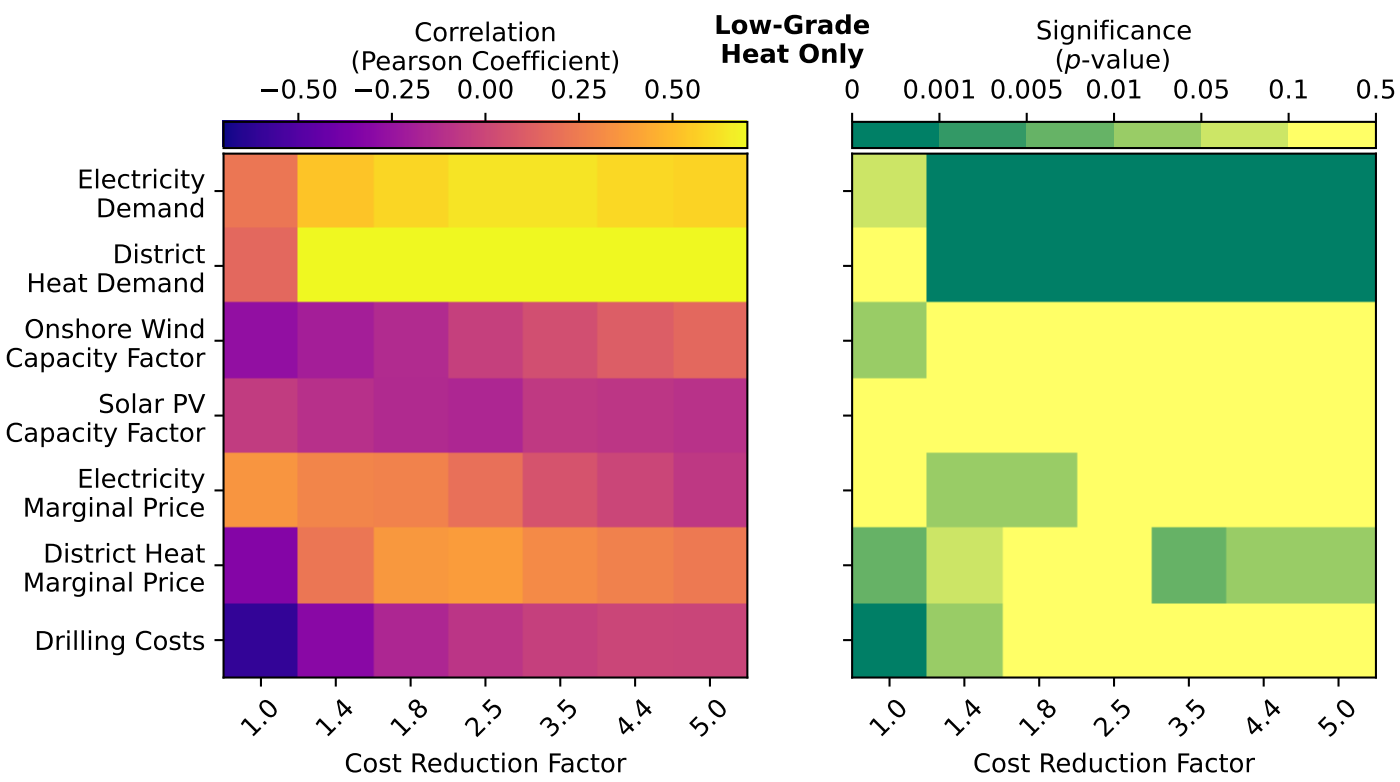

Supplementary Figure 12: Correlation between nodal features and buildout of low-grade heat Enhanced Geothermal Systems (EGS). PV: photovoltaics.

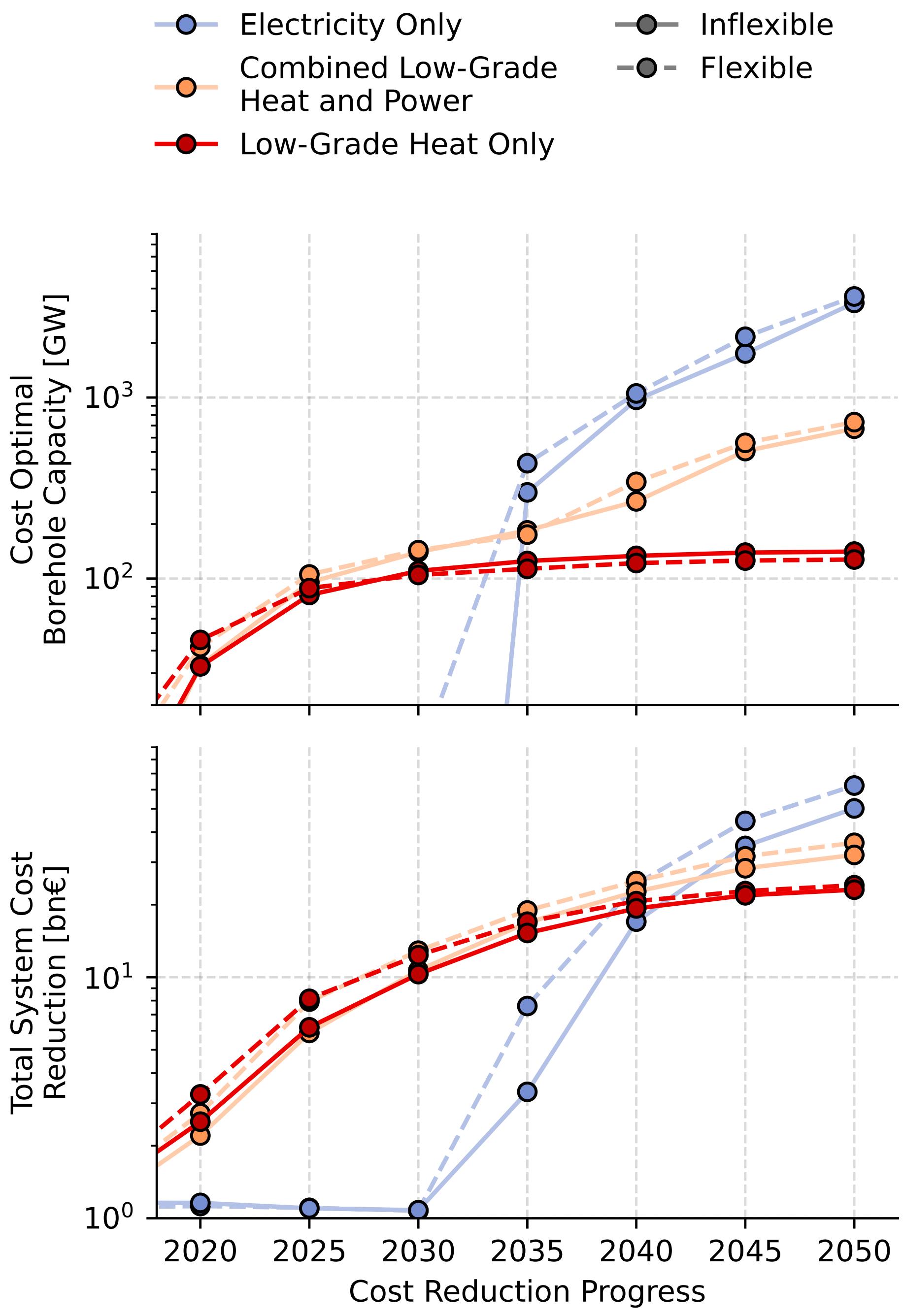

Supplementary Figure 13: Built borehole capacity and total system cost change when Enhanced Geothermal Systems (EGS) is operated flexibly.

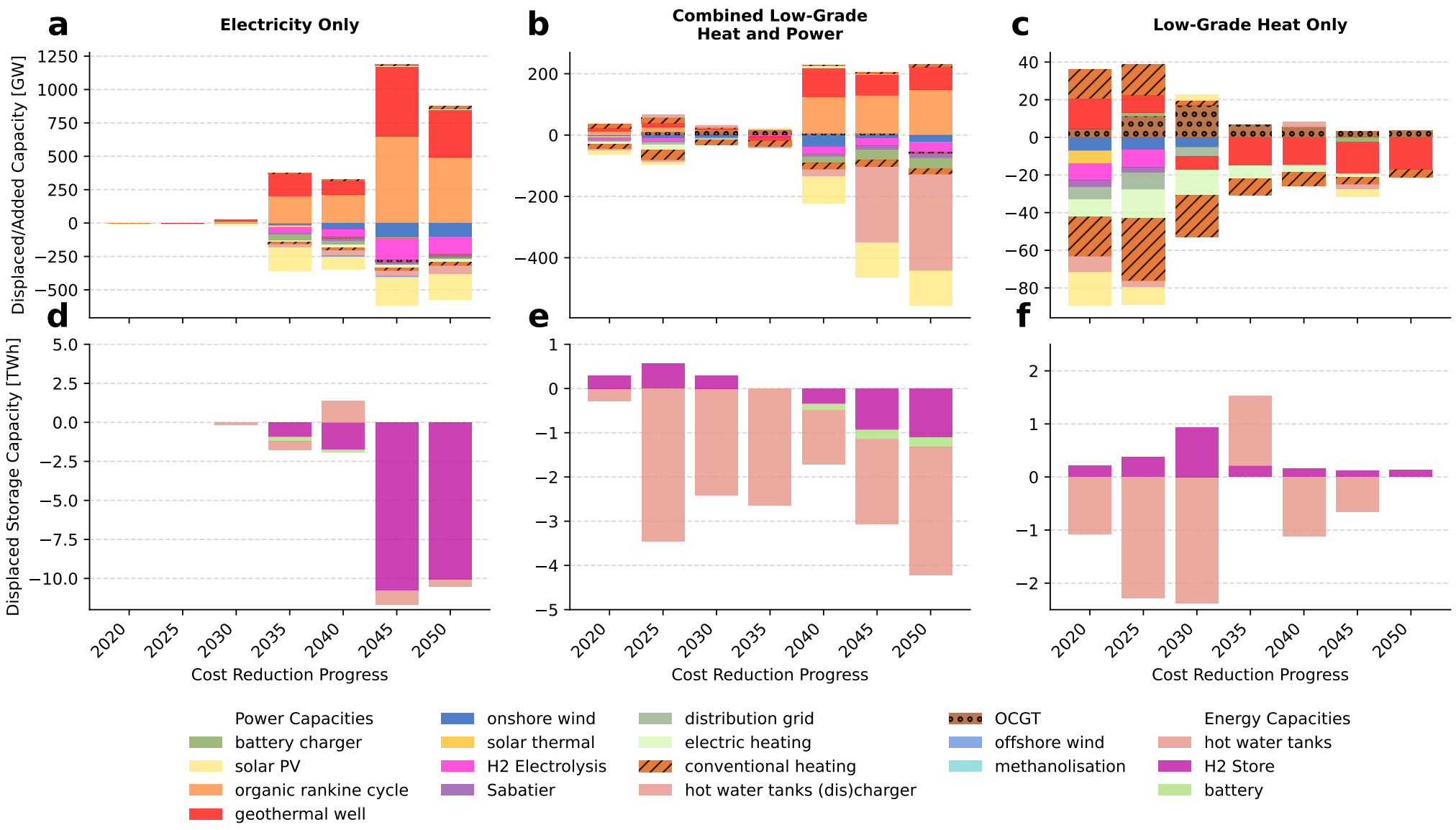

Supplementary Figure 14: Changes of installed capacities by the model when flexible operation is switched on for different Enhanced Geothermal Systems (EGS) cost reduction levels. Panels **a**–**c** show power capacities and **d**–**f** energy capacities. Positive values are added, negative ones are subtracted relative to a counterfactual where EGS is not available. PV: photovoltaics; OCGT: open-cycle gas turbine; H_2: hydrogen.

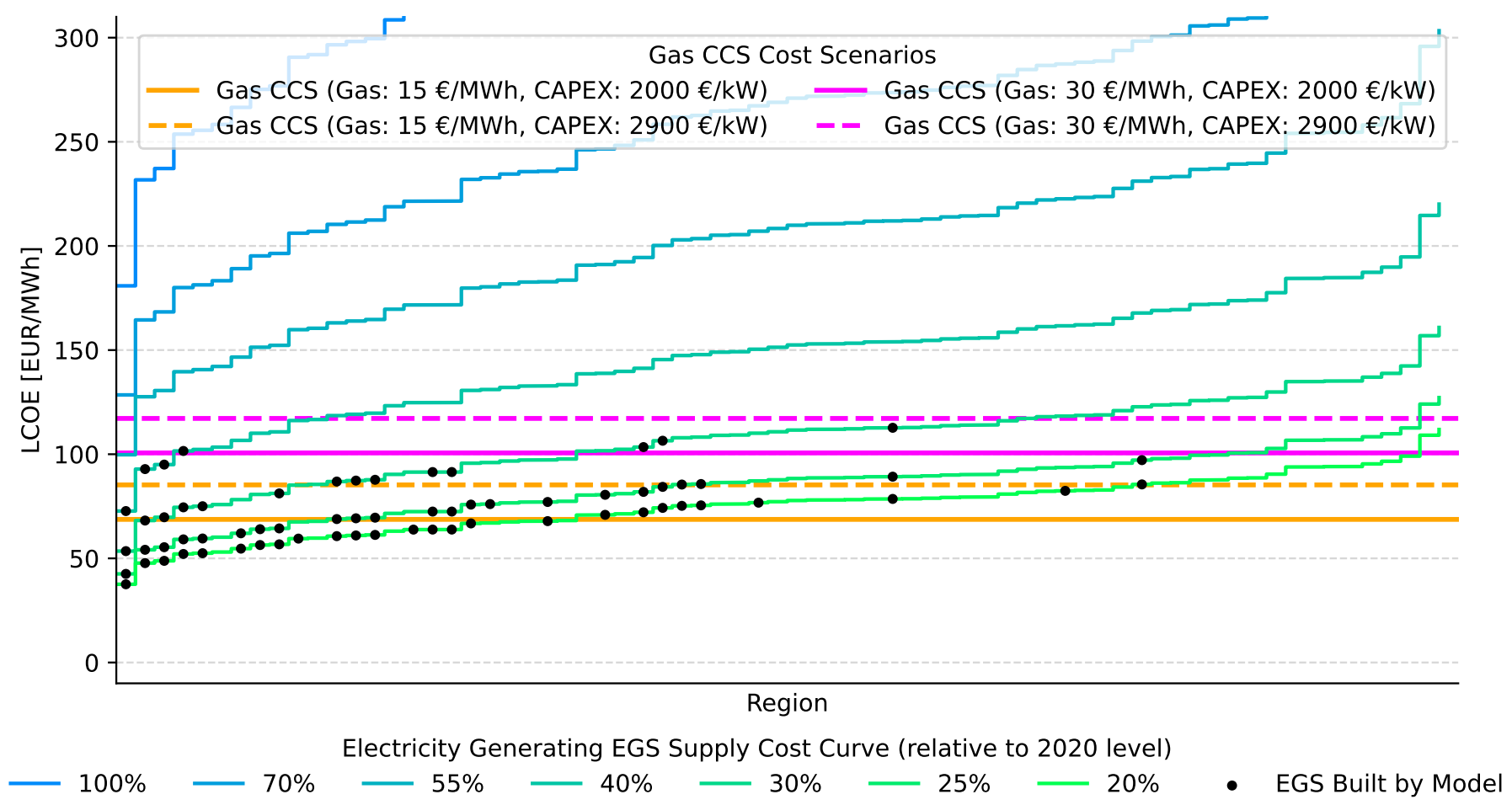

Supplementary Figure 15: Comparison of levelized cost of electricity (LCOE) values from Aghahosseini and Breyer [1] used in the model to different projections for gas generators with attached carbon capture and storage (CCS). The gas generators are assumed to operate at an efficiency of 47% [4] and a capacity factor of 50%. EGS: enhanced geothermal systems; CAPEX: capital expenditure.

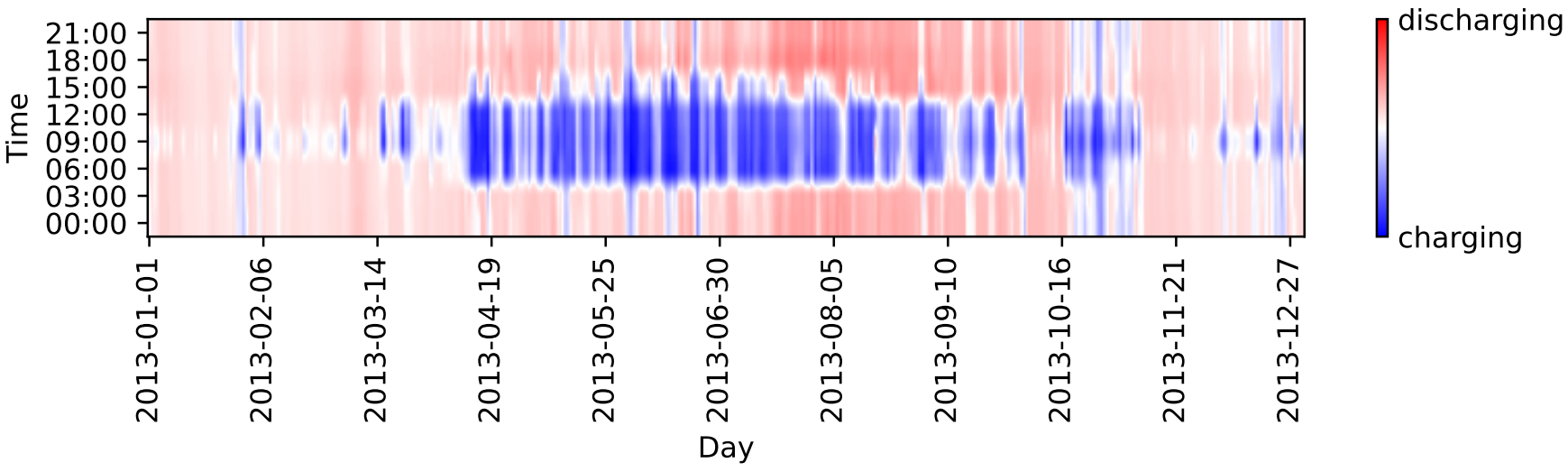

Supplementary Figure 16: Operational pattern of flexible Enhanced Geothermal Systems (EGS), visualised as an average across network regions for electricity-generating EGS at 30% of today's cost.

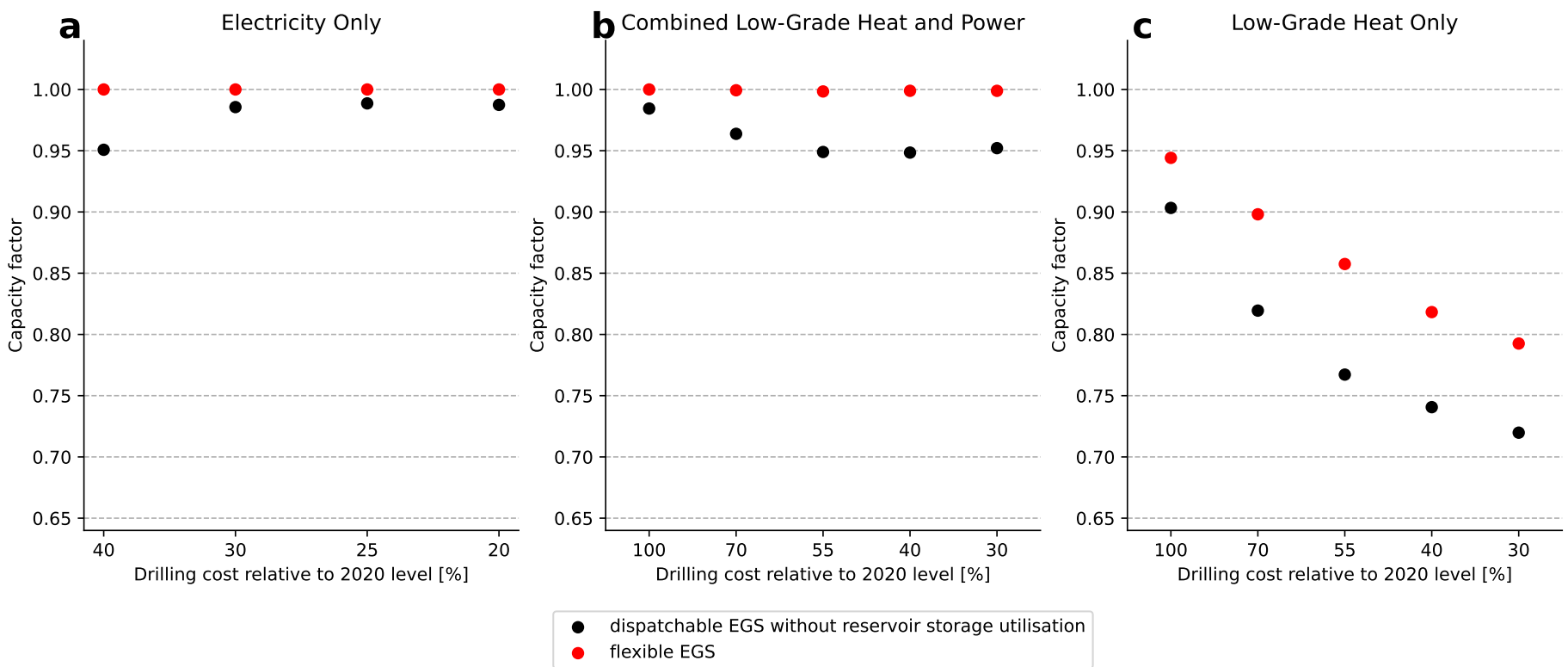

Supplementary Figure 17: Weighted average capacity factors for different Enhanced Geothermal Systems (EGS) modes and cost reductions, for EGS generating **a** *electricity only*, **b** *combined low-grade heat and power* and **c** *low-grade heat only*.

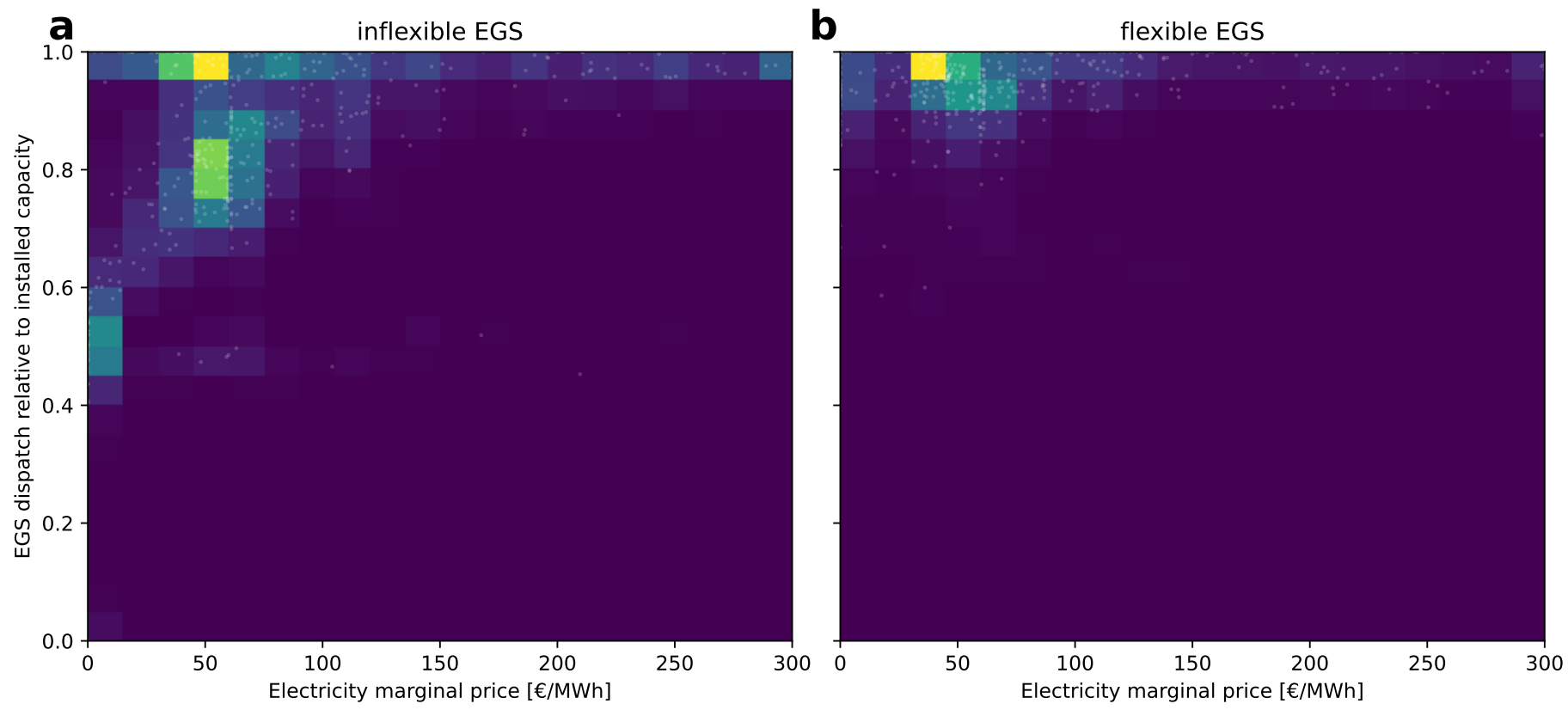

Supplementary Figure 18: Electricity-generating dispatch relative to installed capacity versus marginal price of electricity comparing **a** static and **b** flexible Enhanced Geothermal Systems (EGS) for 60% drilling cost reduction relative to 2020 cost. Each point counted in the contour represents one unique snapshot-bus pair in the model.

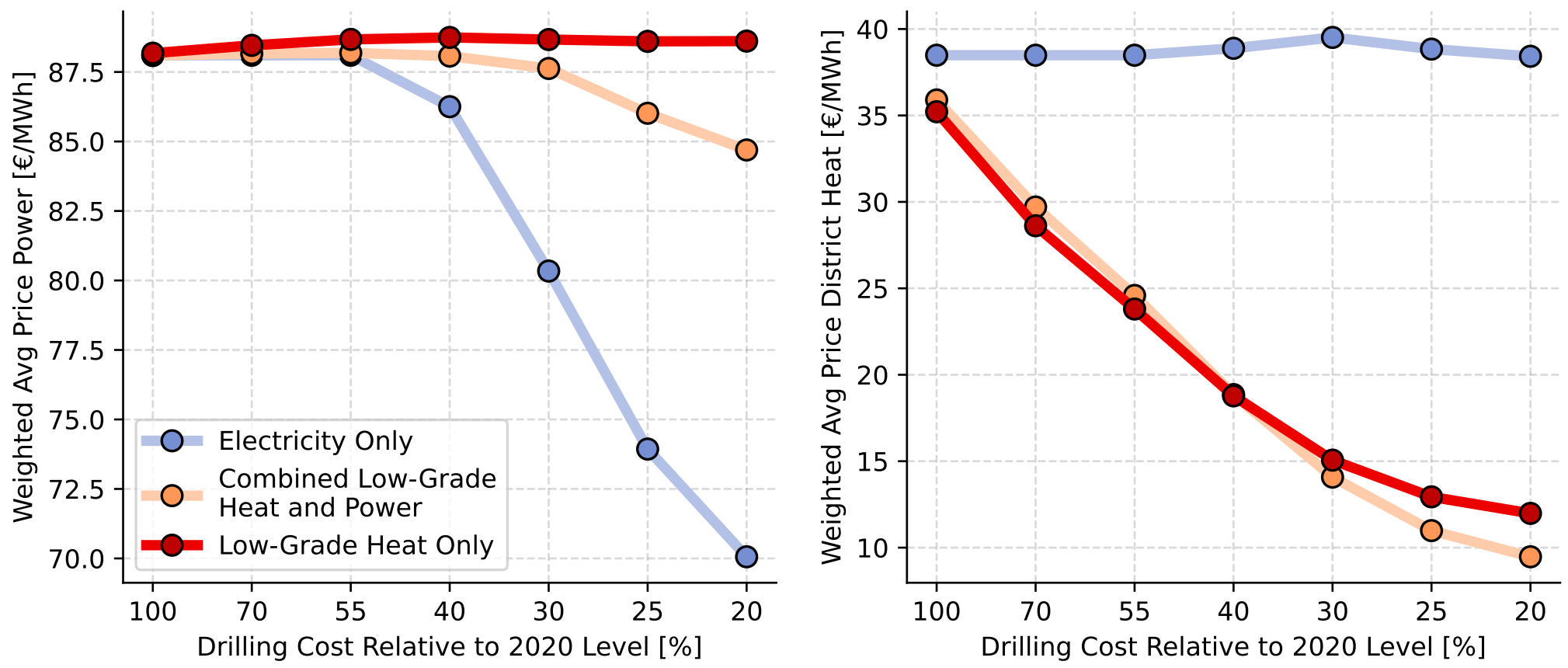

Supplementary Figure 19: Load-weighted average energy prices in electricity and district heating nodes for different levels of Enhanced Geothermal Systems (EGS) cost reductions.

Supplementary Table 1: Sensitivities tested relative to a base case of assumptions on the trajectory of the European energy system.

Sensitivity	Description	Tested Values
Flexible Operation	The application of backpressure to the production well induces an increase in temperature and pressure within the geothermal reservoir, leading to enhanced power generation upon resuming production[5]. This approach, known as flexible EGS, allows EGS to synergise with variable renewable energy generation, thereby overcoming the constraint of operating solely as a base-load generator. In this study, we examine the effects of EGS operation after a period of reduced generation.	We incorporate a storage capacity into the reservoir, which can be charged for up to 24 hours. The discharge from storage is constrained to 25% of the borehole's capacity. This represents an optimistic scenario, given the heightened risk of induced seismicity associated with such pressure increases[6].
Transmission Capacity	The extent of grid reinforcement in Europe beyond 2030 remains uncertain. However, future changes are likely to be minor compared to the existing network layout.	We consider the impact of a 12.5% and 25% expansion in additional volume, measured in gigawatt-kilometres (GWkm), on EGS capacity.
Waste Heat	Fuel synthesis processes, such as Fischer-Tropsch, are anticipated to provide a low-cost source of district heating. However, the extent of this supply remains uncertain. For heat (co-)generating EGS, we assess the impact of excluding Fischer-Tropsch as a low-cost heat source.	The utilisation of waste heat from fuel synthesis for low-temperature heat demands is deactivated.
Renewable Cost	The costs of wind, solar, and hydro generators have been updated.	A 20% increase or decrease in the base case capital expenditure.
District Heating	Although the expansion of district heating in Europe is included in many energy transition outlooks, its future rollout remains uncertain. PyPSA-Eur models varying levels of rollout using a parameter, where 0 represents the 2020 rollout level, and 1 corresponds to all regions meeting 60% of their urban residential heat demand through district heating. In the assumed base case with a rollout progress of 0.3, each region is scaled individually.	A zero-progression scenario assumes district heating shares remain at 2020 levels. Additionally, two scenarios are considered where district heating progresses to 60% and 100% of urban residential heat demand, respectively.

Supplementary Table 2: Lower confidence interval of Pearson R values for electricity generating Enhanced Geothermal Systems (EGS); CRF: Cost Reduction Factor; DH: District Heating; MC: Marginal Cost; MP: Marginal Price

CRF [%]	Elec. demand	DH demand	Wind Capacity	Solar PV Capacity	Elec. MC	DH MP	System Cost
100	-0.062	-0.140	-0.486	-0.352	0.278	-0.071	-0.946
70	-0.084	-0.117	-0.481	-0.361	0.334	-0.061	-0.901
55	-0.101	-0.064	-0.454	-0.330	0.286	-0.055	-0.792
40	-0.098	-0.042	-0.310	-0.267	0.016	-0.134	-0.568
30	-0.086	-0.051	-0.377	-0.258	0.081	-0.102	-0.646
25	-0.057	-0.018	-0.384	-0.271	0.164	-0.100	-0.685
20	-0.024	0.021	-0.376	-0.298	0.208	-0.099	-0.696

Supplementary Table 3: Upper confidence interval of Pearson *R* values for electricity-generating Enhanced Geothermal Systems (EGS); CRF: Cost Reduction Factor; DH: District Heating; MC: Marginal Cost; MP: Marginal Price

CRF [%]	Elec. demand	DH demand	Wind Capacity	Solar PV Capacity	Elec. MC	DH MP	System Cost
100	0.391	0.326	-0.055	0.107	0.641	0.383	-0.865
70	0.372	0.347	-0.049	0.097	0.677	0.392	-0.758
55	0.357	0.392	-0.014	0.132	0.647	0.398	-0.534
40	0.361	0.411	0.154	0.199	0.455	0.328	-0.160
30	0.371	0.403	0.078	0.208	0.505	0.356	-0.278
25	0.395	0.431	0.070	0.195	0.565	0.359	-0.341
20	0.423	0.462	0.079	0.167	0.596	0.360	-0.360

Supplementary Table 4: Lower confidence interval of Pearson *R* values for combined heat and power (CHP)-generating Enhanced Geothermal Systems (EGS); CRF: Cost Reduction Factor; DH: District Heating; MC: Marginal Cost; MP: Marginal Price

CRF [%]	Elec. demand	DH demand	Wind Capacity	Solar PV Capacity	Elec. MC	DH MP	System Cost
100	-0.035	-0.061	-0.420	-0.321	0.099	-0.467	-0.699
70	0.288	0.643	-0.372	-0.350	0.117	0.051	-0.481
55	0.355	0.804	-0.342	-0.384	0.134	0.150	-0.364
40	0.359	0.777	-0.337	-0.397	0.182	0.049	-0.420
30	0.066	0.295	-0.301	-0.360	0.153	-0.274	-0.514
25	-0.024	0.083	-0.332	-0.299	0.099	-0.406	-0.581
20	-0.023	0.050	-0.367	-0.271	0.117	-0.424	-0.615

Supplementary Table 5: Upper confidence interval of Pearson *R* values for combined heat and power (CHP)-generating Enhanced Geothermal Systems (EGS); CRF: Cost Reduction Factor; DH: District Heating; MC: Marginal Cost; MP: Marginal Price

CRF [%]	Elec. demand	DH demand	Wind Capacity	Solar PV Capacity	Elec. MC	DH MP	System Cost
100	0.414	0.395	0.028	0.142	0.519	-0.031	-0.366
70	0.648	0.846	0.084	0.109	0.532	0.482	-0.042
55	0.689	0.920	0.118	0.071	0.544	0.555	0.101
40	0.692	0.908	0.124	0.055	0.578	0.481	0.035
30	0.494	0.654	0.163	0.098	0.558	0.191	-0.085
25	0.423	0.510	0.130	0.165	0.519	0.044	-0.180
20	0.424	0.484	0.091	0.195	0.532	0.023	-0.229

Supplementary References

[1] A. Aghahosseini, C. Breyer, From hot rock to useful energy: A global estimate of enhanced geothermal systems potential, Applied Energy 279 (2020) 115769.

[2] J. Hörsch, F. Hofmann, D. Schlachtberger, T. Brown, PyPSA-Eur: An open optimisation model of the european transmission system, arXiv preprint (2018). URL: https://arxiv.org/abs/1806.01613. arXiv:1806.01613.

[3] F. Neumann, E. Zeyen, M. Victoria, T. Brown, The potential role of a hydrogen network in europe, Joule 7 (2023) 1793–1817.

[4] U.S. Energy Information Administration, Electricity, Coal, and Renewables Long-Term Modeling Team, Discussion on Capital Cost and Performance Characteristic Estimates for New Generating Technologies, Technical Report, U.S. Energy Information Administration, Washington, D.C., 2023. URL: https://www.eia.gov/, accessed 11 July 2025.

[5] W. Ricks, K. Voller, G. Galban, J. H. Norbeck, J. D. Jenkins, The role of flexible geothermal power in decarbonized electricity systems, Nature Energy 10 (2025) 28–40. doi:10.1038/s41560-023-01437-y.

[6] N. Deichmann, D. Giardini, Earthquakes induced by the stimulation of an enhanced geothermal system below basel (switzerland), Seismological Research Letters 80 (2009) 784–798.
