# Supplementary material for: Market Integration Pathways for Enhanced Geothermal Systems in Europe
Source: Nat Commun. 2026 Oct 16;17:11666. doi: 10.1038/s41467-026-77893-3 (PMC13760043; doi:10.1038/s41467-026-77893-3)
Supplement: Supplementary file 2 [file 41467_2026_77893_MOESM2_ESM.pdf]

nature portfolio

Peer Review File

Market Integration Pathways for Enhanced Geothermal Systems in Europe

Corresponding Author: Dr Lukas Franken

This file contains all reviewer reports in order by version, followed by all author rebuttals in order by version.

Version 0:

Reviewer comments:

Reviewer #1

(Remarks to the Author)
Overall, this manuscript shows valuable research about exploring how Enhanced Geothermal Systems could evolve in a carbon-neutral European energy system, offering valuable insights into cost thresholds, heat-sector integration, and the interplay between resource quality and market dynamics. However, several key areas require more extensive revision before publication. In particular, the paper should strengthen its methodological justifications (e.g., for modeling assumptions on district heating rollout, transmission constraints, and cost averaging), deepen scenario exploration to capture alternative firm capacity options and partial decarbonization, and temper some findings to acknowledge real-world barriers such as induced seismicity, regulatory hurdles, and financing constraints. Clarifying the pathways for large-scale EGS deployment (including potential policy measures, learning rate realism, and timelines for industrial ramp-up) would further enhance the paper's relevance and rigor. Addressing these points in detail will provide a more robust, transparent basis for the manuscript's conclusions.

1. The use of a high-resolution, multi-sector European energy system model (PyPSA-Eur) is appropriate. However, several key assumptions should be better justified. For example, the decision to prohibit transmission grid expansion is only briefly defended by a sensitivity showing small impact – the authors should provide more evidence or reasoning for this simplification, since transmission constraints can affect renewable vs. local EGS deployment. Similarly, the assumptions around district heating rollout (e.g. a 30% progress toward a 60% urban heat share) appear somewhat arbitrary; the manuscript should either cite supporting studies for these specific figures or explicitly test the impact of different heat network expansion rates (some of which is touched on in the sensitivity analysis, but it needs clearer justification in the main text). Additionally, the method of assigning EGS costs/potentials to regions by averaging 1°×1° resource data could mask important local variations – this cost-averaging approach might obscure low-cost "sweet spots" or overestimate poor-resource areas, so the authors should discuss how this spatial aggregation affects results and consider a finer resolution or at least acknowledge the uncertainty it introduces.
2. The assumed drilling cost declines (up to ~60–80% reduction from today) and the implied learning rates deserve a more critical examination. The authors adopt cost reduction factors from prior work and later infer a ~20–25% learning rate needed for EGS – this is notably aggressive (comparable to solar PV learning rates) for a technology like drilling that historically sees slower gains. It is recommended the authors justify the realism of these cost trajectories: Are there empirical precedents or specific innovations (e.g. advanced drilling techniques, multi-well pads, oil/gas industry spillovers) that support such steep cost declines? It would strengthen the paper to acknowledge potential deployment constraints on learning – for instance, if EGS deployment is initially very limited due to high costs, substantial exogenous support or global deployment (beyond Europe) might be required to achieve those cost reductions. In its current form, the modeling effectively treats cost drops as exogenous scenario inputs; a discussion of investment pacing (how quickly capacity can scale to drive learning) and whether policy-driven initial deployments or international R&D efforts are assumed would add important context. In short, the cost and learning assumptions seem optimistic – the authors should add caveats or sensitivity cases (e.g. slower learning, or a cap on annual build rate) to ensure conclusions aren't overly reliant on idealized cost reduction pathways.
3. The simplifications in how EGS technology is modeled should be more thoroughly justified and, if possible, refined. Notably, the model uniformly sets the geothermal production temperature (150 °C) and uses a single efficiency/cost parameterization for power conversion – while this streamlines the analysis, it ignores site-by-site differences in reservoir temperature and conversion efficiency (which in reality range ~10–20% for electricity). This uniform approach risks biasing results (e.g. underestimating the benefit of a few high-temperature sites or overestimating performance in marginal areas). The authors themselves note in the limitations that geological performance uncertainties and resource variability are

neglected; I suggest explicitly acknowledging in the main text that EGS output and costs likely vary by location and that results are an idealized case. In terms of operational flexibility, the study commendably explores a “flexible EGS” scenario (injecting fluid to store thermal energy and boost output later) – however, this is presented as an optimistic case given seismicity risks. It would be useful if the authors clarify how EGS is operated in the base scenarios: is it assumed to run as baseload at full output, or can it be dispatchable (e.g. turndown when electricity prices are low)? If not already detailed, the manuscript should describe any dispatch constraints or flexibility assumptions for EGS (beyond the special 24h storage case). Finally, the co-generation modeling appears to allow EGS plants to supply both heat and power, which is a strength of the study – the authors might want to ensure they explain how the model optimizes the split between heat vs. electricity output. For instance, is the ratio fixed or can the plant allocate thermal output dynamically based on demand? Overall, while the technical representation is simplified, it is mostly reasonable for a system model – but the manuscript should more clearly state these simplifications (temperature, efficiency, dispatch limits) and perhaps discuss the implications (e.g. slightly overstated EGS potential if real-world operational downtimes or performance variations were considered).
4. The scenario range could be expanded to better capture key uncertainties and alternative futures. The authors focus on varying drilling costs (which is central) and perform sensitivities on district heating expansion, renewable costs, grid, etc., but it would strengthen the study to test or discuss additional scenarios. For example, one critical uncertainty is district heating rollout speed: the manuscript’s base assumption is moderate growth, but a scenario with delayed or minimal heat network expansion (beyond today’s levels) is important to consider – the authors do include a “zero-progress” case, but this result should be highlighted in the discussion (e.g. noting that EGS uptake falls significantly if heat networks don’t expand). Conversely, exploring an aggressive district heating scenario (or highlighting the one they did with 60%–100% urban coverage) helps bound the potential. Another missing dimension is alternative firm capacity solutions: the current model appears to exclude options like new nuclear or fossil CCS, meaning EGS (plus storage and hydrogen) fills the firm supply gap. The paper would be more convincing if it at least acknowledged or tested a case where other firm low-carbon technologies are available (e.g. a scenario with nuclear or gas+CCS in the mix) – this would show how EGS competes in a broader technology landscape and ensure the results aren’t an artifact of a limited option set. Additionally, the manuscript assumes a fully net-zero 2050 system; discussing a partial decarbonization pathway (where climate targets are weaker or reached later) could be valuable, since EGS economics might differ if carbon prices are lower or if gas heating persists longer. In summary, the scenario exploration would be improved by covering a wider uncertainty envelope – for instance, varying the policy context (strong climate policy vs. moderate), heat demand electrification vs. district heating emphasis, and even availability of other firm renewables (like bioenergy or long-duration storage). These additions would give readers more confidence that the conclusions about EGS’s role hold under different plausible futures, not just the specific setup chosen.
5. Real-world deployment of EGS will face geographical and societal constraints that are not fully captured in the model’s least-cost optimization. The manuscript should more directly acknowledge factors like regulatory barriers, permitting hurdles, and social acceptance issues that could constrain where EGS projects can actually be built. For instance, certain regions with high geothermal potential (or even existing pilot projects) have experienced public opposition or moratoria due to induced seismicity concerns (e.g. Germany’s restrictions after seismic events). Are the identified “high-opportunity” regions for EGS in the results actually likely to allow large-scale deployment? If not, that could significantly reduce practical potential. The authors are encouraged to integrate these external constraints qualitatively (at minimum) or even quantitatively. One suggestion is to discuss a scenario or sensitivity where EGS deployment is restricted in some densely populated or high-seismic-risk areas – how would the system cope (perhaps via more of other firm sources)? Even if not modeled explicitly, the paper should emphasize that permitting and public acceptance could slow the rollout: e.g. lengthy project lead times, the need for community engagement, and possible exclusion of certain regions. Additionally, regulatory frameworks (like drilling regulations, subsurface rights, or environmental impact requirements) vary by country and could delay projects beyond what an optimized model assumes. By addressing these geographical and societal constraints, the authors will provide a more nuanced outlook – highlighting that the theoretical potential from the model is an upper bound contingent on favorable regulatory and public environments. Integrating such insights (perhaps in the discussion or a dedicated subsection) will make the analysis more realistic and policy-relevant.
6. The manuscript would benefit from a more cautious interpretation of results to ensure findings are not overstated. In several places, the language could be tempered to clarify that outcomes are conditional on assumptions (e.g. cost reductions, policy support, etc.). For instance, when discussing EGS becoming competitive in electricity markets with ~60% cost reduction, it should be clear this is an “if/then” result rather than a prediction – i.e., “if drilling costs drop by X, then EGS could expand by an order of magnitude,” rather than implying it will definitely happen. It is suggested adding such conditional phrasing and explicitly noting that these scenarios represent optimistic, best-case conditions for EGS (especially since a net-zero system inherently favors it). Additionally, some results might be currently framed in a way that implies greater certainty than warranted. For example, the paper quantifies system cost savings and optimal capacities to high precision; given the uncertainties, the authors should emphasize these numbers are illustrative estimates under the model’s conditions. It would also help to highlight any important caveats in the main text (not just in the limitations section). For example, investor behavior and financing realities are not captured in the model’s perfect-foresight least-cost approach – so the real-world investment in EGS might lag even if it’s “optimal” on paper. A clear statement that market-optimal deployment does not guarantee investor willingness or immediate feasibility would prevent overinterpreting the capacity results. By more explicitly framing conclusions with their caveats (cost uncertainty, model simplifications, policy dependencies), the authors can avoid readers or policymakers drawing overly deterministic conclusions from what are scenario-based insights.
7. The paper currently underplays the practical hurdles associated with large-scale EGS deployment, and it should dedicate more attention to these aspects. Enhanced Geothermal Systems often face public and regulatory challenges – notably, permitting delays, stringent regulation on induced seismicity, and local public opposition due to safety or environmental concerns. The authors briefly mention that expanding other renewables can face public opposition, but they should equally note that EGS is not immune to this: past EGS projects (e.g. in Basel or Pohang) have been halted by induced earthquakes, leading to public wariness. Does the analysis assume that all proposed EGS capacity can be built on schedule? If so, that may be optimistic given the likelihood of lengthy permitting processes (drilling approvals, environmental impact

assessments) and possible moratoria if seismic events occur. Including a discussion on how policy and public engagement would be needed to address these risks (for example, improved micro-seismic monitoring, compensation schemes, or community benefit agreements) would balance the technology optimism with real-world prudence. In summary, the manuscript should acknowledge that even if EGS becomes economically attractive, non-economic barriers could slow its adoption – and these need proactive management. By addressing this, the authors ensure the reader understands the difference between economic potential and realizable capacity on the ground.

8. The scenarios suggest a very rapid scale-up of EGS capacity once it becomes cost-competitive (potentially hundreds of GW_th in a couple of decades), and the feasibility of this pace should be discussed. Rolling out EGS at the modeled scale would require an enormous ramp-up in supply chains, skilled workforce, and drilling infrastructure. Currently, only a handful of EGS projects exist in Europe; reaching even the "first phase" ~20–30 GW_th identified would mean drilling hundreds of deep wells and building out many combined heat/power facilities in a relatively short period. It is recommended that the authors explicitly comment on whether this expansion rate is realistic. Are there enough drilling rigs and crews available? How quickly can the industry grow while maintaining safety and quality? Potential bottlenecks like the availability of drilling equipment, rigs, cementing crews, and even the manufacturing of power turbines or heat exchangers for EGS plants could significantly slow deployment in practice. It would strengthen the manuscript to mention that achieving the modeled trajectory would likely require coordinated efforts to expand the industrial capacity (perhaps analogous to how the shale gas/fracking boom required scaling up rig counts). If the authors have any insights from industry reports or historical analogues (e.g. the growth of the oil & gas drilling sector or wind industry ramp-up), they could briefly include those; if not, a general caution that "the model does not account for maximum build rates or supply-chain limitations, which could delay reaching these capacity levels" would suffice. Acknowledging this limitation ensures readers appreciate that the timeline of EGS adoption might be much more gradual than the model's equilibrium solution, absent extraordinary mobilization.

9. The manuscript's results have clear policy implications, but the discussion could do more to translate them into actionable recommendations. Since the study indicates district heating expansion and cost reductions are key to EGS viability, policymakers should be informed about which levers to pull. For instance, carbon pricing is implicitly crucial (the model assumes carbon-neutrality); the authors could emphasize that a strong carbon price or equivalent regulatory measure is needed to make EGS (and other low-carbon heat options) competitive against fossil heat. Likewise, enabling EGS's contribution to heat implies supportive district heating policies – such as funding for heat network infrastructure, mandates or incentives for connecting buildings to communal heating, and urban planning that accommodates geothermal heat sources. The paper should also highlight geothermal-specific risk mitigation policies that exist or could be expanded: for example, government-supported insurance schemes for drilling (to de-risk the resource uncertainty), R&D programs for advanced drilling tech, or "green heat" tariffs. By detailing such policies, the authors can guide readers on how to catalyze the first phase of EGS deployment. Currently, the text hints at coordination being crucial but stops short of specifics; it would be valuable to articulate that coordination could take the form of an EU-wide geothermal innovation program or shared knowledge networks to achieve the learning rates identified. In summary, making the market and policy relevance more explicit – e.g., "our findings suggest that without interventions like X or Y, the modeled outcomes may not materialize" – will make the paper more useful to decision-makers and strengthen its impact.

10. The economic barrier of upfront investment for early EGS projects is high, and the manuscript should address how those might be overcome. At current costs, even though the model finds some EGS is cost-competitive for heat, private investors might be reluctant due to the high capital risk and unproven nature of EGS in many regions. It is recommended that the authors discuss or cite mechanisms to finance initial projects – for example, public funding or guarantees for first-of-a-kind (FOAK) EGS plants, dedicated EU or national grants/loans, or public-private partnerships to share risk. If the model's results rely on cost reductions via learning, there is an implicit need for upfront "loss-leading" investments; the paper should make this explicit by suggesting that early deployment will likely require subsidies or risk underwriting (since waiting for costs to drop on their own may never happen without initial volume). The authors could strengthen the discussion by referencing how other technologies (offshore wind, solar, etc.) saw rapid cost declines: nearly all had significant government support in early phases (feed-in tariffs, tax credits, etc.), which may also be necessary for EGS. Additionally, the concept of an "investment trap" could be mentioned: EGS won't get cheaper until it's deployed at scale, but it won't deploy at scale until it gets cheaper – breaking this deadlock likely requires policy intervention. By including these points, the manuscript will be able to better address the financeability of EGS, reassuring readers that the authors have considered the real-world economics and not just the model optimality. In practical terms, suggestions such as geothermal risk insurance funds, drilling cost-sharing programs, or targeted capital grants would show what enabling measures align with the study's findings.

11. The integration of EGS into energy markets (both electricity and heat) may necessitate changes in market design, and the paper should touch on this to round out the policy discussion. For the electricity market, if EGS eventually provides a significant firm capacity (especially in the high cost-reduction scenario), current energy-only markets might undervalue its contribution to reliability. The authors should consider noting that mechanisms like capacity payments or adequacy markets could reward EGS's ability to deliver steady power, ensuring it's financially viable to build even if energy prices are depressed by abundant renewables at times. Similarly, valuing ancillary services (frequency support, grid stability) could improve the economics of EGS power plants if they run flexibly – did the model account for any ancillary service provision? If not, it's worth mentioning that in reality EGS could have additional value streams. On the heat market side, the manuscript already identifies district heating as crucial; the authors might elaborate that heat markets often lack transparent pricing or open competition. They could recommend market reforms or regulations for heat: for example, ensuring that district heating networks allow third-party geothermal heat injection on fair terms, or implementing heat purchase agreements (analogous to PPAs in power) to guarantee offtake for geothermal heat. Moreover, if EGS plants are to optimize co-generation, the coordination between power and heat markets must be considered (e.g. pricing such that operators have incentive to produce the most needed form of energy at a given time). Including a brief commentary that market rules need to evolve – for instance, integrating heating into energy planning, or providing long-term contracts for firm low-carbon capacity – would demonstrate that the authors have thought through the practical integration of EGS beyond just technical feasibility. Such discussion can be kept concise but will significantly enhance the paper's relevance to energy planners and regulators.

12. The manuscript engages with existing studies [2–4,15,16, etc.], but a more thorough differentiation could strengthen its

positioning. It would be beneficial for the authors to explicitly contrast their findings with those of earlier works. For instance, if Longa et al. [4] found ~25 GW_e of EGS in an IAM and this study finds a comparable magnitude (or perhaps more when including heat), the authors should discuss why – e.g., "Longa et al. assumed optimistic cost but no spatial detail, whereas our results show a similar order-of-magnitude potential concentrated in specific regions under certain conditions." Also, the paper might consider citing additional relevant literature on geothermal integration or firm renewables for completeness. There have been recent energy outlooks (e.g., IEA or IRENA scenarios, IPCC reports) which typically project limited geothermal uptake; referencing these and then explaining why this study might diverge (due to inclusion of heat co-generation or different cost assumptions) would contextualize the work. If any studies on district heating and geothermal (perhaps from a European Commission report or national plans) exist, citing them could back the importance of heat integration. The authors mention EGS co-producing lithium [16] – this is a good example of broadening context. They could also mention any known demonstration projects or national targets for EGS to show awareness of the landscape. Ensuring all statements like "widely seen as an ingredient of cost-efficient heat supply" have appropriate references will guard against unsupported claims. Overall, the literature review is on the right track; a few more references or clearer comparisons – especially highlighting what past models missed (e.g., high temporal resolution for flexibility, explicit learning rate analysis, or pan-European coordination issues) – will underline the manuscript's originality and thoroughness.
13. the manuscript's framing should stress the wider significance of the findings beyond the immediate European case. The authors can elevate the impact by drawing out generalizable lessons: for example, the idea that a niche market (heat) can serve as a springboard for a new technology to later become mainstream in electricity is a concept that could apply to other regions or technologies. They should make it clear that while the study is set in Europe, the insights on EGS learning and integration could inform geothermal strategy in other parts of the world (e.g. how combining heat & power applications might make EGS viable in Asia or North America's decarbonization efforts). In the introduction or conclusion, a sentence or two linking the results to global decarbonization (perhaps noting that deep geothermal could provide a similar firm renewable option in regions with suitable geology, or that the challenges identified – cost reduction, heat infrastructure, public acceptance – are common everywhere) would broaden the paper's appeal. Additionally, the framing could be sharpened by emphasizing the relevance to current energy policy debates: for instance, many countries are now seeking firm, dispatchable clean energy to complement wind/solar – this study provides timely insight into where and when EGS might fill that role. The authors might also consider a slightly more accessible summary of the core message in the conclusion: e.g., "Our analysis indicates that enhanced geothermal could evolve from a small heat-providing niche today into a major player in future power systems, but only if substantial cost reductions are achieved through coordinated deployment and supportive policy." Such a statement distills the significance for a broad scientific readership. Finally, to maximize visibility, the authors should ensure the title and abstract clearly reflect the most striking outcomes (currently they do convey the key points quite well). Emphasizing keywords like "net-zero energy system," "firm renewable energy," and "techno-economic learning" in the framing can also help position the paper at the forefront of energy systems research.

(Remarks on code availability)
I just clicked the link, but I didn't review the codes line by line, also didn't try to install and run the code.

Reviewer #2

(Remarks to the Author)
The authors delivered a study about the development of EGS (Enhanced Geothermal Systems) with respect to drilling cost. It is a good approach to see the task of EGS to provide electricity, combines heat and power, and heating. Therefore the influence of drilling costs is different for the different energy provision options. It is also good to consider the whole spectrum of energy sources and/or storage options.
Nevertheless, I missed the recent technical developments of EGS, e.g. in Utah or Blue Mountain (see Geothermal Power Generation (Second Edition), Developments and Innovation, Woodhead Publishing Series in Energy 2025, Pages 451-473 Enhanced Geothermal Systems: Review and Status of Research and Development) (Xing et al., 2023), (Norbeck et al., 2023). EGS is not equal EGS and the way of multi stage stimulation has a significant influence on the energy output and therefore on LCOE. I recommend to include such technological aspects in a revised manuscript.

(Remarks on code availability)

Reviewer #3

(Remarks to the Author)
This paper analyzes future market potentials for enhanced geothermal systems (EGS) in Europe. The analyzed scenarios are different levels of drilling costs reductions within a net-zero multi-sector energy system. In addition, 3 different EGS operation modes were considered: electricity only, heat only and combined heat and electricity generation. While the paper provides some interesting results and sound methodology, there are some important points that need to be reviewed.

1) The paper focuses primarily on EGS costs as the main driving factor for market opportunities. However, other factors might play an important role as well for EGS adoption, for example, social acceptance and - especially - the induced seismicity. The seismicity factor should be addressed and discussed in the paper.
2) Methodology: all model runs use only single mode of EGS operation. However, especially in the context of spatial distribution across Europe, it would also make sense to consider different EGS modes at the same time and let the model 'decide' where to install each EGS type (mode).
3) Methodology: the paper does not clearly state the modelling year (if there is one) for the net-zero Europe energy system.

This year, e.g. 2050, will influence vast majority of model parameters, such as projected energy demands, costs of other technologies, including renewables, etc. It is important to clarify this more.
4) Methodology: related to 3) - cost reductions of other technologies are not addressed sufficiently. Only in the sensitivity analysis, there is analysis of cost reductions of renewable by +/- 20%. Since this has a major impact on the overall results, more analysis and investigation regarding cost reductions is needed. For example, what are the potential cost reductions (projections) for other technologies, especially renewables, and how do the compare to EGS case? What is the historical development here?
5) In the context of 4) and multiple other assumptions (with uncertainties) in the model - such as assumptions on pg. 11 about EGS capital expenditure, the cost of heat integration, etc. - it makes more sense to provide the main results as ranges (distributions) rather than single values. Using single value results, such as 60% cost reduction as tipping point, can be misleading in this context.
6) In addition to this, there are other major limitations explained in section "Limitations", which further make uncertain the overall results - and limit the statements. This is especially the case for neglecting the temporal nature of energy transition. While these are discussed in the section, they were not reflected anywhere before in the paper - this needs to be further addressed.
7) Model: Looking at Fig A.10 or A.11, it can be seen which technologies were modeled in the framework. Some technologies, that might be crucial, are not represented, for example: shallow geothermal systems (open and closed-loop) as well as 'classical' deep geothermal systems. Both of these technologies are, for example, important in countries such as Germany, especially with the planned new capacities. Are the technologies considered in the model? If not, why?

Other (smaller) points:
8) Fig A.14 shows Albania in the model as region 1, however this region is 'empty' in Fig A.13
9) Title of Fig A.9. - please pay attention to grammar
10) Pg. 11: it is mention 'we simulate ... ORC' and 'simulation of borehole operation' - this is misleading. More proper would be 'we model'.
11) Pg. 10: 'Our default assumption is a progress of 30% ...' - based on what?
12) Pg. 4: OCGT - it was not explained before what this is
13) Pg. 4: '(co-)gen-' and then it was skipped to new row
14) Pg. 3: word 'which' goes outside of the 'text frame' - this happens throughout the paper and needs to be fixed

(Remarks on code availability)
The code is freely accessible and provides a README file with clear instructions for the usage. However, I have not try to install and run the code.

Version 1:

Reviewer comments:

Reviewer #1

(Remarks to the Author)
The authors have addressed many of the earlier comments in detail, and several additions (e.g., flexible EGS operation, spatial adoption maps, drilling cost sensitivity) significantly improve the manuscript. However, some technical aspects require further clarification or strengthening.

1. The manuscript now links drilling cost reductions to learning rates (~20–25%) but still treats them somewhat simplistically. It remains unclear how robust these estimates are across different global deployment scenarios. A comparison to other energy technologies' historical learning curves (wind, solar, shale gas drilling) would strengthen the case.

2. The sensitivity test of transmission capacity (+12.5% / +25%) is acknowledged, but the justification for using only modest increments is thin. Given that long-distance transport of EGS electricity is critical for Central European concentration, more rigorous treatment (or discussion of modeling limits) is needed.

3. Figures of system cost reductions show system savings <1%–6%. While acknowledged, the authors should clarify whether these differences are within model uncertainty bands. Otherwise, the significance of such small changes is questionable.

4. The manuscript briefly notes China/Russia industrial heat opportunities, but the discussion remains superficial. More depth on how European learning could transfer globally would improve the framing.

5. Although mitigation strategies of induced seismicity are cited, the risks of field implementation remain understated. The authors should balance optimism with clear acknowledgment of uncertainties.

6. regarding the PyPSA model, the paper does not show any benchmarking of the modified EGS module against existing field data or alternative models. Even a qualitative comparison would improve confidence.

7. The flexible-operation scenario is mentioned, but how the temporal resolution (hourly/daily) of the energy system model captures reservoir storage dynamics remains vague.

8. The displacement of water tanks by EGS at high penetration is intriguing but needs more mechanistic explanation. Why does the model prefer to eliminate short-term storage entirely instead of co-optimizing?

9. The back-of-the-envelope calculation on drilling rigs is helpful but simplistic. A more nuanced treatment (e.g., learning in drilling speed, supply-chain bottlenecks) would strengthen the argument.

10. regarding the economic metrics, only system cost reductions are reported. Metrics like LCOE distributions, payback time, or capacity factors would make the results more actionable for policymakers.

11. The manuscript mentions EU directives, but lacks discussion of how national-level permitting, social acceptance, or induced-seismicity regulation might slow rollout.

12. The role of advanced heat pumps, CCS with waste heat, or nuclear district heating as competitors is underexplored.

13. The response argues that early heat-based rollout can unlock electricity competitiveness, but the manuscript lacks a robust quantitative link (e.g., explicit scaling of cost reduction with installed heat-only capacity).

(Remarks on code availability)

Reviewer #2

(Remarks to the Author)
I have problems with the paper. I'm not an expert in economic modelling. I have seen that the other reviewers are. I consider me as a technology expert. Therefore, I made comments about missing technology aspects. In the new version, they we're mentioned. However, the answer of the authors was in addition, that these developments are part of the learning curve and they led to cost reduction without going into details how this should function. I know that these new developments have the consequence of deeper drilling but lower seismic problems. That means either increase by deeper drilling or decrease by less seismic events. Therefore, cost reduction may occur but based on a different cost compilation. I wonder if the used cost models can do this. Nevertheless, these influences on the cost models were not presented in the revised paper.

(Remarks on code availability)

Version 2:

Reviewer comments:

Reviewer #1

(Remarks to the Author)
Thank you for addressing the previous comments. Some concerns/comments remain and are listed below.

1. You report a system-wide load-weighted average electricity price of ~88 €/MWh (without EGS) decreasing to ~70 €/MWh under strong EGS cost reductions. Please state explicitly (in the main text) that if these are model-implied prices from the optimized decarbonized system, or observed market prices, and specify the modeled year/scenario context when quoting these numbers.

2. At present, the manuscript does not clearly benchmark EGS economics (LCOE / implied strike price) against current wholesale/retail electricity price ranges in Europe. Without this, readers may over-interpret the “market integration” framing as near-term feasibility. A short, well-cited sentence (or small table) contextualizing modeled prices relative to recent European market prices would materially strengthen the credibility and interpretation.

3. The rebuttal points to an LCOE distribution in supplementary, but the main manuscript still lacks a clear, reader-facing presentation of EGS LCOE ranges/distributions and how those relate to modeled prices. Please include (in the main text and/or a main figure) a concise summary of EGS LCOE distributions used/assumed and how they shift under your cost-reduction cases.

4. The added marginal-price analysis is helpful, but it does not answer whether EGS projects are revenue-adequate under the modeled prices. Please add at least one of the following:
(1)fraction of EGS sites with LCOE ≤ modeled node price,

(2) implied required premium/subsidy (strike price – modeled price), or

(3) net present value / payback indicators (even as distributions).
This would directly address economic feasibility rather than inferring it indirectly from system-average prices.

5. One key request was capacity factor (or operating hours). The manuscript should report utilization/dispatch outcomes for

EGS (e.g., median and interquartile range of annual generation or capacity factor by case). This is necessary to interpret costs and competitiveness, especially since EGS value depends on dispatchability and system need.

6. Cost-reduction assumptions need stronger justification and/or disaggregation. You assume large, uniform reductions to total EGS CAPEX (applied broadly, not component-wise). Please either:

(1) disaggregate CAPEX reductions (drilling vs stimulation vs surface plant), or

(2) provide a stronger evidence-based rationale for applying a blanket scaling to total CAPEX.
As written, the results appear highly sensitive to an assumption that is not yet empirically supported at commercial scale.

7. Avoid overstating feasibility; keep conclusions aligned with “threshold/scenario” nature. The manuscript correctly notes that high drilling costs currently make EGS economically uncompetitive.However, some “market integration pathway” phrasing reads as if large-scale feasibility is imminent. Please tighten the framing to emphasize that your results identify conditions/thresholds under which EGS could become competitive, rather than suggesting competitiveness under current conditions.

8. If your implied EGS LCOE remains well above plausible market prices (today or in-model), that indicates economic non-competitiveness, not that EGS is “not technically feasible.” Please ensure wording throughout distinguishes technical feasibility (can it be built/operated) from commercial feasibility (will it compete without support).

9. Explicitly state the competitiveness threshold implied by your study. You state that electricity-only EGS competitiveness requires drilling costs to fall to ~40% of 2020 levels.This is an important takeaway and should be highlighted clearly in the abstract/discussion, along with the associated uncertainty and realism of achieving that reduction.

(Remarks on code availability)

Reviewer #2

(Remarks to the Author)
The quality of the paper was improved with the last version und my concerns in former reviews were addressed. The restrictions of the findings are given und the reader can now better understand for what the conclusions are given. However, the paper lost focus by the now given structure. It is hard to follow that the conclusions are made and the methods are discribed in a later chapter after the discussions.
Nevertheless, I recommend minor revision of this manuscript because a more focuesd revision requires a totally new written article.

(Remarks on code availability)
I have not tested the code

Version 3:

Reviewer comments:

Reviewer #1

(Remarks to the Author)
Thank you for addressing the comments. I recommend acceptance of the manuscript.

(Remarks on code availability)

Open Access This Peer Review File is licensed under a Creative Commons Attribution 4.0 International License, which permits use, sharing, adaptation, distribution and reproduction in any medium or format, as long as you give appropriate credit to the original author(s) and the source, provide a link to the Creative Commons license, and indicate if changes were made.
In cases where reviewers are anonymous, credit should be given to 'Anonymous Referee' and the source.
The images or other third party material in this Peer Review File are included in the article's Creative Commons license, unless indicated otherwise in a credit line to the material. If material is not included in the article's Creative Commons license and your intended use is not permitted by statutory regulation or exceeds the permitted use, you will need to obtain permission directly from the copyright holder.
To view a copy of this license, visit https://creativecommons.org/licenses/by/4.0/

Author Response to Reviews of

Market Integration Pathways for Enhanced Geothermal Systems in Europe

Lukas Franken, Elisabeth Zeyen, Orestis Angelidis, Tom Brown, Daniel Friedrich
Nature Communications
Revision #1: July 16, 2025

RC X.Y: Reviewer Comment, **AR:** Author Response, *Manuscript Text*

General Reply

We thank the reviewers for their thoughtful and insightful comments. We believe the adjustments that have been made are great improvements to the quality of the work, and we are deeply grateful for the time and effort invested. Beyond responding to each of the comments, the main additions are the following:

- A sensitivity study evaluating a near-carbon-neutral energy supply.
- An LCOE-based analysis assessing the potential competition EGS might face from power-generating gas turbines with carbon capture and storage.
- Broad additions discussing policy, societal concerns, and investment support to deal with the high risk associated with EGS projects.
- A deeper discussion of limitations in the main text.
- A general toning back of language to reflect both the uncertainty about future cost reductions and the approximate nature of the analysis.

With these additions, we hope to have addressed the suggestions made adequately and look forward to the further review process.

We have attached the new iteration of the manuscript, where the changes made are highlighted in red for Reviewer 1, teal for Reviewer 2 and blue for Reviewer 3.

Reviewer 1

General Response to Reviewer 1:

We would like to express our gratitude for the incredible effort invested in this review. It prompted us to add between two and four additional pages to the text, and we believe that the new content really helps to situate the modelling within the wider context of the debate and generally elevates the quality of the article.

RC 1.1 The use of a high-resolution, multi-sector European energy system model (PyPSA-Eur) is appropriate. However, several key assumptions should be better justified.

(i) For example, the decision to prohibit transmission grid expansion is only briefly defended by a sensitivity showing small impact – the authors should provide more evidence or reasoning for this simplification, since transmission constraints can affect renewable vs. local EGS deployment.

(ii) Similarly, the assumptions around district heating rollout (e.g. a 30% progress toward a 60% urban heat share) appear somewhat arbitrary; the manuscript should either cite supporting studies for these specific figures or explicitly test the impact of different heat network expansion rates (some of which is touched on in the sensitivity analysis, but it needs clearer justification in the main text).

(iii) Additionally, the method of assigning EGS costs/potentials to regions by averaging $1^\circ \times 1^\circ$ resource data could mask important local variations – this cost-averaging approach might obscure low-cost "sweet spots" or overestimate poor-resource areas, so the authors should discuss how this spatial aggregation affects results and consider a finer resolution or at least acknowledge the uncertainty it introduces.

AR: We agree that all three points are relevant and, for each, have made the respective adjustments and additions in the text that we believe enhance the quality of the work.

(i) With regard to the transmission grid, we have added additional references that justify the chosen approach. There are two factors driving this decision. First, a large share of future grid investment is allocated to offshore–onshore grid connections, and the model does not contain a precise allocation of the assigned resources. Second, historical and continuing delays in grid reinforcement complicate an assessment of which share of planning will actually materialise. We therefore opt for the conservative approach of modelling within the context of dynamics that currently exist and testing the effect of a sensitivity with a 12.5–25% expansion, reflecting the compounded outcomes of the aforementioned reasons. For that sensitivity, the changes in EGS adoption induced (1–5%) suggest to us that it is reasonable to assume that our findings are robust against likely future transmission grid expansion.

> ***[Added to Results]*** *Transmission capacity expansion would likely benefit EGS roll-out by facilitating the transport of EGS-generated power from regions of high geological suitability. By default, however, for two reasons we have opted to use the incumbent grid capacity and explore additional expansion only as a sensitivity. First, allocating grid investment is difficult to align with our modelling approach; the present model does not explicitly assign the relevant share of future grid capacity investment to onshore-offshore grid connection, which constitutes a large share of investment, complicating a precise tracking of the leeway future grid planning should afford the model. We further assume that past trends of onshore grid reinforcement lagging behind renewable roll-out will continue, and therefore examine sensitivities with an additional 12.5 % and 25 % grid capacity (expressed in GWkm), in line with expectations for the magnitude of future changes [1]. This addition results in a 1–3 % capacity increase for electricity-generating EGS and a 5 % decrease for combined-heat-and-power EGS, leading us to conclude that our findings are largely robust to future transmission-capacity expansion.*

(*ii*) With regard to district heating, we agree that the reasoning behind the chosen numbers could be strengthened and have therefore added the corresponding discussion. We further agree that the assumed district heating shares are highly relevant to the results, which is why the sensitivity analysis explores three additional levels of rollout.

> ***[Added to Results]*** *For regions that currently have low rollout, this would mean that around 20 % of urban heating demand is met by district heating. Conversely, for regions that already have high roll-out, such as the Nordic and Eastern European countries [2, 3, 4], the model essentially assumes today's levels. The 30 % value is therefore mainly relevant for countries with substantial district-heating adoption plans, such as Germany or the UK, where it approximates near- to medium-term targets [5, 6] (see Figure A.18 and the Methods for details).*

(*iii*) In terms of the spatial distribution of the EGS suitability data, we agree that some sweet spots are likely to be missed and that the downstream effects of the rather low spatial resolution are a limitation that should be discussed more in the main text. We have added the corresponding discussion to the main text, but, in terms of updating the modelling, we are unfortunately somewhat constrained by the available data. Therefore, we have added a discussion of how data with improved spatial granularity could be integrated in the future and how such data could be further leveraged by examining how EGS suitability spatially overlaps with heat demand — a consideration that is highly relevant for hard-to-transport heat.

> ***[Added to Discussion]*** *One limitation of the results stems from the spatial resolution of the underlying EGS suitability data ($1° \times 1°$) [7]. A more granular dataset would capture high-suitability "sweet spots" more accurately. The smoothing of EGS suitability across larger regions—as done in the underlying data and carried into this work—is therefore likely to underestimate the true EGS opportunity. Future datasets could enable a more detailed analysis, including a demand-side assessment that checks whether demand centres coincide with such sweet spots and feeds these overlaps into the optimisation model.*

RC 1.2 The assumed drilling cost declines (up to 60–80% reduction from today) and the implied learning rates deserve a more critical examination. The authors adopt cost reduction factors from prior work and later infer a 20–25% learning rate needed for EGS – this is notably aggressive (comparable to solar PV learning rates) for a technology like drilling that historically sees slower gains. It is recommended the authors justify the realism of these cost trajectories: Are there empirical precedents or specific innovations (e.g. advanced drilling techniques, multi-well pads, oil/gas industry spillovers) that support such steep cost declines? It would strengthen the paper to acknowledge potential deployment constraints on learning – for instance, if EGS deployment is initially very limited due to high costs, substantial exogenous support or global deployment (beyond Europe) might be required to achieve those cost reductions. In its current form, the modeling effectively treats cost drops as exogenous scenario inputs; a discussion of investment pacing (how quickly capacity can scale to drive learning) and whether policy-driven initial deployments or international R&D efforts are assumed would add important context. In short, the cost and learning assumptions seem optimistic – the authors

should add caveats or sensitivity cases (e.g. slower learning, or a cap on annual build rate) to ensure conclusions aren't overly reliant on idealized cost reduction pathways.

AR: We agree that the cost trajectory evaluated here is highly optimistic and have removed, adapted, or added sections of the text accordingly. However, these trajectories are neither assumptions nor results on our part; rather, we are merely evaluating under which scenarios, and at which energy outputs, EGS becomes economical and where. In this context, we purposefully chose a broad spectrum of possible cost developments, aligning with costs estimated as plausible in Aghahosseini et al., not because we predict such cost reductions but because they might occur (even though we acknowledge that a five-fold cost reduction does not appear very likely). The paper therefore aims to remain agnostic about the true cost reductions and to provide useful insight regardless of the actual outcomes.

The estimation of cost reduction in Aghahosseini et al. is based on an assumed 7.5% technology learning rate per doubling of installed capacity — a rather small value relative to, for instance, solar power, with roughly 20% [8]. Where Aghahosseini et al. is more optimistic, however, is in the exponentially increasing EGS adoption projected over the coming decades. In our paper, by contrast, the limited European scope (compared with the global scope of Aghahosseini et al.) means that we assess learning rates only on the comparatively limited capacity considered here, increasing the inferred learning rate to 20–25%. This implicitly assumes that technology learning is a local (or at least continental) development — an assumption diametrically opposed to that made in Aghahosseini et al. Upon reflection, we agree that this is misleading. We have therefore removed or clarified the relevant parts of the work to streamline the analysis. We have further changed the text to emphasise that we are not making cost predictions ourselves but are merely aiming to provide insights in light of the broad spectrum of uncertainty concerning the real cost reduction.

Finally, with regard to the learning rate, we have added a discussion about market opportunities outside Europe, both as a response to this and a later comment by Reviewer 1, and we will return to this at a later point.

> ***[Removed from Discussion]*** ~~*From the relationship found between cost reduction and market opportunity, we assess that with a learning rate between 20 and 25%, deployment during the first phase of EGS could suffice to unlock the second phase. This is slightly higher than the 20 % observed for solar generation, but it should, however, be considered in the context of this study's exclusive focus on Europe; EGS cost reductions unlock markets outside Europe, likely leading to less stringent demands on the learning rate.*~~

> ***[Removed from Discussion]*** ~~*During this phase, EGS gains access to a much larger market, which implies lower requirements on the learning rate relative to phase one, converging to approximately 20%. Therefore, it appears likely that if EGS can build the momentum to reach phase two, that momentum could be sustained without additional public funding.*~~

> *[**Added to Discussion**] It remains unclear whether the cost reductions evaluated here can be realised. The range of cost trajectories analysed reflects the values deemed plausible in [7], where technology learning is driven by projected exponential growth in EGS deployment and a 7.5 % cost decrease for each doubling of installed capacity. This assumes idealised learning from increased global uptake but does not yet consider the additional potential of EGS to supply heat. Our present analysis—showing that most early, high-cost EGS market opportunity lies in the heating sector—therefore highlights a learning potential that is under-emphasised in the existing literature.*

RC 1.3 (*i*) The simplifications in how EGS technology is modeled should be more thoroughly justified and, if possible, refined. Notably, the model uniformly sets the geothermal production temperature (150°C) and uses a single efficiency/cost parameterization for power conversion – while this streamlines the analysis, it ignores site-by-site differences in reservoir temperature and conversion efficiency (which in reality range 10–20% for electricity). This uniform approach risks biasing results (e.g. underestimating the benefit of a few high-temperature sites or overestimating performance in marginal areas). The authors themselves note in the limitations that geological performance uncertainties and resource variability are neglected; I suggest explicitly acknowledging in the main text that EGS output and costs likely vary by location and that results are an idealized case.

(*ii*) In terms of operational flexibility, the study commendably explores a "flexible EGS" scenario (injecting fluid to store thermal energy and boost output later) – however, this is presented as an optimistic case given seismicity risks. It would be useful if the authors clarify how EGS is operated in the base scenarios: is it assumed to run as baseload at full output, or can it be dispatchable (e.g. turndown when electricity prices are low)? If not already detailed, the manuscript should describe any dispatch constraints or flexibility assumptions for EGS (beyond the special 24h storage case).

(*iii*) Finally, the co-generation modeling appears to allow EGS plants to supply both heat and power, which is a strength of the study – the authors might want to ensure they explain how the model optimizes the split between heat vs. electricity output. For instance, is the ratio fixed or can the plant allocate thermal output dynamically based on demand?

Overall, while the technical representation is simplified, it is mostly reasonable for a system model – but the manuscript should more clearly state these simplifications (temperature, efficiency, dispatch limits) and perhaps discuss the implications (e.g. slightly overstated EGS potential if real-world operational downtimes or performance variations were considered).

AR: The reviewer raises three valid points, and we have amended the text accordingly.

(*i*) With regard to modelling a uniform temperature assumption, the main reason lies in the underlying geothermal-potential data shared by Aghahosseini *et al.* The optimisation conducted in that paper, weighing up drilling cost against surface-plant cost, results in a spatially resolved map of cost-optimal temperature and depth. However, that data layer was not shared alongside the EGS CAPEX values and was therefore not available for our analysis; the same applies to the breakdown of CAPEX into drilling and surface-plant shares (parts of these data were presented in the original paper but were not included when the author exchanged his results with us

at higher spatial resolution). Consequently, we are unable to determine exactly which share of the cost should be removed for heat-generating EGS and must therefore assume a uniform temperature and, hence, a uniform surface-plant cost (the cost share removed from the total cost when considering heat generation only). This has different implications for different output types.

For electricity generation, the power output seen by the energy model comes from a chain of components – injection well, production well and organic Rankine cycle. Through our approximation, the split of the cumulative CAPEX of the chain into its components is imprecise. However, for electricity generation the model sees only the total CAPEX of the chain; the temperature-sensitive CAPEX values from the underlying data are therefore passed through unchanged and should not introduce additional error.

For heat generation, the uniform-temperature approximation does induce an error, resulting in the dynamic outlined by the reviewer: the results underestimate favourability in highly suitable regions and overestimate it in less suitable ones.

Overall, the lack of available temperature data and the temperature sensitivity of the electricity-only generation data make us hesitant to overhaul the chosen temperature approximation fundamentally, and we hope the reviewer agrees that, in this case, the scale of intervention required outweighs the likely benefit.

The default values chosen – 150°C and an electricity-generation efficiency of 12% – are based on what we judge to be a reasonable average of Figure 8 in Aghahosseini *et al.* Temperatures at optimal depth appear to hover between 150°C and 175°C for Europe; however, generation efficiency seems closer to 10%, which we consider low for that temperature band, prompting us to favour 150°C.

In the paper, the discussion of these dynamics was not sufficiently clear, and we have amended the Methods section accordingly. Further, as suggested, we have added passages describing the relevant errors induced into *combined heat-and-power* and *heat-only* EGS in the main text.

> ***[Added to Discussion]*** *In heat-only EGS, the capital cost of the organic Rankine cycle (ORC) is removed, leaving only the drilling-related component. The cost split between drilling and the ORC depends on borehole depth and reservoir temperature. However, the modelling assumes a uniform geothermal temperature of 150°C (following [7]) and therefore does not account for spatial variation in this split. In highly suitable regions the model likely overestimates the drilling cost share and thus slightly overstates total EGS costs, while the opposite holds in less suitable areas. This effect is most relevant in the low- to moderate-cost-reduction cases, where EGS faces stronger competition.*

> ***[Added to Methods]*** *This decision is informed by [9], which finds that EGS plants in Germany typically generate both heat and power but achieve far higher full-load hours for heat than for electricity, indicating that the systems modulate between the two.*

(*ii*) Indeed, EGS is assumed to be dispatchable by default, with additional flexibility explored in the sensitivity analysis. This was not sufficiently clear in the original text, and we have added the corresponding discussion. The difference between the two modes is the continued pumping through the injection well in the flexible case. We have amended the main text to discuss this distinction in more detail. With regard to the associated risk of seismicity, contrary to what the text previously implied, both dispatchable and flexible operation appear to entail such risk owing to the ramping of the system's throughput. We have added more recent guidelines and interventions; in particular, we note that softer ramping is likely to make such operating strategies plausible options for present and future EGS plants.

> ***[Added to Results]*** *Flexible operation differs from regular, assumed-dispatchable EGS [10] by allowing operation at a lower partial load during periods of high levels of intermittent generation and then, after pressure and temperature in the reservoir have increased, generate beyond the plant's nameplate capacity. However, the resulting increase in reservoir pressure has, in the past, been associated with the risk of inducing seismicity [11]. However, more recent work has shown that gentler ramping of injection rates and other interventions can mitigate this risk [12, 13], and it therefore appears highly likely that present and future EGS plants will be able to modulate back-pressure to temporarily boost generation. The same interventions can also be applied to dispatchable EGS, where sudden ramping can likewise be a risk factor.*

> ***[Added to Methods]*** *Finally, throughout this work EGS is assumed to operate as a dispatchable generation source by default. Although originally considered a baseload technology, more recent studies show that pausing and resuming production is feasible [14, 15, 16]. We note that dispatchable operation is distinct from flexible operation, in which the production well ceases to flow while the injection well continues, building reservoir pressure and enabling a boost in generation once production resumes [17]. The economic benefits of such flexible operation are analysed in the sensitivity analysis.*

(*iii*) We see the reviewer's perspective and agree that the paper does not clearly explain that the relative capacities of heat and power generation in CHP EGS plants are fixed, whereas the hour-to-hour operational decisions are not. This modelling decision was made on the basis of many CHP EGS plants in Germany that appear to supply heat for a high number of full-load hours but generate power only during a rather small subset of hours. The Methods section previously addressed the first point only briefly, and we have now expanded that discussion.

> ***[Added to Methods]*** *This decision is informed by [9], which finds that EGS plants in Germany typically generate both heat and power but achieve far higher full-load hours for heat than for electricity, indicating that the systems modulate between the two.*

RC 1.4 The scenario range could be expanded to better capture key uncertainties and alternative futures. The authors focus on varying drilling costs (which is central) and perform sensitivities on district heating expansion, renewable costs, grid, etc., but it would strengthen the study to test or

discuss additional scenarios.

(i) For example, one critical uncertainty is district heating rollout speed: the manuscript's base assumption is moderate growth, but a scenario with delayed or minimal heat network expansion (beyond today's levels) is important to consider – the authors do include a "zero-progress" case, but this result should be highlighted in the discussion (e.g. noting that EGS uptake falls significantly if heat networks don't expand). Conversely, exploring an aggressive district heating scenario (or highlighting the one they did with 60%–100% urban coverage) helps bound the potential.

(ii) Another missing dimension is alternative firm capacity solutions: the current model appears to exclude options like new nuclear or fossil CCS, meaning EGS (plus storage and hydrogen) fills the firm supply gap. The paper would be more convincing if it at least acknowledged or tested a case where other firm low-carbon technologies are available (e.g. a scenario with nuclear or gas+CCS in the mix) – this would show how EGS competes in a broader technology landscape and ensure the results aren't an artifact of a limited option set.

(iii) Additionally, the manuscript assumes a fully net-zero 2050 system; discussing a partial decarbonization pathway (where climate targets are weaker or reached later) could be valuable, since EGS economics might differ if carbon prices are lower or if gas heating persists longer. In summary, the scenario exploration would be improved by covering a wider uncertainty envelope – for instance, varying the policy context (strong climate policy vs. moderate), heat demand electrification vs. district heating emphasis, and even availability of other firm renewables (like bioenergy or long-duration storage). These additions would give readers more confidence that the conclusions about EGS's role hold under different plausible futures, not just the specific setup chosen.

AR: We agree with the reviewer that important sensitivities are either not sufficiently explored or do not receive enough attention in the Discussion. We have added two additional sensitivities — the inclusion of gas CCS and a near-carbon-neutrality scenario — and have expanded the existing text regarding the sensitivity on district-heating.

(i) We agree with the reviewer that the Discussion section previously only echoed the sensitivity findings with regard to district-heating rollout that falls short of expectations. We have added a few sentences that highlight this potential outcome and further emphasise the generally quite extreme linkage between district-heating rollout and the (modelled) EGS market opportunity.

> ***[Added to Discussion]*** *Conversely, if the roll-out of district heating surpasses current projections, particularly in countries with large heat markets but limited existing networks, the potential market for EGS could expand substantially. If for instance, 30 % of all heating demand was met by central supply, it likely would increase cost-optimal EGS borehole capacity by around 150 GW_{th} relative to our base scenarios of moderate progress (Fig A.18). In short, whether European district-heating deployment falls short of or exceeds expectations, the implications for the EGS market would be significant, and therefore an attempt to track EGS's future market requires precise tracking of district heating development.*

(ii) We have supplemented our study with an LCOE-based comparison between EGS and gas-fired generation equipped with carbon capture and storage (gas CCS), rather than modelling

both technologies explicitly in PyPSA-Eur. Because the two options are dispatchable and their future costs are highly uncertain, the LCOE framework allows us to explore a broader range of plausible parameter combinations more efficiently. Our results show that under sustained low-gas-price scenarios, gas CCS could feasibly compete with electricity-producing EGS. Consequently, the evolving cost trajectories of both technologies merit close monitoring.

> ***[Added to Results]*** *We have further considered competition from alternative dispatchable, carbon-neutral sources, in particular gas generators with carbon capture and storage. However, because gas CCS is not constrained by geographic suitability, shares dispatchability characteristics with EGS, and is subject to large cost uncertainty, we opt for a simpler LCOE comparison (Fig 1). A straightforward LCOE calculation for a 30-year lifetime, 7 % discount rate, 47 % efficiency, and 50 % capacity factor—together with CAPEX assumptions of 2,000–2,900 €/kW [18, 19]—places such dispatchable generation at values comparable to EGS, with additional sensitivity to gas price. In a low-gas-price scenario (15 €/MWh [20]) gas CCS could generate carbon-neutral power at a cost similar to that required for EGS adoption in the model. Under a more pessimistic price assumption (30 €/MWh [21]), however, gas CCS appears largely uncompetitive with the EGS capacities adopted by the model. Similar to EGS, gas CCS is mostly at the demonstration stage, and substantial uncertainties remain regarding its feasibility—particularly its currently much lower-than-expected capture rates [22], with the additional cost of rectifying these shortcomings still unclear. In summary, gas generators with CCS could plausibly compete with EGS as a source of firm, carbon-neutral power; however, the combined uncertainties surrounding both technologies complicate any direct comparison, and future work should track the performance of gas CCS demonstration projects relative to those for EGS.*

> ***[Added to Discussion]*** *Potential competition from gas turbines with carbon capture and storage (CCS) is assessed via a simple LCOE comparison (30-year lifetime, 7 % discount rate, 47 % efficiency, 50 % capacity factor, and 2 000–2 900 €/kW CAPEX). At low gas prices (∼15 €/MWh) gas-CCS can match EGS costs, but at ∼30 €/MWh it becomes uncompetitive; moreover, real-world capture rates lag expectations, adding further uncertainty. Thus gas-CCS could eventually vie with EGS for firm, carbon-neutral power, but the substantial techno-economic unknowns surrounding both technologies warrant close monitoring of forthcoming demonstration projects.*

(*iii*) Finally, the carbon-neutrality constraint is a strong assumption by default and could have implications for EGS's market opportunity. We have added the corresponding sensitivity to the Results, where we explore an upper bound on emissions of 10% relative to 1990 levels (Fig. 2). We thank the reviewer for this nudge, because it highlights interesting new findings. It turns out that EGS loses market opportunity not to emitting competitors in district heating but rather by relinquishing some of its role as a facilitator of deep decarbonisation. In particular, the relaxed carbon constraints lead the model to produce less hydrogen and undertake less direct air capture, lowering EGS market opportunity by between a third and a half, as discussed here:

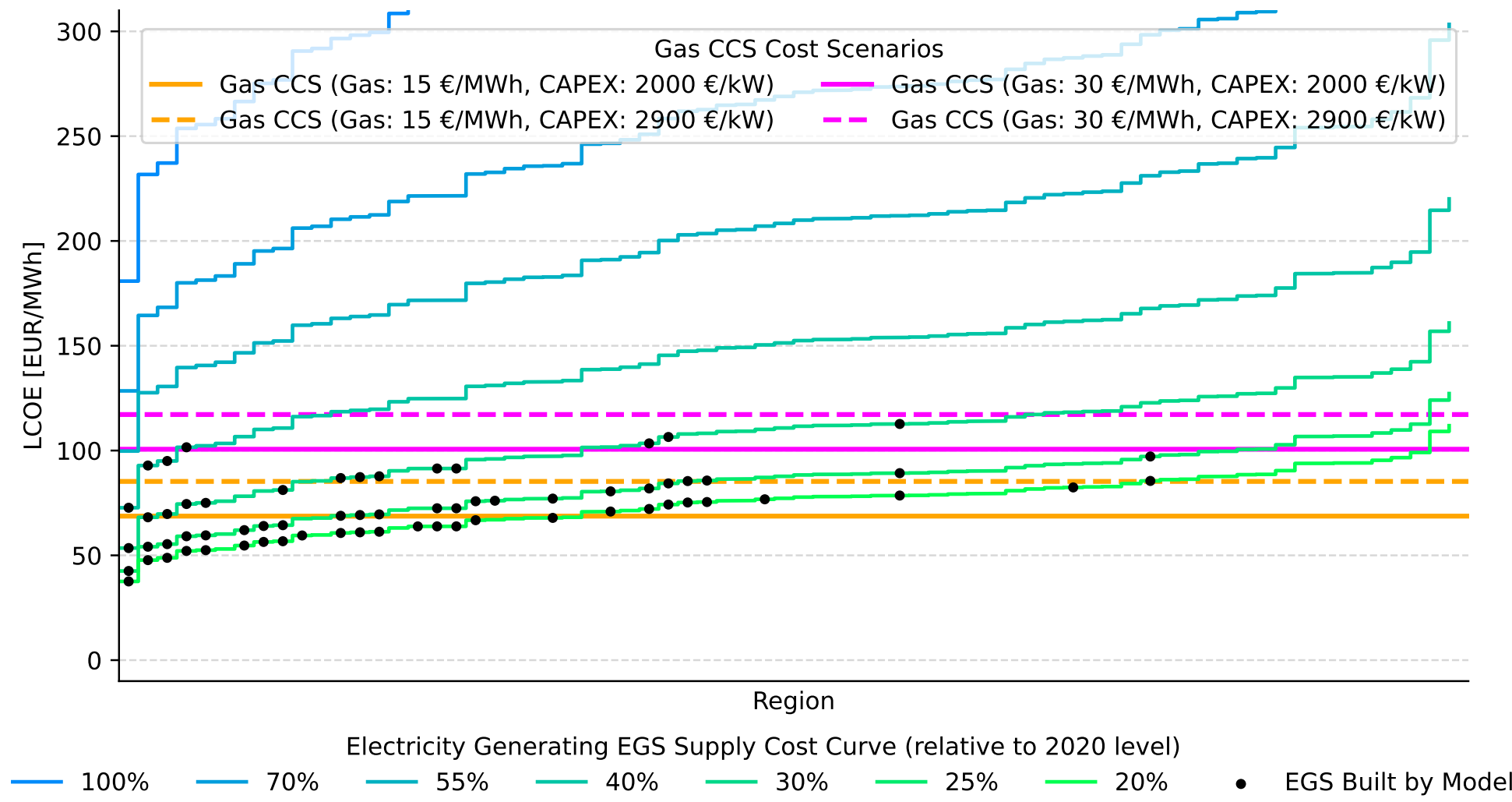

Figure 1: Comparison of LCOE values from [7] used in the model to different projections for gas generators with attached carbon capture and storage. The gas generators are assumed to operate at an efficiency of 47% [18] and a capacity factor of 50%.

> ***[Added to Results]*** *Default model runs assume a fully carbon-neutral system. As a low-carbon technology, this constraint benefits EGS, but we find that it does so in a somewhat surprising way. In a sensitivity that relaxes the carbon-neutrality target, allowing emissions up to 10 % of 1990 levels (instead of 0 %), we find EGS capacity expansion reduced by roughly 1800, 90, and 50 GW_{th} for electricity-, co-generating, and heat-only EGS, respectively—eliminating about half of the market opportunity. However, in all three cases we find that this is not the result of stronger competition for the demands EGS serves; rather, its role as a facilitator of deep decarbonisation diminishes. Specifically, the low-grade heat that EGS supplies inexpensively lowers the cost of direct air capture, while electricity demand is amplified by H_2 electrolysis—both downstream effects of the perfect-decarbonisation assumption in the default setup.*

> ***[Added to Discussion]*** *The analysis shows that EGS derive much of their value from enabling very deep decarbonisation: In the baseline model, a zero-emissions constraint allows EGS to provide low-grade heat, which lowers the cost of direct air capture, and electricity that supports large-scale hydrogen electrolysis. When the carbon cap is relaxed to 10 % of 1990 levels, EGS capacity falls by half—not because of new competitors, but because the system no longer needs those decarbonisation services.*

RC 1.5 Real-world deployment of EGS will face geographical and societal constraints that are not fully captured in the model's least-cost optimization. The manuscript should more directly acknowledge factors like regulatory barriers, permitting hurdles, and social acceptance issues that could constrain where EGS projects can actually be built. For instance, certain regions with high geothermal potential (or even existing pilot projects) have experienced public opposition or moratoria due to induced seismicity concerns (e.g. Germany's restrictions after seismic events). Are the identified

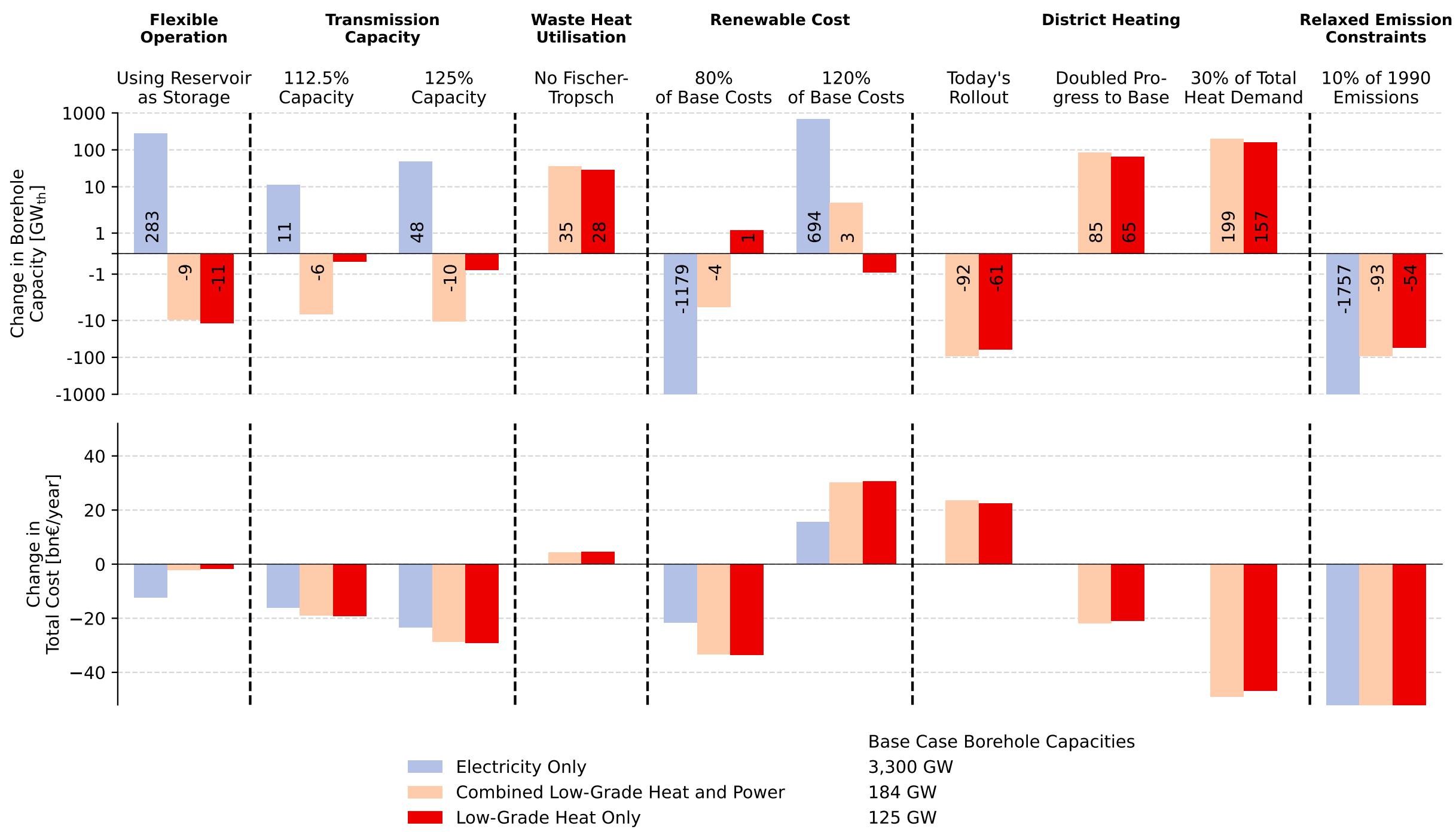

Figure 2: The change in borehole capacity and total system cost is evaluated for given sensitivities relative to EGS base cases, which include EGS under the same system assumptions as outlined in the previous sections. The base cases for heat-generating EGS are scenarios with a cost level of 40% relative to 2020 for heat (co-)generating EGS, and 20% for electricity-generating EGS.

"high-opportunity" regions for EGS in the results actually likely to allow large-scale deployment? If not, that could significantly reduce practical potential. The authors are encouraged to integrate these external constraints qualitatively (at minimum) or even quantitatively. One suggestion is to discuss a scenario or sensitivity where EGS deployment is restricted in some densely populated or high-seismic-risk areas – how would the system cope (perhaps via more of other firm sources)? Even if not modeled explicitly, the paper should emphasize that permitting and public acceptance could slow the rollout: e.g. lengthy project lead times, the need for community engagement, and possible exclusion of certain regions. Additionally, regulatory frameworks (like drilling regulations, subsurface rights, or environmental impact requirements) vary by country and could delay projects beyond what an optimized model assumes. By addressing these geographical and societal constraints, the authors will provide a more nuanced outlook – highlighting that the theoretical potential from the model is an upper bound contingent on favorable regulatory and public environments. Integrating such insights (perhaps in the discussion or a dedicated subsection) will make the analysis more realistic and policy-relevant.

AR: We agree with the reviewer that these constraints exist and were not sufficiently emphasised in the manuscript. To address induced-seismicity risk, regulators and industry in Europe and the United States have introduced mitigation protocols that require extensive additional monitoring. The cost data set of Aghahosseini *et al.* omits these monitoring expenses; integrating them would raise levelised costs by a non-trivial amount. Nevertheless, in the heat-supply segment that currently dominates the EGS market, even modest drilling-cost reductions would likely offset most of this increase. An alternative strategy is to locate EGS plants outside urban areas and compensate by using longer heat-transport pipelines. Unlike monitoring, the extra capital required for extended heat transmission could materially undermine EGS competitiveness. Because the market impact of seismic-risk constraints depends on local fault-line distributions, a geographically resolved assessment is required. We have added a paragraph to the *Discussion* section to flag these points and to emphasise the need for future work on the spatial implications of seismic-risk mitigation.

[Added to Discussion] [Also aims to address Reviewer 1 Comment 7, and Reviewer 3 Comment 1] *EGS roll-out could also face societal constraints that are not captured by the modelling framework. Public opposition to EGS increased notably after fracking-related events in which induced seismicity activated existing fault lines, leading to noticeable ground shaking, widespread building damage, and project abandonment—as in Basel, Switzerland, in 2006 [23], and even more severely in Pohang, South Korea, where a magnitude 5.5 earthquake was triggered [24]. To avert such events, frameworks have been proposed in both Europe and the United States to accompany EGS roll-out; they include seismic-risk pre-screening and the monitoring of risk indicators during drilling, stimulation, and operation [13, 25, 26].*

[Added to Discussion] [Also aims to address Reviewer 3 Comment 1] *These protocols are likely to lead to only a minor overall cost increase or, irrespective of such protocols, to discourage EGS deployment in urban settings. On the one hand, these cost additions are small relative to the cost range evaluated here, particularly for heat-generating EGS, which appears to hold a sizeable advantage over alternative carbon-neutral heat supplies. Future work could, however, examine the implications of public opinion*

restricting EGS adoption to rural areas. To interpret the findings in light of these potential additional costs, we suggest reading, for example, Manuscript Figure 3 with an additional shift along the x-axis representing the corresponding cost increase.

RC 1.6 (i) The manuscript would benefit from a more cautious interpretation of results to ensure findings are not overstated. In several places, the language could be tempered to clarify that outcomes are conditional on assumptions (e.g. cost reductions, policy support, etc.). For instance, when discussing EGS becoming competitive in electricity markets with ∼60% cost reduction, it should be clear this is an "if/then" result rather than a prediction – i.e., "if drilling costs drop by X, then EGS could expand by an order of magnitude," rather than implying it will definitely happen. It is suggested adding such conditional phrasing and explicitly noting that these scenarios represent optimistic, best-case conditions for EGS (especially since a net-zero system inherently favors it).

(ii) Additionally, some results might be currently framed in a way that implies greater certainty than warranted. For example, the paper quantifies system cost savings and optimal capacities to high precision; given the uncertainties, the authors should emphasize these numbers are illustrative estimates under the model's conditions.

(iii) It would also help to highlight any important caveats in the main text (not just in the limitations section). For example, investor behavior and financing realities are not captured in the model's perfect-foresight least-cost approach – so the real-world investment in EGS might lag even if it's "optimal" on paper. A clear statement that market-optimal deployment does not guarantee investor willingness or immediate feasibility would prevent overinterpreting the capacity results. By more explicitly framing conclusions with their caveats (cost uncertainty, model simplifications, policy dependencies), the authors can avoid readers or policymakers drawing overly deterministic conclusions from what are scenario-based insights.

AR: (i) We fully agree with the reviewer that our language overstates the likelihood that the substantial cost reductions we evaluate will materialise, while offering no evidence to support such an outcome. Working with "when" instead of "if" clearly implies certainty—an implication that is, in retrospect, unwarranted. Passages that sound predictive rather than conditional have therefore been amended throughout the Abstract, Results, Discussion and Conclusion. In some instances, instead of repeatedly using "would" and "if", we now refer to "scenarios" with high cost reductions, so as to avoid writing the entire text in the second conditional. We have opted not to quote these changes here individually.

(ii) We agree that some findings were reported in greater detail than the underlying methodology justifies. If we have not overlooked anything, the main culprits are the percentages given early in the Results section, previously reported to two decimal places. We have rounded these figures to whole percentages and, at the end of the Discussion, have added a remark to the effect suggested: the reported numbers are coarse approximations contingent on the modelling framework.

***[Added to Discussion] [also aiming to address** (iii)**]** We also emphasise that the modelling presented here is subject to considerable uncertainty—both with regard to the simplifications required to accommodate the study's scale and to unknown future cost trajectories. Therefore, the reported numbers are approximations and should be interpreted accordingly.*

> *One such simplification is that the model overlooks the investment risk that an EGS plant may under-perform if the geothermal resource delivers less output than expected. While subsidies and policy interventions to mitigate this risk are discussed, the model assumes that such mechanisms are—and remain—available to shield investors. Further limitations are discussed in the Methods.*

(*iii*) We agree that the modelling neglects the additional cost of risk, and, in fact, this omission is explicitly acknowledged. Together with point (*ii*), this has now been made explicit in a new paragraph in the *Discussion* section of the text. Policies that mitigate that risk have also been added to the text and will be discussed later in this Response.

RC 1.7 The paper currently underplays the practical hurdles associated with large-scale EGS deployment, and it should dedicate more attention to these aspects. Enhanced Geothermal Systems often face public and regulatory challenges – notably, permitting delays, stringent regulation on induced seismicity, and local public opposition due to safety or environmental concerns. The authors briefly mention that expanding other renewables can face public opposition, but they should equally note that EGS is not immune to this: past EGS projects (e.g. in Basel or Pohang) have been halted by induced earthquakes, leading to public wariness. Does the analysis assume that all proposed EGS capacity can be built on schedule? If so, that may be optimistic given the likelihood of lengthy permitting processes (drilling approvals, environmental impact assessments) and possible moratoria if seismic events occur. Including a discussion on how policy and public engagement would be needed to address these risks (for example, improved micro-seismic monitoring, compensation schemes, or community benefit agreements) would balance the technology optimism with real-world prudence. In summary, the manuscript should acknowledge that even if EGS becomes economically attractive, non-economic barriers could slow its adoption – and these need proactive management. By addressing this, the authors ensure the reader understands the difference between economic potential and realizable capacity on the ground.

AR: We agree that practical hurdles, particularly those relating to public and regulatory challenges, are insufficiently discussed in the manuscript and that, distinct from Comment 5, they affect not only costs but could have a more dramatic impact: a seismic incident might provoke opposition severe enough to render an EGS project—or the technology as a whole—untenable. We have partially addressed this comment together with Comment 5 by highlighting past seismic events, particularly Basel and Pohang, in a dedicated paragraph; we then added a paragraph discussing the cost implications of such events (thereby addressing Comment 5) and, for Comment 7, added another paragraph outlining the more "dramatic" risks of public opposition and the regulatory hurdles associated with seismicity risk.

> ***[Added to Discussion]*** *Beyond additional costs, future seismic events—which may occur again—could deal a substantial blow to EGS roll-out and introduce hurdles that are hard to quantify in monetary terms. Even in its current state, past events have already raised significant practical barriers to EGS deployment. Moreover, public opinion remains largely unformed about EGS owing to its niche status [27]; by contrast, fracking is widely known and a majority of*

people—particularly within liberal urban cohorts—hold an unfavourable view of it [28]. In this context, rigorous seismic monitoring and environmental-impact assessments are essential to maintain public trust during roll-out. These requirements can lead to delays and additional costs that are difficult to capture in the present modelling and occur against the backdrop of a continuing risk that any serious future incident could render EGS politically untenable.

RC 1.8 The scenarios suggest a very rapid scale-up of EGS capacity once it becomes cost-competitive (potentially hundreds of GWth in a couple of decades), and the feasibility of this pace should be discussed. Rolling out EGS at the modeled scale would require an enormous ramp-up in supply chains, skilled workforce, and drilling infrastructure. Currently, only a handful of EGS projects exist in Europe; reaching even the "first phase" ∼20–30GWth identified would mean drilling hundreds of deep wells and building out many combined heat/power facilities in a relatively short period. It is recommended that the authors explicitly comment on whether this expansion rate is realistic. Are there enough drilling rigs and crews available? How quickly can the industry grow while maintaining safety and quality? Potential bottlenecks like the availability of drilling equipment, rigs, cementing crews, and even the manufacturing of power turbines or heat exchangers for EGS plants could significantly slow deployment in practice. It would strengthen the manuscript to mention that achieving the modeled trajectory would likely require coordinated efforts to expand the industrial capacity (perhaps analogous to how the shale gas/fracking boom required scaling up rig counts). If the authors have any insights from industry reports or historical analogues (e.g. the growth of the oil & gas drilling sector or wind industry ramp-up), they could briefly include those; if not, a general caution that "the model does not account for maximum build rates or supply-chain limitations, which could delay reaching these capacity levels" would suffice. Acknowledging this limitation ensures readers appreciate that the timeline of EGS adoption might be much more gradual than the model's equilibrium solution, absent extraordinary mobilization.

AR: We agree with the reviewer that, for an emerging technology, the practicality of the envisaged scale-up warrants careful scrutiny. Accordingly, we have added a section that benchmarks the deployment volumes assumed for EGS against the current global stock of drilling rigs and related equipment. Our analysis indicates that—provided existing rigs are not already operating at full utilisation—the present fleet could support a substantial expansion of EGS capacity. By contrast, large-scale electricity-generating EGS projects could tighten the market for specialised rigs and auxiliary equipment. In the near term, therefore, hardware bottlenecks seem unlikely; yet a successful scale-up will still require sustained transfer of skills and supply-chain capabilities from the oil and gas industry.

[Added to Discussion] *The market opportunity discussed here exceeds the cumulative installed capacity of European EGS projects many-fold. Even under the more conservative cost-reduction scenarios, the identified 20–30 GWth opportunity exceeds the roughly 100 MWth operating EGS capacity in Europe [29, 30, 31, 32, 33] by orders of magnitude. Assuming each well doublet provides around 10 MWth, realising that capacity would require thousands of wells. Drilling times vary considerably; for instance, Utah reached a depth of 5 km in 16 days [34], whereas a Bavarian project in 2003 required almost 150 days [35]. As a back-of-the-envelope*

> *calculation, one drilling rig with an average well-drilling time of 60 days could add about 30 MWth per year. At that rate, developing 30* GW_{th} *would demand roughly 100 rig-years if 10 rigs were deployed—or 10 years with 100 rigs. Given the current European fleet of about 125 drilling rigs [36], this underscores both the practical importance of rig availability in any large-scale egs roll-out and the critical role of technology and skill transfer from the oil-and-gas industry.*

RC 1.9 The manuscript's results have clear policy implications, but the discussion could do more to translate them into actionable recommendations. Since the study indicates district heating expansion and cost reductions are key to EGS viability, policymakers should be informed about which levers to pull. For instance, carbon pricing is implicitly crucial (the model assumes carbon-neutrality); the authors could emphasize that a strong carbon price or equivalent regulatory measure is needed to make EGS (and other low-carbon heat options) competitive against fossil heat. Likewise, enabling EGS's contribution to heat implies supportive district heating policies – such as funding for heat network infrastructure, mandates or incentives for connecting buildings to communal heating, and urban planning that accommodates geothermal heat sources. The paper should also highlight geothermal-specific risk mitigation policies that exist or could be expanded: for example, government-supported insurance schemes for drilling (to de-risk the resource uncertainty), R&D programs for advanced drilling tech, or "green heat" tariffs. By detailing such policies, the authors can guide readers on how to catalyze the first phase of EGS deployment. Currently, the text hints at coordination being crucial but stops short of specifics; it would be valuable to articulate that coordination could take the form of an EU-wide geothermal innovation program or shared knowledge networks to achieve the learning rates identified. In summary, making the market and policy relevance more explicit – e.g., "our findings suggest that without interventions like X or Y, the modeled outcomes may not materialize" – will make the paper more useful to decision-makers and strengthen its impact.

AR: We thank the reviewer for the comment and use this rather broad comment about policy to create a summary of the policy implications that the reviewer raised, both in comments before and after this one, along with comments raised by other reviewers. In particular, we have added that summary to the Conclusion section of the paper. The summarised policies, most of which were added to the paper because of this review, are as follows:

- Policies to ensure third-party access to district heating are crucial, as is stimulating sufficient district heating demand.
- The visualisation of the carbon-neutral pathway is essential (include results from sensitivity analyses).
- Regulation must enforce practices that minimise seismicity risk; if tremors occur, negative public opinion towards fracking could spill over and make EGS politically untenable.
- Scaling up EGS may create practical bottlenecks in drilling expertise and equipment: although rig demand is moderate relative to Europe's current stock, large-scale market penetration could require broader supply chain upscaling.
- Investment structures and loans should socialise the high-risk phases of EGS develop-

ment—early geological exploration, first-of-a-kind installations, and protection against poor reservoir performance.

- Regulatory requirements should be streamlined to minimise delays and hurdles for developers.

> ***[Added to Conclusion]*** *In terms of policy, the measures enacted in recent years are crucial for further EGS development, yet additional bottlenecks may still impede progress if left unaddressed. Even under deep-decarbonisation scenarios—and in the heating sector in particular—the bulk of EGS potential depends on steep cost reductions; policy must therefore foster the technology learning required to achieve them. Ensuring open third-party access to district heating networks and stimulating sufficient heat demand are foundational steps that have not yet been rolled out across the European Union. At the same time, carbon markets or targeted subsidies should reward the carbon neutrality of EGS, as an increasing number of countries are beginning to do. Regulators must also rigorously enforce practices that mitigate seismic risk, because any induced tremors could provoke public backlash and render EGS politically untenable. Investment instruments and loans should socialise early geological and first-of-a-kind risks to shield investors from poor reservoir performance. Further, streamlining permitting and other regulatory procedures would cut delays and lower entry barriers for EGS developers. Finally, while current European drilling-rig capacity is moderate, a rapid EGS scale-up could strain rigs, expertise, and the wider supply chain, making proactive upscaling essential.*

RC 1.10 The economic barrier of upfront investment for early EGS projects is high, and the manuscript should address how those might be overcome. At current costs, even though the model finds some EGS is cost-competitive for heat, private investors might be reluctant due to the high capital risk and unproven nature of EGS in many regions. It is recommended that the authors discuss or cite mechanisms to finance initial projects – for example, public funding or guarantees for first-of-a-kind (FOAK) EGS plants, dedicated EU or national grants/loans, or public-private partnerships to share risk. If the model's results rely on cost reductions via learning, there is an implicit need for upfront "loss-leading" investments; the paper should make this explicit by suggesting that early deployment will likely require subsidies or risk underwriting (since waiting for costs to drop on their own may never happen without initial volume). The authors could strengthen the discussion by referencing how other technologies (offshore wind, solar, etc.) saw rapid cost declines: nearly all had significant government support in early phases (feed-in tariffs, tax credits, etc.), which may also be necessary for EGS. Additionally, the concept of an "investment trap" could be mentioned: EGS won't get cheaper until it's deployed at scale, but it won't deploy at scale until it gets cheaper – breaking this deadlock likely requires policy intervention. By including these points, the manuscript will be able to better address the financeability of EGS, reassuring readers that the authors have considered the real-world economics and not just the model optimality. In practical terms, suggestions such as geothermal risk insurance funds, drilling cost-sharing programs, or targeted capital grants would show what enabling measures align with the study's findings.

AR: We agree with the reviewer that these topics were not adequately discussed in the text and have **closed this gap**. Upon researching the topic, we were **pleased** to find that **de-risking**

strategies, as suggested by the reviewer, are indeed in place on a broad scale. Most of these schemes appear to result from European or **pan-European** directives, and they support (deep) geothermal projects exactly in the way considered by the reviewer. Indeed, it appears that the rollout of such schemes largely aligns with the findings of the paper and is mostly **focused** on Eastern, Northern and Central Europe. Accordingly, we have added a paragraph on the topic to the Discussion, giving an overview of the policies in place and highlighting the dynamics they are aiming to unlock—breaking the deadlock, similar to how "escape velocity" was achieved for other renewable energy carriers.

> ***[Added to Discussion]*** *Further policy support should protect investors against the risks inherent in engineering geothermal reservoirs and developing exploratory, first-of-a-kind projects, similar to the de-risking strategies employed during the early roll-out of wind generation. Such policies are increasingly being implemented across European countries. Some programmes aim to socialise the costs of high-risk test wells [37, 38, 39], while others more broadly target support for early-stage technologies [40]. These incentives could serve as a powerful lever to break the "investment trap", whereby high geothermal costs hinder widespread adoption and limited adoption in turn slows technology learning.*

RC 1.11 The integration of EGS into energy markets (both electricity and heat) may necessitate changes in market design, and the paper should touch on this to round out the policy discussion. For the electricity market, if EGS eventually provides a significant firm capacity (especially in the high cost-reduction scenario), current energy-only markets might undervalue its contribution to reliability. The authors should consider noting that mechanisms like capacity payments or adequacy markets could reward EGS's ability to deliver steady power, ensuring it's financially viable to build even if energy prices are depressed by abundant renewables at times. Similarly, valuing ancillary services (frequency support, grid stability) could improve the economics of EGS power plants if they run flexibly – did the model account for any ancillary service provision? If not, it's worth mentioning that in reality EGS could have additional value streams. On the heat market side, the manuscript already identifies district heating as crucial; the authors might elaborate that heat markets often lack transparent pricing or open competition. They could recommend market reforms or regulations for heat: for example, ensuring that district heating networks allow third-party geothermal heat injection on fair terms, or implementing heat purchase agreements (analogous to PPAs in power) to guarantee offtake for geothermal heat. Moreover, if EGS plants are to optimize co-generation, the coordination between power and heat markets must be considered (e.g. pricing such that operators have incentive to produce the most needed form of energy at a given time). Including a brief commentary that market rules need to evolve – for instance, integrating heating into energy planning, or providing long-term contracts for firm low-carbon capacity – would demonstrate that the authors have thought through the practical integration of EGS beyond just technical feasibility. Such discussion can be kept concise but will significantly enhance the paper's relevance to energy planners and regulators.

AR: We agree with the reviewer that policy regarding market frameworks could be supportive in improving EGS's market opportunity. We have therefore added two paragraphs to the Discussion. The first discusses the role that capacity markets could play for electricity-generating EGS,

potentially relaxing some of the requirements for extreme cost reductions by rewarding its firm generation capability. We have also highlighted frequency response as a potential second revenue stream, but noted that forthcoming field trials will clarify the technical feasibility of this option. The second paragraph concerns heat markets, where we have explored the topic in greater depth and provided an overview of how recently passed or forthcoming regulation in Europe aligns with the conclusions drawn from our work and facilitates the general rollout of geothermal energy.

> ***[Added to Discussion]*** *Additional revenue streams via electricity market frameworks could ease the need for drastic cost reductions by rewarding EGS for its ability to provide firm electricity [16]. The modelling used here implicitly assumes an energy-only market. However, the ongoing renewable roll-out can create unfavourable market conditions for dispatchable resources (through highly unstable spot prices), especially before demand-side flexibility has caught up [41]. To ensure the availability of such resources, countries have introduced mechanisms, such as capacity markets, that reward firm generators for complementing variable renewables [42]. Such incentives would benefit electricity-generating EGS. Additional revenue streams could also arise for EGS if field trials demonstrate sufficiently rapid dispatch adjustments to support grid stability through frequency response [16].*
>
> *In the context of heating, European regulation is already moving to shape district-heat markets in ways that support the roll-out of EGS. For instance, district-heat markets are regulated local monopolies, and market access for new heat generators differs from the electricity sector. However, recent work shows that, across varying degrees of national regulatory stringency, renewable generation sources can still diffuse into district-heat markets [43]. Moreover, policy support for heat-generating EGS is gaining momentum: the European Union's Renewable Energy Directive 2023/2413 prohibits district-heating systems >25 MWth from monopolistic gate-keeping and encourages instruments such as heat-purchase agreements [44]. As of summer 2025, the German government is preparing legislation to accelerate geothermal project planning [45].*

RC 1.12 The manuscript engages with existing studies [2–4,15,16, etc.], but a more thorough differentiation could strengthen its positioning. It would be beneficial for the authors to explicitly contrast their findings with those of earlier works. For instance, if Longa et al. [4] found ∼25 GWe of EGS in an IAM and this study finds a comparable magnitude (or perhaps more when including heat), the authors should discuss why – e.g., "Longa et al. assumed optimistic cost but no spatial detail, whereas our results show a similar order-of-magnitude potential concentrated in specific regions under certain conditions." Also, the paper might consider citing additional relevant literature on geothermal integration or firm renewables for completeness. There have been recent energy outlooks (e.g., IEA or IRENA scenarios, IPCC reports) which typically project limited geothermal uptake; referencing these and then explaining why this study might diverge (due to inclusion of heat co-generation or different cost assumptions) would contextualize the work. If any studies on district heating and geothermal (perhaps from a European Commission report or national plans) exist, citing them could back the importance of heat integration. The authors mention EGS co-producing lithium [16] – this is a good example of broadening context. They could also mention any known demonstration projects or national targets for EGS to show awareness of the landscape. Ensuring all statements like "widely

seen as an ingredient of cost-efficient heat supply" have appropriate references will guard against unsupported claims. Overall, the literature review is on the right track; a few more references or clearer comparisons – especially highlighting what past models missed (e.g., high temporal resolution for flexibility, explicit learning rate analysis, or pan-European coordination issues) – will underline the manuscript's originality and thoroughness.

AR: We agree with regard to the addition of further references and have expanded the paragraph on related modelling literature, particularly that concerning broader energy-landscape assessments, not exclusively geothermal (e.g. IEA and IRENA scenarios, and IPCC reports). Accordingly, the Motivation section was adjusted to trace their low estimates of geothermal potential back to the overlooked possibility of drilling-cost reductions—the scenario analysed here. We have also added literature references to the Discussion and related our findings to those of Longa *et al.* Furthermore, in addressing a previous review comment, we elaborated on justifying the model's assumptions about future district-heating rollout. Therefore, with regard to the present comment, we have included the reference added at that point in the passage highlighted by the reviewer in the Methods.

> ***[Added to Introduction]*** *However, broader and often influential energy outlooks that consider the entire energy mix—rather than geothermal specifically—arrive at more pessimistic projections for its deployment, notably the IEA's* World Energy Outlook 2023 *[46], IRENA's* 2050 Energy Outlook *[47], and the IPCC* Sixth Assessment Report *(2022) [48].*
>
> *[...]*
>
> *Meanwhile, studies that do not focus on geothermal supply—such as the IEA* World Energy Outlook 2023 *[46]—favour only a pessimistic EGS cost outlook, neglecting the fact that, as a technology with limited current roll-out, it still offers considerable scope for learning-driven cost reductions.*

> ***[Added to Discussion]*** *This finding broadly aligns with previous results for electricity-generating EGS under optimistic drilling-cost scenarios reported by Longa et al. [49], which project around* $25\,GW_{el}$ *of capacity, while also offering additional context on its spatial distribution and the conditions under which this market opportunity arises.*

RC 1.13 the manuscript's framing should stress the wider significance of the findings beyond the immediate European case. The authors can elevate the impact by drawing out generalizable lessons: for example, the idea that a niche market (heat) can serve as a springboard for a new technology to later become mainstream in electricity is a concept that could apply to other regions or technologies. They should make it clear that while the study is set in Europe, the insights on EGS learning and integration could inform geothermal strategy in other parts of the world (e.g. how combining heat & power applications might make EGS viable in Asia or North America's decarbonization efforts). In the introduction or conclusion, a sentence or two linking the results to global decarbonization (perhaps noting that deep geothermal could provide a similar firm renewable option in regions with suitable geology, or that the challenges identified – cost reduction, heat infrastructure, public acceptance – are

common everywhere) would broaden the paper's appeal. Additionally, the framing could be sharpened by emphasizing the relevance to current energy policy debates: for instance, many countries are now seeking firm, dispatchable clean energy to complement wind/solar – this study provides timely insight into where and when EGS might fill that role. The authors might also consider a slightly more accessible summary of the core message in the conclusion: e.g., "Our analysis indicates that enhanced geothermal could evolve from a small heat-providing niche today into a major player in future power systems, but only if substantial cost reductions are achieved through coordinated deployment and supportive policy." Such a statement distills the significance for a broad scientific readership. Finally, to maximize visibility, the authors should ensure the title and abstract clearly reflect the most striking outcomes (currently they do convey the key points quite well). Emphasizing keywords like "net-zero energy system," "firm renewable energy," and "techno-economic learning" in the framing can also help position the paper at the forefront of energy systems research.

AR: We thank the reviewer for this helpful nudge. Upon researching this point, we found that, in terms of both low-temperature industrial and district-heat demand, the rest of the world—particularly China and Russia—holds substantial potential for district heating, amounting to several times that of Europe; the same relative magnitude applies to low-temperature industrial demand. We have added a paragraph to the Discussion, highlighting that, for this demand, geothermal could—through the same dynamics that played out in our modelling—find another large market opportunity. In the same vein, we liked the suggested sentence and have added it to the Conclusion. However, the results did not appear to yield sufficiently straightforward evidence for us to discuss at length the particular role that EGS could have in meeting the need for firm generation (beyond comparing its potential future LCOE with gas-CCS LCOE), given that most of that potential derives either from extreme cost reductions or from flexible operation, which we feel we did not investigate in sufficient depth to justify an elaborate discussion.

> ***[Added to Results]*** *We note that this is a local estimate; if technology learning occurs at a pan-European scale, the process could draw on the much larger market opportunities outside Europe, thereby substantially lowering the required learning rates (see Discussion).*

> ***[Added to Discussion]*** *The results have further implications beyond Europe, where district- and low-temperature industrial heat demands are substantially larger. For instance, China and Russia each exceed 4,500 TWh of annual district heating demand, while the rest of the world accounts for more than 1,500 TWh [50]. Meanwhile, the vast majority of low-temperature industrial heat demand—globally totalling around 9,000 TWh [51]—is also located outside Europe. Currently, most of this demand is met by fossil fuelled heat generation [50], but, given incentives for low-emission heat and moderate drilling-cost reductions, it could provide additional market opportunities for EGS, mirroring the dynamics observed in the present study.*

> ***[Added to Conclusion]*** *Our analysis indicates that EGS could evolve from a small, heat-providing niche today into a major player in future energy systems—but only if substantial cost reductions are achieved through coordinated deployment and supportive policy.*

Reviewer 2

General Response to Reviewer 2:

We thank the reviewer for this highly valuable suggestion. Recent developments at the Blue Mountain and UtahFORGE sites have changed the framing of EGS cost reductions, as they demonstrate tangible progress beyond the cost baseline used here, thereby making the paper's assumption of steep cost reductions more plausible.

RC 2.1 I missed the recent technical developments of EGS, e.g. in Utah or Blue Mountain (see Geothermal Power Generation (Second Edition), Developments and Innovation, Woodhead Publishing Series in Energy 2025, Pages 451-473 Enhanced Geothermal Systems: Review and Status of Research and Development) (Xing et al., 2023), (Norbeck et al., 2023). EGS is not equal EGS and the way of multi stage stimulation has a significant influence on the energy output and therefore on LCOE. I recommend to include such technological aspects in a revised manuscript.

AR: We thank the reviewer for this highly valuable suggestion. We find that it provides extremely relevant context to the text. In particular, it appears that the "multi-stage lateral reservoir stimulation"—as demonstrated at the projects in **Nevada** and **Utah**—has already translated into an LCOE that is approximately 25 %[10] lower than the values we previously labelled as the 2020 baseline (our case of no cost reductions).

This implies that the 2025 cost level should not be understood as equivalent to the 2020 figure, but rather as approximately $\sim 80\,\%$ of that benchmark. Based on this, we modified the text in two respects: (*i*) an explicit discussion of the Nevada and Utah demonstration sites, and (*ii*) a reframing of the cost narrative to signal that part of the learning curve has already been realised.

These insights have been incorporated into the revised manuscript. Concretely, we have made the following additions:

1. We extended the literature review to feature the Nevada and Utah sites as contemporary proof-of-concept examples.
2. We inserted a vertical guideline in Figure 1 to mark the estimated cost decline already achieved since 2020.
3. We adjusted passages of the main text to emphasise that present-day EGS costs should be interpreted as post-learning values—about 80 % to 75 % of the 2020 level—rather than as the pre-learning 2020 benchmark.

> *[**Added to Introduction**] Recent technical advances highlight the ongoing cost reductions of EGS technology. A shortcoming of conventional reservoir-stimulation methods in EGS is the high probability that the induced fracture network will lack sufficient permeability for economically viable circulation. Field campaigns conducted between 2020 and 2025 at the Blue Mountain and UtahFORGE sites have validated a multistage "plug-and-perf" strategy: Instead*

of propagating a single massive fracture, each horizontal well is treated in roughly a dozen discrete stages, generating a laterally extensive fracture network. This approach has delivered greater injectivity and improved thermal sweep efficiency [52, 53, 54] and is projected to reduce the levelised cost of electricity (LCOE) by about 25% [10].

[Added to Discussion] *However, in light of the recently demonstrated advanced stimulation techniques at the Blue Mountain and UtahFORGE sites, both conducted after 2020, it appears plausible that as of 2025 already, 20-25 % cost reductions has already been achieved [10].*

[...]

However, our baseline 2020 estimate of EGS cost [7] anticipated a roughly 25 % reduction by 2025—a decline that data from active demonstration sites suggests has indeed materialised [10].

Reviewer 3

General Response to Reviewer 3:

We thank the reviewer for the thoughtful comments and have done our best to add the context they requested, which we hope enriches the text.

RC 3.1 The paper focuses primarily on EGS costs as the main driving factor for market opportunities. However, other factors might play an important role as well for EGS adoption, for example, social acceptance and - especially - the induced seismicity. The seismicity factor should be addressed and discussed in the paper.

AR: We agree with the reviewer that the existing manuscript insufficiently considers a range of factors that evade direct economic measurement. Seismicity appears to be chief among these factors, and we have added two paragraphs to the Discussion: first, to explore historic EGS-induced seismic events and the associated public backlash; and second, to outline protocols that aim to minimise the risk of inducing such events.

[Added to Discussion] [Also aims to address Reviewer 1 Comment 5] *EGS roll-out could also face societal constraints that are not captured by the modelling framework. Public opposition to EGS increased notably after fracking-related events in which induced seismicity activated existing fault lines, leading to noticeable ground shaking, widespread building damage, and project abandonment—as in Basel, Switzerland, in 2006 [23], and even more severely in Pohang, South Korea, where a magnitude 5.5 earthquake was triggered [24]. To avert such events, frameworks have been proposed in both Europe and the United States to accompany EGS roll-out; they include seismic-risk pre-screening and the monitoring of risk indicators during drilling, stimulation, and operation [13, 25, 26].*

These protocols are likely to lead to only a minor overall cost increase or, irrespective of such

protocols, to discourage EGS deployment in urban settings. On the one hand, these cost additions are small relative to the cost range evaluated here, particularly for heat-generating EGS, which appears to hold a sizeable advantage over alternative carbon-neutral heat supplies. Future work could, however, examine the implications of public opinion restricting EGS adoption to rural areas. To interpret the findings in light of these potential additional costs, we suggest reading, for example, Manuscript Figure 3 with an additional shift along the x-axis representing the corresponding cost increase.

RC 3.2 Methodology: all model runs use only single mode of EGS operation. However, especially in the context of spatial distribution across Europe, it would also make sense to consider different EGS modes at the same time and let the model 'decide' where to install each EGS type (mode).

AR: We agree with the reviewer that such a framing would have been an interesting addition to the work. Ultimately, however, we decided against including it for two reasons. First, for clarity of discussion, we believe that only one of the two possible implementation approaches had space in the manuscript. Second, and more importantly, we are not confident that the statements such an analysis would yield can be supported by the rather coarse approximations inherent in large-scale modelling and the uncertainties in our cost assumptions. In particular, if the technology costs differ only slightly between, say, heat-only and combined-heat-and-power EGS, the model may adopt only one of the two. However, real planners would base their decisions on a much more granular assessment of demand-side and geological opportunities in the context of uncertain technological developments. Therefore, we opted for the slightly more defensive and clearer approach.

Regardless, if future work improves the available data on EGS suitability, this could, in our view, motivate an assessment that allows different types of EGS to compete.

RC 3.3 Methodology: the paper does not clearly state the modelling year (if there is one) for the net-zero Europe energy system. This year, e.g. 2050, will influence vast majority of model parameters, such as projected energy demands, costs of other technologies, including renewables, etc. It is important to clarify this more.

AR: We agree with the reviewer that the modelled year is highly relevant to the results, and we have added the corresponding discussion to the text. With regard to technology roll-out, the modelled year is 2050; this primarily affects fuel switching in road transport and shipping, as well as in steel and aluminium production. We chose 2050 mainly because the carbon-neutral constraint, when paired with a residual presence of internal-combustion engines in land transport, can otherwise force the model into unrealistic configurations that require vast volumes of synthetic fuel. For renewable-cost inputs, we adopted the 2030 projections—a default choice in many PyPSA-Eur studies.

Both of these points have been elaborated upon in the main text and the Methods.

[Added to Introduction] *The model's background assumptions are exogenously set to align*

with the carbon-neutral constraint. That means, for instance, that fuel switching in road transport, shipping, and industrial heating is fixed at the levels projected for 2050.

[Added to Methods] *In terms of technology roll-out assumptions, the model year is set to 2050 to align with the carbon-neutral target. Specifically, we assume 85 % electrification of road transport, modifications to steel and aluminium production processes, a high degree of building retrofits to reduce space-heating demand, and a complete fuel switch in shipping from oil to methanol. We further assume that renewable-energy costs decline in line with Danish Energy Agency projections for 2030 [55], representing an average reduction of roughly 25 % relative to 2020 levels.*

RC 3.4 Methodology: related to 3) - cost reductions of other technologies are not addressed sufficiently. Only in the sensitivity analysis, there is analysis of cost reductions of renewable by +/- 20%. Since this has a major impact on the overall results, more analysis and investigation regarding cost reductions is needed. For example, what are the potential cost reductions (projections) for other technologies, especially renewables, and how do the compare to EGS case? What is the historical development here?

AR: We agree with the reviewer that renewable cost reductions are needed to provide a realistic competitive backdrop for assessing EGS's market opportunity. As the reviewer points out, this was an oversight on our part: we had not made our default cost assumptions sufficiently clear. We have elaborated on this in response to Comment 3, noting (both here and in the corrected manuscript) that the default costs for all technologies except EGS are projections for the year 2030 (mostly produced by the Danish Energy Agency) in an effort to strike a balance between today's costs and the cost reductions likely to materialise in future. We have made further additions to the text to communicate this more clearly.

In this context, we have elaborated with an additional sensitivity analysis. While a broader assessment of alternative cost developments could yield further insights, we believe the range already assessed suffices for the qualitative statements we aim to make. First, heat-(co-)generating EGS at moderate cost reductions is extremely robust against changes in renewable costs, highlighting the particular opportunity the heating sector offers for EGS. Meanwhile, the market opportunity of electricity-generating EGS is highly susceptible to renewable costs. In light of general cost uncertainty, we would argue that the precise numbers behind this dynamic are less important than the qualitative picture of how competition changes as EGS progresses through successive cost-reduction levels.

Prompted by this point and Reviewer 1 Comment 4, we have also added an LCOE-based comparison between EGS and potential cost developments for gas turbines with carbon capture and storage, which we hope will prove helpful in addressing this comment. For convenience, we have included that addition in the discussion here as well.

*[**Added to Results**] We have further considered competition from alternative dispatchable, carbon-neutral sources, in particular gas generators with carbon capture and storage. However, because gas CCS is not constrained by geographic suitability, shares dispatchability characteristics with EGS, and is subject to large cost uncertainty, we opt for a simpler LCOE comparison (Fig 1). A straightforward LCOE calculation for a 30-year lifetime, 7 % discount rate, 47 % efficiency, and 50 % capacity factor—together with CAPEX assumptions of 2,000–2,900 €/kW [18, 19]—places such dispatchable generation at values comparable to EGS, with additional sensitivity to gas price. In a low-gas-price scenario (15 €/MWh [20]) gas CCS could generate carbon-neutral power at a cost similar to that required for EGS adoption in the model. Under a more pessimistic price assumption (30 €/MWh [21]), however, gas CCS appears largely uncompetitive with the EGS capacities adopted by the model. Similar to EGS, gas CCS is mostly at the demonstration stage, and substantial uncertainties remain regarding its feasibility—particularly its currently much lower-than-expected capture rates [22], with the additional cost of rectifying these shortcomings still unclear. In summary, gas generators with CCS could plausibly compete with EGS as a source of firm, carbon-neutral power; however, the combined uncertainties surrounding both technologies complicate any direct comparison, and future work should track the performance of gas CCS demonstration projects relative to those for EGS.*

RC 3.5 In the context of 4) and multiple other assumptions (with uncertainties) in the model - such as assumptions on pg. 11 about EGS capital expenditure, the cost of heat integration, etc. - it makes more sense to provide the main results as ranges (distributions) rather than single values. Using single value results, such as 60% cost reduction as tipping point, can be misleading in this context.

AR: We fully agree with the reviewer that many of the reported numbers suggest a level of detail in the modelling that is at odds with the model's rather coarse approximations. Accordingly, we have adjusted the language throughout the text, ensuring, in particular, that percentage values are not quoted at a precision finer than whole integers.

RC 3.6 In addition to this, there are other major limitations explained in section "Limitations", which further make uncertain the overall results - and limit the statements. This is especially the case for neglecting the temporal nature of energy transition. While these are discussed in the section, they were not reflected anywhere before in the paper - this needs to be further addressed.

AR: We agree that the manuscript is not sufficiently forthright in communicating its limitations in the main text.

In response to this point, and to a comment from Reviewer 1, we have added a general overview of the work's principal limitations to the main text. In addition, we have included a brief discussion of the carbon-neutrality constraint adopted throughout the analysis, acknowledging it as a limitation while also providing justification for its use.

*[**Added to Discussion**] We also emphasise that the modelling presented here is subject to*

> *considerable uncertainty—both with regard to the simplifications required to accommodate the study's scale and to unknown future cost trajectories. Therefore, the reported numbers are approximations and should be interpreted accordingly. One such simplification is that the model overlooks the investment risk that an EGS plant may under-perform if the geothermal resource delivers less output than expected. While subsidies and policy interventions to mitigate this risk are discussed, the model assumes that such mechanisms are—and remain—available to shield investors. Further, the model uses a fully carbon-neutral system as a default, neglecting that EGS cost reductions rather proceed in parallel to gradually decreasing emissions of the European energy system. While this oversimplifies the process of decarbonisation, it does provide a streamlined analysis while also, at least in the heating and power sector, largely reflects a cost landscape that in the real system is already created through environmental subsidies. Further limitations are discussed in the Methods.*

RC 3.7 Model: Looking at Fig A.10 or A.11, it can be seen which technologies were modeled in the framework. Some technologies, that might be crucial, are not represented, for example: shallow geothermal systems (open and closed-loop) as well as 'classical' deep geothermal systems. Both of these technologies are, for example, important in countries such as Germany, especially with the planned new capacities. Are the technologies considered in the model? If not, why?

AR: We agree with the reviewer that, from those figures, it appears these technologies have been overlooked. Indeed, the authors have reassured themselves that at least ground-source heat pumps are included in the model. They are, but they are not shown in the figures. Figures A.10–A.12 explicitly exclude technologies that meet service-sector or rural heat demands, as these are, for EGS, the least relevant part of the system. However, in the model only rural heat demands are considered to provide sufficient space for ground-source heat-pump installations.

With regard to existing deep-geothermal systems, these do indeed constitute a relevant part of the energy supply; however, existing capacity appears small relative to the future potential. Our goal in this study is to develop a broad picture of the cost reductions required for EGS to find a market in different parts of Europe. The analysis therefore aims to be agnostic about the particular type of (deep) geothermal in which a stakeholder is interested. Regardless of the type, drilling CAPEX values could be compared with the drilling costs assessed here to gain insight into the achievable market opportunity.

RC 3.8

1. Fig A.14 shows Albania in the model as region 1, however this region is 'empty' in Fig A.13
2. Title of Fig A.9. - please pay attention to grammar
3. Pg. 11: it is mention 'we simulate ... ORC' and 'simulation of borehole operation' - this is misleading. More proper would be 'we model'.
4. Pg. 10: 'Our default assumption is a progress of 30% ...' - based on what?
5. Pg. 4: OCGT - it was not explained before what this is

6. Pg. 4: '(co-)gen-' and then it was skipped to new row
7. Pg. 3: word 'which' goes outside of the 'text frame' - this happens throughout the paper and needs to be fixed

AR: We thank the reviewer for the detailed hints and have corrected the issues raised.

1. After further inspection, it turns out that the underlying EGS data do not overlap with Albania. A corresponding remark has been added to the figure caption.
2. The grammar has been adjusted.
3. We have revised the language in the passages identified.
4. This point was indeed not sufficiently justified, but it does align with European-level planning for district heating. The relevant discussion has been added to the Discussion (see Reviewer 1, Comment 4).
5. We have added the full term, spelt out as "open-cycle gas turbine".
6. We located the passage in question and have corrected the issue.
7. Line breaks have been fixed throughout the text.

References

[1] S. Butorac, Eu electricity grids, Briefing PE 772.854, European Parliamentary Research Service, accessed 25 June 2025 (May 2025).
URL
https://www.europarl.europa.eu/RegData/etudes/BRIE/2025/772854/EPRS_BRI(2025)772854_EN.pdf

[2] T. S. Amirkhizi, M. G. A. Guddat, Organisational structures in the danish district heating sector, PlanEnergi for Deutsche Energie-Agentur (dena) (2024).

[3] M. Bryant, "the heat you need at a reasonable price": how district heating can speed the switch to clean energy, The Guardian (2025).

[4] Poland: Energy district-heating industry needs up to $100 billion for transformation, International Trade Administration, U.S. Department of Commerce (2024).

[5] Starke wärmenetze für eine sichere versorgung – wie klima, haushalte und versorger profitieren (2024).

[6] Department for Energy Security & Net Zero, Heat network zoning: Consultation summary (2023). URL https://www.gov.uk/government/consultations/proposals-for-heat-network-zoning-2023/heat-network-zoning-consultation-summary

[7] A. Aghahosseini, C. Breyer, From hot rock to useful energy: A global estimate of enhanced geothermal systems potential, Applied Energy 279 (2020) 115769.

[8] International Energy Agency, Evolution of solar PV module cost by data source, 1970–2020, https://www.iea.org/data-and-statistics/charts/evolution-of-solar-pv-module-cost-by-data-source-1970-2020, interactive chart in *Clean Energy Innovation*; last updated 2 July 2020. Licence: CC BY 4.0 (2020).

[9] S. Eyerer, C. Schifflechner, S. Hofbauer, W. Bauer, C. Wieland, H. Spliethoff, Combined heat and power from hydrothermal geothermal resources in germany: An assessment of the potential, renewable and sustainable energy reviews 120 (2020) 109661.

[10] M. J. Aljubran, R. N. Horne, Power supply characterization of baseload and flexible enhanced geothermal systems, Scientific Reports 14 (2024) 17619. doi:10.1038/s41598-024-17619-0.
URL https://www.nature.com/articles/s41598-024-17619-0

[11] N. Deichmann, D. Giardini, Earthquakes induced by the stimulation of an enhanced geothermal system below basel (switzerland), Seismological Research Letters 80 (5) (2009) 784–798.

[12] M. Alghannam, R. Juanes, Understanding rate effects in injection-induced earthquakes, Nature communications 11 (1) (2020) 3053.

[13] W. Zhou, F. Lanza, I. Grigoratos, R. Schultz, J. Cousse, E. Trutnevyte, A. Muntendam-Bos, S. Wiemer, Managing induced seismicity risks from enhanced geothermal systems: A good practice guideline, Reviews of Geophysics (2024). doi:10.1029/2024RG000849.
URL https://doi.org/10.1029/2024RG000849

[14] J. Nordquist, T. Buchanan, M. Kaleikini, Automatic generation control and ancillary services, GRC Transactions 37 (2013) 761–766.

[15] W. Ricks, K. Voller, G. Galban, J. H. Norbeck, J. D. Jenkins, The role of flexible geothermal power in decarbonized electricity systems, Nature Energy (2024) 1–13.

[16] R. Horne, A. Genter, M. McClure, W. Ellsworth, J. Norbeck, E. Schill, Enhanced geothermal systems for clean firm energy generation, Nature Reviews Clean Technology (2025) 1–13.

[17] W. Ricks, J. Norbeck, J. Jenkins, The value of in-reservoir energy storage for flexible dispatch of geothermal power, Applied Energy 313 (2022) 118807.

[18] U.S. Energy Information Administration, Electricity, Coal, and Renewables Long-Term Modeling Team, Discussion on capital cost and performance characteristic estimates for new generating technologies, Technical report, U.S. Energy Information Administration, Washington, D.C., accessed 11 July 2025 (Sep. 2023).
URL https://www.eia.gov/

[19] L. Mancuso, N. Ferrari, Effects of plant location on the costs of co_2 capture, Technical Report 2018-04, IEAGHG, Cheltenham, UK (Apr. 2018).
URL https://ieaghg.org/publications/technical-reports/2018-04

[20] A. Exarheas, Executives reveal where they see henry hub price landing in futurePublished 8:00 AM EST; accessed 11 Jul 2025 (Jul. 2025).
URL https://www.rigzone.com/news/executives_reveal_where_they_see_henry_hub_price_landing_in_future-09-jul-2025

[21] International Energy Agency, World energy outlook 2024: Executive summary, Flagship report, International Energy Agency, Paris, France (Oct. 2024).
URL https://www.iea.org/reports/world-energy-outlook-2024

[22] Institute for Energy Economics and Financial Analysis (IEEFA), Carbon capture and storage: An unproven technology that cannot meet planetary co_2 mitigation needs, Briefing note, IEEFA, includes analysis of 16 CCS projects and cites IPCC AR6 Synthesis Report (2023) (Dec. 2023).
URL https://ieefa.org/articles/carbon-capture-and-storage-unproven-technology-cannot-meet-planetary-co2-mitigation-needs

[23] N. Deichmann, D. Giardini, Earthquakes induced by the stimulation of an enhanced geothermal system below basel (switzerland), Seismological Research Letters 80 (5) (2009) 784–798. doi:10.1785/gssrl.80.5.784.
URL https://doi.org/10.1785/gssrl.80.5.784

[24] W. L. Ellsworth, D. Giardini, J. Townend, S. Ge, T. Shimamoto, Triggering of the pohang, korea, earthquake (mw 5.5) by enhanced geothermal system stimulation, Seismological Research Letters 90 (5) (2019) 1844–1858. doi:10.1785/0220190102.
URL https://doi.org/10.1785/0220190102

[25] E. Majer, J. Nelson, A. Robertson-Tait, J. Savy, I. Wong, Protocol for addressing induced seismicity associated with enhanced geothermal systems, Tech. rep., Office of Energy Efficiency and Renewable Energy (EERE), Washington, DC . . . (2012).

[26] D. Bruhn, E. Huenges, K. Ágústsson, A. Zang, G. Kwiatek, X. Rachez, S. Wiemer, J. D. Van Wees, P. Calcagno, T. Kohl, et al., Geothermal engineering integrating mitigation of induced seismicity in reservoirs–the european geiser project, Geotherm Res Council Trans 35 (2011) 1623.

[27] M. McKasy, S. K. Yeo, J. S. Zhang, M. A. Cacciatore, H. W. Allen, L. Y.-F. Su, Support for regulation of enhanced geothermal systems research: examining the role of familiarity, credibility, and social endorsement, Geothermal Energy 13 (2025). doi:⟨pending⟩.
URL ⟨pending⟩

[28] A. Tyson, B. Kennedy, How americans feel about hydraulic fracturing for oil and gas, Pew Research Center – Short Reads, accessed 9 July 2025 (oct 2024).
URL ⟨pending:inserttheexactPewResearchCenterlinkhere⟩

[29] T. Kölbel, A. Genter, Enhanced geothermal systems: The soultz-sous-forêts project, in: Towards 100% Renewable Energy, Springer, 2017. doi:10.1007/978-3-319-45659-1_25.
URL https://www.researchgate.net/publication/313885169

[30] A. Richter, Vulcan resumes geothermal district heating supply in landau, germanyAccessed 9 Jul 2025 (Apr. 2025).
URL
https://www.thinkgeoenergy.com/vulcan-resumes-geothermal-district-heating-supply-in-landau-germany/

[31] A. Richter, Vulcan energy resources acquires 4.8 mw insheim geothermal plantAccessed 9 Jul 2025 (Dec. 2021).
URL
https://www.thinkgeoenergy.com/vulcan-energy-resources-acquires-4-8-mw-insheim-geothermal-plant/

[32] G. Ravier, Rittershoffen geothermal heat plant – enhanced geothermal system (egs) for heat generation,

https://www.geoenvi.eu/wp-content/uploads/2020/04/2020_04_27_Rittershoffen_plant_Guillaume.pdf, webinar slide deck, 27 Apr 2020. Accessed 9 Jul 2025 (2020).

[33] E. Gaucher, T. Kohl, Why the seismicity induced in soultz-sous-forêts and gross schoenebeck enhanced geothermal fields are so different?, in: AGU Fall Meeting Abstracts, Vol. 2013, 2013, pp. NS23C–1596.

[34] V. Addison, Fervo energy sets geothermal drilling records with appraisal well in utahAccessed 2025-07-09 (2025).
URL
https://www.hartenergy.com/exclusives/fervo-energy-sets-geothermal-drilling-records-appraisal-well-utah

[35] T. Hettkamp, J. Baumgärtner, R. Baria, A. Gérard, T. Gandy, S. Michelet, D. Teza, Electricity production from hot rocks, in: Proceedings of the Twenty-Ninth Workshop on Geothermal Reservoir Engineering, SGP-TR-175, Stanford University, Stanford, California, 2004, workshop dates: January 26–28 2004.

[36] YCharts, European union rig count (i:eurr)Monthly rig-count time series; accessed 2025-07-10 (2025).
URL https://ycharts.com/indicators/european_union_rig_count

[37] F. Rojas, France launches $54 million geothermal risk mitigation fundAccessed: 2025-07-06 (2015).
URL https://www.thinkgeoenergy.com/france-launches-54m-geothermal-risk-mitigation-fund/

[38] C. Cariaga, Kfw and munich re introduce risk protection mechanism for geothermal in germanyAccessed: 2025-07-06 (2024).
URL https://www.thinkgeoenergy.com/
kfw-munich-re-introduce-risk-protection-mechanism-for-geothermal-in-germany/

[39] C. Cariaga, Poland to allocate funding for geothermal exploration in ten communesAccessed: 2025-07-06 (2024).
URL https:
//www.thinkgeoenergy.com/poland-to-allocate-funding-for-geothermal-exploration-in-ten-communes/

[40] International Energy Agency, Enova and the climate and energy fund, IEA Policies and Measures Database, policy in force since 2001; country: Norway; last updated 6 November 2019 (2019).
URL https://www.iea.org/policies/enova-and-the-climate-and-energy-fund

[41] T. Brown, F. Neumann, I. Riepin, Price formation without fuel costs: The interaction of demand elasticity with storage bidding, Energy Economics (2025) 108483.

[42] UK Department of Energy and Climate Change, Electricity market reform: Capacity market – detailed design proposalsAccessed: 2025-07-06 (2013).
URL https://assets.publishing.service.gov.uk/media/5a7cb2ebed915d6822361fe3/
15398_TSO_Cm_8637_DECC_Electricity_Market_Reform_web_optimised.pdf

[43] A. Billerbeck, B. Breitschopf, J. Winkler, V. Bürger, B. Köhler, A. Bacquet, E. Popovski, M. Fallahnejad, L. Kranzl, M. Ragwitz, Policy frameworks for district heating: A comprehensive overview and analysis of regulations and support measures across europe, Energy Policy 173 (2023) 113377.

[44] European Parliament and Council, Directive (eu) 2023/2413 of the european parliament and of the council of 18 october 2023 amending directive (eu) 2018/2001, regulation (eu) 2018/1999 and directive 98/70/ec as regards the promotion of energy from renewable sources, and repealing council directive (eu) 2015/652, Official Journal of the European Union, L series (31 October 2023), accessed: 2025-07-06 (2023).
URL https://eur-lex.europa.eu/legal-content/EN/TXT/?uri=CELEX:32023L2413

[45] Bundesregierung, Entwurf eines gesetzes zur beschleunigung der genehmigungsverfahren von geothermieanlagen, wärmepumpen und wärmespeichern sowie zur Änderung weiterer rechtlicher rahmenbedingungen für den klimaneutralen ausbau der wärmeversorgungAccessed: 2025-07-06 (2024).
URL https://www.bundesrat.de/SharedDocs/drucksachen/2024/0401-0500/435-24.pdf

[46] IEA, World energy outlook 2023, International Energy Agency (2023).

[47] International Renewable Energy Agency (IRENA), Global energy transformation: A roadmap to 2050 (2019 edition), accessed: 2025-07-06 (2019).
URL https://www.irena.org/publications/2019/Apr/Global-Energy-Transformation-A-Roadmap-to-2050-2019Edition

[48] Intergovernmental Panel on Climate Change, Chapter 6: Energy systems, in: P. R. Shukla, J. Skea, R. Slade, A. A. Khourdajie, R. van Diemen, D. McCollum, M. Pathak, S. Some, P. Vyas (Eds.), Climate Change 2022: Mitigation of Climate Change. Contribution of Working Group III to the Sixth Assessment Report of the Intergovernmental Panel on Climate Change, Cambridge University Press, Cambridge, United Kingdom and New York, NY, USA, 2022, accessed: 2025-07-06. doi:10.1017/9781009157926.008.
URL https://www.ipcc.ch/report/ar6/wg3/downloads/report/IPCC_AR6_WGIII_Chapter_06.pdf

[49] F. Dalla Longa, L. P. Nogueira, J. Limberger, J.-D. van Wees, B. van der Zwaan, Scenarios for geothermal energy deployment in europe, Energy 206 (2020) 118060.

[50] Renewables 2020: Analysis and forecast to 2025, accessed 5 July 2025 (2020).
URL https://www.iea.org/reports/renewables-2020

[51] B. Odgerel, L. Pugliaresi, M. Lynch, A critical assessment of the iea's net zero scenario, esg, and the cessation of investment in new oil and gas fields, Tech. rep., Energy Policy Research Foundation, Inc. (EPRINC), Washington, D.C., accessed 5 July 2025 (Jun. 2023).
URL https://www.eprinc.org

[52] J. Norbeck, T. Latimer, C. Gradl, S. Agarwal, S. Dadi, E. Eddy, S. Fercho, C. Lang, E. McConville, A. Titov, K. Voller, M. Woitt, A review of drilling, completion, and stimulation of a horizontal geothermal well system in north-central nevada, in: Proceedings of the 48th Workshop on Geothermal Reservoir Engineering (SGP–TR–224), Stanford University, Stanford, California, 2023, workshop held 6–8 February 2023.

[53] J. H. Norbeck, T. M. Latimer, Commercial-scale demonstration of a first-of-a-kind enhanced geothermal system, EarthArXiv preprint, non-peer-reviewed manuscript (2025).
URL ⟨addEarthArXivlinkorDOIhere⟩

[54] K. England, P. Li, P. Xing, J. Moore, J. McLennan, 2024 enhanced geothermal system hydraulic fracturing campaign at utah forge, in: Proceedings of the SPE Hydraulic Fracturing Technology Conference and Exhibition, no. SPE-223519-MS, Society of Petroleum Engineers, The Woodlands, Texas, USA, 2025, conference held 4–6 February 2025. doi:10.2118/223519-MS.

[55] Danish Energy Agency, Energinet, Technology Data, fourth revised edition Edition, Danish Energy Agency, Copenhagen, Denmark, 2024.

Author Response to Reviews of

Market Integration Pathways for Enhanced Geothermal Systems in Europe

Lukas Franken, Elisabeth Zeyen, Orestis Angelidis, Tom Brown, Daniel Friedrich
Nature Communications
Revision #2: December 20, 2025

RC X.Y: Reviewer Comment, **AR:** Author Response, ▎*Manuscript Text*

General Reply

We thank the reviewers for their thoughtful and insightful comments, and hope to have addressed the concerns adequately.

Reviewer 1

AR: We thank Reviewer 1 for the detailed and constructive feedback, and agree that the points raised improve the quality of the work. In response, we have added a sensitivity study, two figures, and various discussions and text amendments. We hope these changes address the reviewer's concerns satisfactorily. We believe a small subset of (sub-)points fall outside the scope of the present work; in those cases, we have added notes in the supplemental material.

RC 1.1 The manuscript now links drilling cost reductions to learning rates (∼20–25%) but still treats them somewhat simplistically. It remains unclear how robust these estimates are across different global deployment scenarios. A comparison to other energy technologies' historical learning curves (wind, solar, shale gas drilling) would strengthen the case.

AR: We agree that additional context would enrich the discussion, and have therefore expanded the manuscript to better situate our assumed learning rates relative to historical learning rates observed for other energy technologies. In addition, we have added a sentence that estimates how the implied learning rate would change under the sensitivities evaluated in our analysis, to make the robustness (and limits) of the assumption clearer.

> ***[Added to Results: "Heat Generating EGS as Market Entry"]*** *For context, historical learning-rate estimates are commonly reported at roughly 20–30% for solar PV, 10–20% for onshore wind, and 10–15% for shale gas drilling (per doubling of cumulative deployment) [1, 2]. Therefore, the learning rates estimated here, 20–25%, appear high but plausible.*

[Added to Discussion: "Two Phases of EGS Deployment"] *Note that this is distinct from the in this paper estimated technology learning rate required for EGS roll-out to be self-sustaining[a] of 20–25%—a much steeper requirement than assumed in the input data (of 7.5 %) [3], but comparable, for instance, to reported solar PV learning rates (20–30% [1]). Technology-learning spillovers from outside Europe would reduce the value estimated here; conversely, as shown in the Sensitivities, less stringent emissions constraints in Europe or a business-as-usual district heating roll-out could significantly increase it.*

[a]Market opportunity at one level of cost reduction results in the technology learning that unlocks the next level of cost reduction.

RC 1.2 The sensitivity test of transmission capacity (+12.5% / +25%) is acknowledged, but the justification for using only modest increments is thin. Given that long-distance transport of EGS electricity is critical for Central European concentration, more rigorous treatment (or discussion of modeling limits) is needed.

AR: After review, we agree that an expansion scenario of +25% relative to today's transmission capacity is modest compared with plausible long-run expansion levels. We have therefore added an additional +50% sensitivity. Under this scenario, overall system costs decrease, but EGS build-out is lower than in the +25% expansion case. A likely explanation is that other renewable technologies benefit equally from improved transmission access, enabling better siting and (slightly) reducing the relative advantage of EGS. We have folded this rather interesting finding into the text and Fig 7.

[Added to Results: "Higher Renewable Costs and Larger Roll-Out of District Heating Maximize Market Opportunity"; and expanded sensitivity figure] *We further assume that past trends of onshore grid reinforcement lagging behind renewable roll-out will continue, and therefore examine sensitivities with an additional 12.5 %, 25 %, and 50 % grid capacity (expressed in GWkm), in line with expectations for the magnitude of future changes [4]. This addition results in a 1–3 % capacity increase for electricity-generating EGS and a 5 % decrease for combined-heat-and-power EGS, leading us to conclude that our findings are largely robust to future transmission-capacity expansion, also under a +50% expansion; in that case, EGS build-out even slightly decreases relative to a +25% expansion, likely because other renewables similarly profit from improved siting enabled by higher transmission capacity.*

[(Only changes in middle sentence) Added to Discussion: "Risks and Opportunities During EGS Rollout"] *Due to the difficulty of transporting heat, EGS cannot relocate to take advantage of the spatial flexibility offered by larger transmission capacity. As such, the common association of transmission expansion benefiting renewable energy holds for EGS only to a very limited extent, as other renewables can typically also exploit the additional spatial flexibility.*

RC 1.3 Figures of system cost reductions show system savings $< 1\%$–6%. While acknowledged,

the authors should clarify whether these differences are within model uncertainty bands. Otherwise, the significance of such small changes is questionable.

AR: We agree that uncertainty in system cost changes warrants a more nuanced treatment and split that discussion into numerical and assumption-related/parametric uncertainty.

Numerical noise is likely to play a negligible role in the findings. PyPSA-Eur solves to standard solver feasibility/optimality tolerances of order 10^{-6} (relative scale), i.e. differences in the objective value attributable to solver inaccuracy are on the order of 10^{-6}. This is orders of magnitude smaller than the reported 1%–6% cost changes [5].

Second, there is assumption-related and parametric uncertainty (techno-economic inputs, spatial/temporal aggregation, omitted constraints, etc.) which is likely of the same order of magnitude as the reported findings in large-scale, long-horizon energy system optimisation models. For this reason, we present cost impacts also in relative terms (percent of total system cost), which are more robust than absolute cost levels, and we further provide sensitivity analyses to assess robustness against perturbations.

To clarify this context, we have added a brief discussion to the Limitations section.

> ***[Added to Methods: "Limitations"]*** *Large-scale optimisation models are inherently subject to two kinds of noise: numerical optimisation imprecision and uncertainty arising from modelling assumptions and parameter choices. Numerical noise is negligible here, as PyPSA-Eur is solved to standard feasibility/optimality tolerances of order* 10^{-6} *(relative), far below the magnitude of the reported* $< 1\%$*–6% system-cost changes [5]. By contrast, parametric and structural uncertainty (e.g. techno-economic inputs, spatial/temporal aggregation, and omitted constraints) may be comparable to a few percent in long-horizon energy system optimisation. We therefore interpret the reported savings as conditional comparative results within a consistent modelling set-up, and complement them with sensitivity analyses to assess robustness to perturbations.*

RC 1.4 The manuscript briefly notes China/Russia industrial heat opportunities, but the discussion remains superficial. More depth on how European learning could transfer globally would improve the framing.

AR: We thank the reviewer for this suggestion and agree that the discussion of global market opportunity and knowledge transfer can be strengthened. We have expanded the Discussion in two ways. First, we note that publicly accessible geospatial platforms are increasingly providing the data layers required for first-pass screening of geothermal heat opportunities, including overlays of subsurface favourability with proxies for heat demand and district-heating potential (e.g., GeoMap) [6]. Using these layers as a qualitative check, we observe that large district-heating regions outside Europe (including parts of Eurasia) appear to coincide with areas of high geothermal favourability, suggesting that the market dynamics highlighted here could extend beyond the European case. However, we emphasise that such map-based screening does not substitute for a spatially explicit techno-economic market simulation that endogenously represents competition

with alternative heat-supply options under network and operational constraints; conducting such an analysis at global scale remains an important direction for future work. Second, we clarify that cross-regional knowledge spillovers appear plausible because a substantial share of advances in EGS-related drilling and subsurface engineering is disseminated through the open scientific and engineering literature, while the remaining know-how embodied in specialised equipment and services could diffuse through internationally operating supply chains [7]. A deeper empirical assessment of the rate and extent of these spillovers is beyond the scope of this study, and we therefore frame our remarks as qualitative.

> ***[Added to Discussion: "Spatial Distribution of EGS Adoption"]*** *The results have further implications beyond Europe, where district- and low-temperature industrial heat demands are substantially larger. For instance, China and Russia each exceed 4,500 TWh of annual district heating demand, while the rest of the world accounts for more than 1,500 TWh [8]. Importantly, the geospatial data layers needed to extend such assessments beyond Europe are increasingly available via publicly accessible geothermal mapping platforms (e.g., GeoMap), which combine subsurface favourability with surface-relevant layers for heating and industrial applications [6]. However, a comprehensive global analysis that explicitly simulates techno-economic competition between geothermal and alternative heat-supply options under network and operational constraints remains to be carried out. Adoption of EGS on a meaningful scale in these regions could boost European adoption since cross-regional knowledge spillovers appear plausible: many advances relevant to next-generation geothermal (e.g., drilling and subsurface engineering methods) are disseminated through the open scientific literature, while additional know-how embodied in specialised equipment and services can diffuse through internationally operating supply chains [7].*

RC 1.5 Although mitigation strategies of induced seismicity are cited, the risks of field implementation remain understated. The authors should balance optimism with clear acknowledgment of uncertainties.

AR: We agree and have strengthened the framing to make clear that induced seismicity can be a binding constraint that is not captured endogenously in our modelling framework; accordingly, our quantified results should be interpreted as conditional on social and regulatory feasibility, and have made the respective changes in the text

> ***[Expanded in Discussion: "Risk and Opportunities During EGS rollout"]*** *However, despite these measures, a non-zero probability of an EGS-induced seismic event—detrimental to EGS' public perception—will remain and, similar to excessive regulatory hurdles, cast additional uncertainty over the technology's future.* ~~*These requirements can lead to delays and additional costs that are difficult to capture in the present modelling and occur against the backdrop of a continuing risk that any serious future incident could render EGS politically untenable.*~~

RC 1.6 Regarding the PyPSA model, the paper does not show any benchmarking of the modified

EGS module against existing field data or alternative models. Even a qualitative comparison would improve confidence.

AR: We agree that the manuscript should provide more context by comparing our EGS cost assumptions to other sources, even if only qualitatively. To fix this, we have added text that compares the LCOEs used in our model, as shown in Figure A.22, to cost estimates from related work.

One relevant point of comparison is the recent IEA / Project InnerSpace report *The Future of Geothermal Energy* [9]. Under an optimistic cost trajectory, that report suggests that EGS could reach LCOEs as low as 50 USD/MWh by 2035 and 35 USD/MWh by 2050. However, these values are presented as global benchmarks rather than Europe-specific estimates, so a direct one-to-one comparison is not possible.

Nevertheless, the reported values are markedly lower than the majority of the LCOEs in our assessment (Figure A.22): only a small minority of modelled cost points—even under the strongest cost-reduction assumptions considered—reach below 50 EUR/MWh. A likely reason is that the cost-reduction trajectories in our analysis, taken from [3], correspond to a technology learning rate of 7.5%, which is lower than learning rates assumed in parts of the more recent literature (e.g. [10]). We therefore agree that the maximum cost reductions explored here are somewhat more conservative than some optimistic benchmarks; however, given the substantial uncertainty around future EGS costs, we consider the higher-cost tranches that drive most of our results to remain within the range assessed across the broader literature, and further emphasise that our main findings rather relate to the "tipping points" where EGS' market opportunity greatly expands and only after that concern the hypothetical market opportunity under extreme and highly uncertain cost reductions.

> ***[Added to Discussion: "Two phases of EGS-deployement"]*** *A qualitative comparison to alternative cost estimates suggests that our trajectories are conservative relative to some optimistic benchmarks. For example, a recent IEA report [9] suggests that, under an optimistic trajectory, EGS could reach LCOEs as low as 50 USD/MWh by 2035 and 35 USD/MWh by 2050. While these values are global and are therefore not directly comparable to our Europe-focused results, they lie below most of the LCOEs assessed in the present study (see Figure A.22). For context, the bespoke market expansion case with a 60% cost reduction for electricity-generating EGS corresponds to LCOEs of roughly 75–120 €/MWh. The relatively modest learning rate (7.5 % per capacity doubling) underpinning our input data and therefore our assessed cost reductions [3] is likely responsible for this difference.*

RC 1.7 The flexible-operation scenario is mentioned, but how the temporal resolution (hourly/daily) of the energy system model captures reservoir storage dynamics remains vague.

AR: We agree and have added a figure in the appendix and the respective discussion to show the geothermal reservoir's operational pattern when flexibility is enabled. Both seasonal and intra-day arbitrage are found, similar to Fig. 7 in Ricks et al. 2025 [11]. This suggests that the 3-hourly resolution of the model captures both seasonal and intra-day arbitrage opportunities

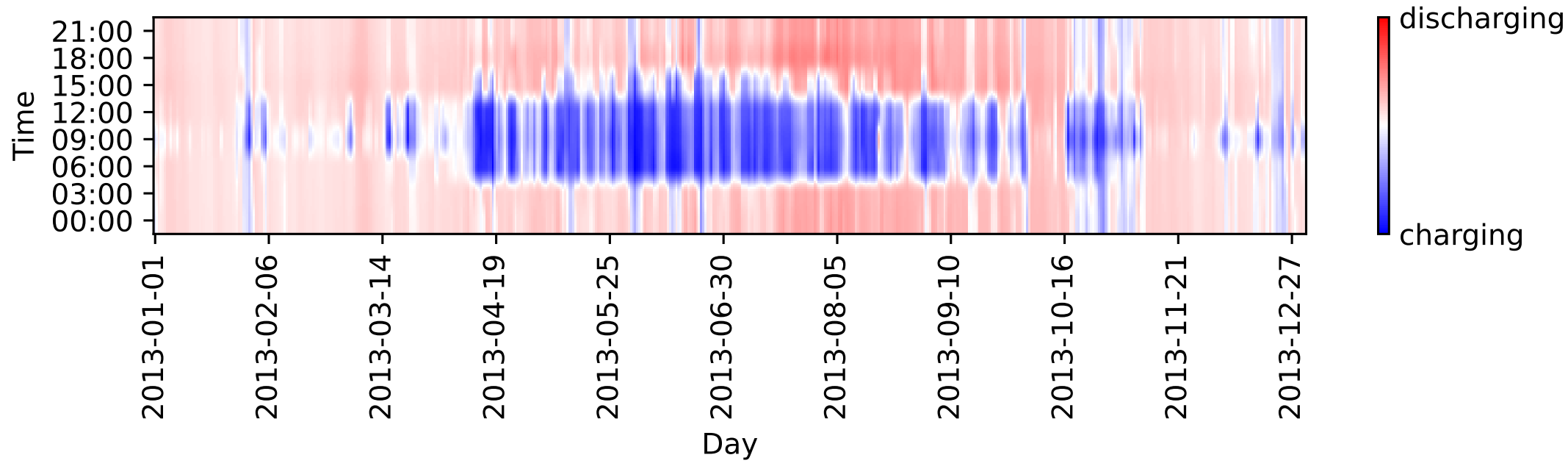

Figure 1: Operational pattern of flexible EGS, visualised as an average across network regions for electricity-generating EGS at 30% of today's cost. [*Added to the Appendix as Fig A.23*]

enabled by flexible operation.

> ***[Expanded in Results: "Higher Renewable Costs and Larger Roll-Out of District Heating Maximize Market Opportunity"*** *Operationally, we find that both seasonal and intra-day arbitrage contribute to EGS' market opportunity (Fig. 1), comparable to the behaviour found in [11]. Both effects are resolved by the model's 3-hour temporal resolution.*

RC 1.8 The displacement of water tanks by EGS at high penetration is intriguing but needs more mechanistic explanation. Why does the model prefer to eliminate short-term storage entirely instead of co-optimizing?

AR: We agree that this discussion could be treated in more depth. We also note that short-term storage is not eliminated entirely but only partially, indicating that co-optimisation is indeed occurring. The main dynamic is that lower marginal electricity costs lead the model to prioritise the electrified heating route over direct heating from boilers and solar thermal. We checked the data to confirm that the displaced hot-water storages are primarily used to balance the seasonal profile of solar thermal. Accordingly, it is this seasonal balancing role that becomes less necessary under low-cost, EGS-enabled electrified heating, and is therefore displaced by the model. We have added some text to the paper to discuss this nuance.

> ***[Added to Results: "Heat Generating EGS as Market Entry"]*** *The thermal storage that the model displaces along with solar thermal exhibits a seasonal charging pattern rather than an intra-day one. Therefore, it is primarily seasonal balancing that becomes less valuable as dispatchable electrified heating, enabled by EGS, expands.*

RC 1.9 The back-of-the-envelope calculation on drilling rigs is helpful but simplistic. A more nuanced treatment (e.g., learning in drilling speed, supply-chain bottlenecks) would strengthen the argument.

AR: We agree the drilling-rig calculation deserves a more nuanced treatment. Upon review, we

also found that only a subset of Europe's active drilling fleet is practically suitable and available for deep onshore geothermal drilling; stakeholder evidence suggests that the current pool of specialist geothermal-capable rigs is comparatively small (order 10^1 rigs across Europe, rather than ∼100 as assumed before) and that recent projects have had to source appropriate rigs (and associated crews/services) internationally [12]. This could constitute a meaningful bottleneck for rapid EGS scale-up and underscores the importance of technology, skills, and service-sector transfer from the oil-and-gas industry [12]. We have added corresponding clarifying text to the manuscript.

> ***[Expanded in Discussion: "Two Phases of EGS Deployment"]*** *As a back-of-the-envelope calculation, one drilling rig with an average well-drilling time of 60 days could add about 30 MW_{th} per year (assuming one well doublet provides ∼10 MW_{th}). At that rate, developing 30 GW_{th} would demand roughly 1,000 rig-years, i.e. on the order of 100 years with 10 rigs. While Europe hosts around 120 active drilling rigs overall [13], stakeholder interviews suggest that only a small subset is suitable for deep onshore geothermal drilling; for example, a recent supply-chain consultation reported only ∼13 geothermal-capable rigs across Europe [12]. This underscores both the practical importance of rig and service-sector availability in any large-scale EGS roll-out and the critical role of technology and skill transfer from the oil-and-gas industry.*

RC 1.10 Regarding the economic metrics, only system cost reductions are reported. Metrics like LCOE distributions, payback time, or capacity factors would make the results more actionable for policymakers.

AR: We agree that reporting additional economic metrics would enrich the analysis and improve its policy relevance. To provide a more actionable and intuitive perspective beyond aggregate system-cost changes, we added Fig. 2, which shows marginal price (reductions) as a function of EGS cost reduction across both electricity and district-heating nodes. In addition, we now more explicitly highlight Fig. A.22, which summarises the distribution of LCOEs in the underlying geothermal resource data. Accordingly, we added a few sentences in the main text that refer to these figures and clarify how they complement the system-cost results.

> ***[Added to Results: "Heat-Generating EGS as Market Entry"]*** *As EGS penetration increases, marginal energy prices at both electricity and district-heating nodes decrease. For power, the system-wide load-weighted average electricity price is around 88 €/MWh without EGS and, consistent with the reduction in system costs, falls to 70 €/MWh under the strongest EGS cost reductions. District-heating prices start at around 37 €/MWh and decline to roughly 20 €/MWh for medium cost reductions and under extreme EGS cost reductions to about 10 €/MWh (Fig. 2).*

RC 1.11 The manuscript mentions EU directives, but lacks discussion of how national-level permitting, social acceptance, or induced-seismicity regulation might slow rollout.

AR: We agree that some additional information regarding national regulation would enrich the

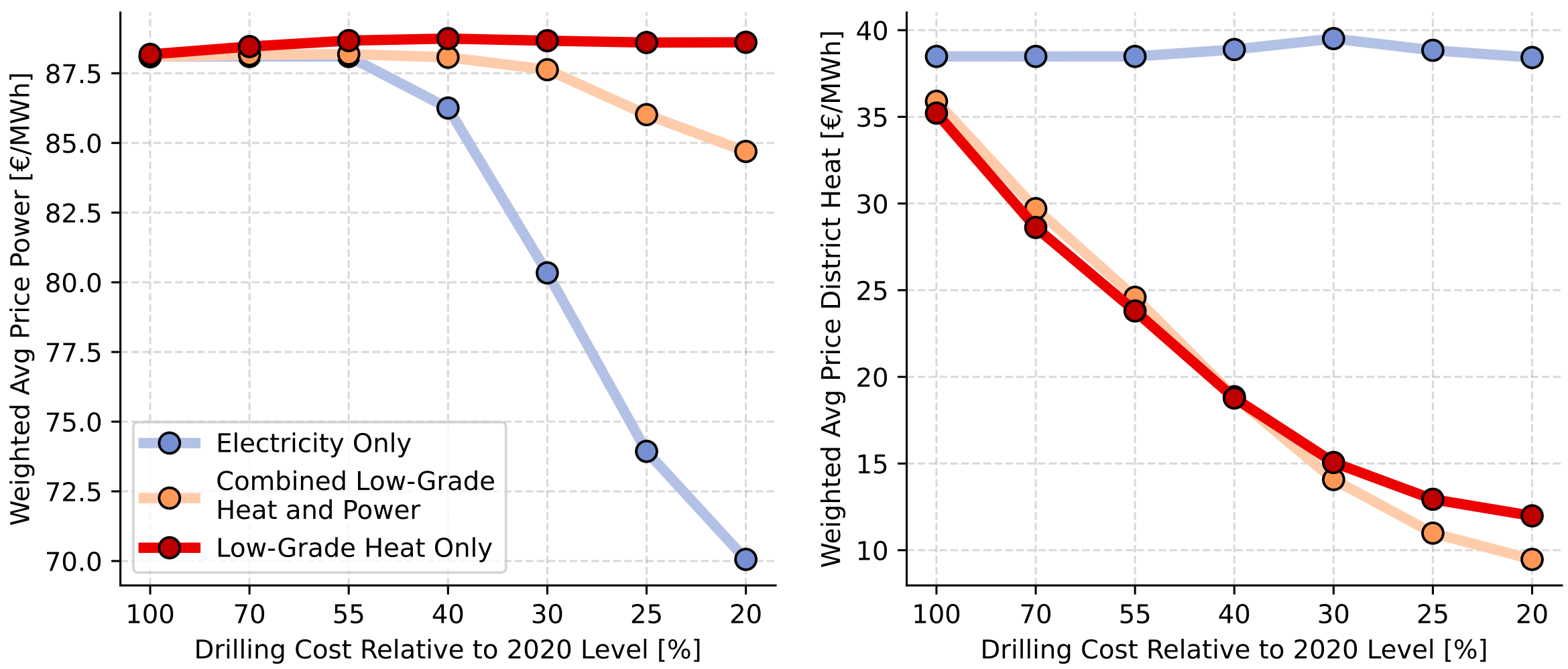

Figure 2: Load-weighted average energy prices in electricity and district heating nodes for different levels of EGS cost reductions.

text, but we are also hesitant to add too many details because the volume of content can bloat out of control. The additions we made to the Discussion therefore focus on four regulatory / public-perception topics:

- **Heat-market access:** There are national examples for third-party access (TPA) and related rules in district-heating–heavy countries such as **Sweden** and, more broadly, in several **Eastern and Baltic** member states.
- **De-risking:** explicit public or public–private mechanisms to socialise geological/drilling risk exist in a small set of large heat markets, notably **France**, the **Netherlands**, **Germany** and **Poland**. A broader review suggests that dedicated geothermal risk-mitigation schemes remain limited to a handful of European countries overall.
- **Induced-seismicity regulation:** guidance and/or operational protocols are documented for **France**, **Switzerland**, and the **Netherlands**, while in **Germany** such requirements are commonly incorporated via permitting practice rather than a single national framework. In the **United Kingdom**, reviews emphasise that governance is often treated in a permit-specific manner.
- **Public perception:** It appears that public acceptance is largely linked to perceived induced-seismicity risk, therefore the national perspective is less interesting.

We have not added distinct new paragraphs to the Discussion; instead, where appropriate, we inserted short national-level context into the existing text.

> ***[Added to Discussion: "Two phases of EGS deployment"] [With regard to heat market access]*** *In addition, national policies already exist in some district-heating–heavy countries—for example, Sweden's District Heating Act includes conditional third-party access (TPA) for injecting heat into networks, and cross-country reviews document substantial heterogeneity in analogous access rules across other EU member states [14, 15].*

*[**Added to Discussion: "Two phases of EGS deployment"] [With regard to de-risking]** Such policies are increasingly being implemented across European countries, including several large heat markets such as France, the Netherlands, Poland and Germany [16, 17, 18, 19, 20]. These incentives could serve as a powerful lever to break the "investment trap", whereby high geothermal costs hinder widespread adoption and limited adoption in turn slows technology learning.*

*[**Added to Discussion: "Risks and Opportunities During EGS Rollout"] [With regard to induced-seismicity regulation]** Across Europe, anti-seismicity protocols span national risk-screening frameworks (e.g., Switzerland), permit-embedded traffic-light systems (e.g., France and the Netherlands), and industry guidance that informs case-by-case permitting (e.g., Germany); by contrast, in the UK induced-seismicity management has largely been handled through local planning conditions only [21, 22, 23, 24, 25, 26].*

RC 1.12 The role of advanced heat pumps, CCS with waste heat, or nuclear district heating as competitors is underexplored.

AR: We agree with the reviewer that a deeper comparison of geothermal district heating against alternative low-carbon heat options would be valuable, but a comprehensive treatment is beyond the scope of this study; we therefore added a brief discussion of these competitors in the Limitations.

Regarding heat pumps, our district-heating representation already includes electrified supply options (heat pumps and resistive heating); if the reviewer refers to *advanced* (e.g., high-temperature or next-generation) heat-pump configurations, their performance and cost trajectories remain highly uncertain and are not explicitly parameterised here. Similarly, CCS with waste heat depends on uncertain future capture rates, transport and storage costs, utilisation of low-grade heat, and integration constraints; introducing this additional layer of uncertainty alongside geothermal learning would materially expand the modelling scope. Some illustrative context can be derived from a LCOE comparison between the EGS potentials used in this paper and plausible future CCS cost outcomes (for the power sector) (that figure had already been included). The figure shows overlapping cost ranges and hence that the relative competitiveness remains uncertain. Finally, nuclear power and by extension its waste heat utilisation are subject to political decisions and siting constraints which complicates an adequate treatment in an economic model. Further, the limited number of nuclear sites in Europe, combined with the spatial constraints of heat distribution, suggests that its contribution to district heating is likely to remain comparatively small.

*[**Expanded in Discussion: "Limitations"]** Our analysis does not model waste-heat supply from CCS-equipped thermal plants or from nuclear facilities as competing heat sources for district heating. The future roll-out and cost of both of these technologies are subject to large uncertainties, and therefore their integration into the model under blanket cost assumptions is confounding rather than insightful. To illustrate this, we have included an illustrative LCOE-*

> *based comparison for gas power plants with CCS (Fig. A.22), highlighting the overlap in their uncertainty bands due to both investment and fuel costs. For nuclear, the potential for district heating is strongly constrained by siting and political feasibility, both of which are difficult to capture in a purely economic model. However, both technologies constitute realistic alternatives to geothermal district heating, and future work could provide an adequate treatment of their potential future competition.*

RC 1.13 The response argues that early heat-based rollout can unlock electricity competitiveness, but the manuscript lacks a robust quantitative link (e.g., explicit scaling of cost reduction with installed heat-only capacity).

AR: We believe the reviewer asks for a causal chain from early heat deployment to later electricity competitiveness that is explicit and quantitative. Our manuscript includes a figure that estimates the learning rate required for progressively increasing EGS deployment to become self-sustaining as a function of drilling cost. The learning rate is estimated by relating the market opportunity of neighbouring cost steps (see Methods *Technology Learning Rate*). Roughly, we find that a technology learning rate of 20% would be sufficient to generate self-sustaining growth (see Figure A.9). However, upon review, we find that the only link in the text to this figure and result is in one paragraph in the Results. We have added another reference in the Discussion subsection *Two phases of EGS deployment*, and hope this clarifies the issue.

> ***[Expanded in Discussion: "Two phases of EGS deployment"]*** *Based on this, we estimate that a technology learning rate of around 20% during this phase (Figure A.9) could lead to self-sustaining growth into a second phase.*

Reviewer 2

We thank the reviewer for his comment. It is a very central point, and upon review we agree it deserves to be addressed thoroughly.

RC 2.1 I have problems with the paper. I'm not an expert in economic modelling. I have seen that the other reviewers are. I consider me as a technology expert. Therefore, I made comments about missing technology aspects. In the new version, they we're mentioned. However, the answer of the authors was in addition, that these developments are part of the learning curve and they led to cost reduction without going into details how this should function. I know that these new developments have the consequence of deeper drilling but lower seismic problems. That means either increase by deeper drilling or decrease by less seismic events. Therefore, cost reduction may occur but based on a different cost compilation. I wonder if the used cost models can do this. Nevertheless, these influences on the cost models were not presented in the revised paper.

AR: We thank the reviewer for highlighting that our revised manuscript did not sufficiently explain (i) how the assumed cost reductions could arise and (ii) whether they are applied to individual technology components or only in aggregate. After review, we agree that the manuscript

was lacking important details on this central methodological aspect of the analysis. To address this shortfall, we have made what we hope are satisfactory additions to both the Methods and the Limitations.

In particular, we now explain in the Methods how cost reductions in the input data from [3] are constructed, and how that translates into our modelling. We added around three to four paragraphs to fill the gap (see below). In short, we clarify that, consistent with [3] (where the learning factor multiplies aggregated CAPEX; Supplementary Eq. S1), cost reductions in our analysis are applied to cost *aggregates* rather than to individual sub-components. Therefore, our model does not endogenously reallocate costs between components (e.g. higher depth-driven drilling costs versus lower induced-seismicity mitigation, etc.); such effects can only be represented insofar as they change the *net* project cost captured by the applied reduction factor.

Further, we have added a dedicated Limitations paragraph to make this reduced-form treatment explicit and to aid interpretation. Our results should be read as thresholds for *net* cost reductions required for competitiveness in power and heat markets, which stakeholders may then map to specific technological pathways via their implied net impact on well-field and/or surface-plant costs. To improve coherence, we also moved the existing Methods section *Technology Learning* behind *Modelling Enhanced Geothermal Systems* and added bridging text to clarify how it relates to the preceding discussion.

To address the reviewer's concern, we have made the following concrete changes and additions:

1) Added discussion in the Methods section of how exactly the cost reductions are implemented, and how they are applied to cost aggregates rather than components.

2) Folded the Methods on the spatial distribution of EGS techno-economic data into the general discussion of how we model EGS (Methods section *Modelling Enhanced Geothermal Systems*). The structure should now be more coherent, moving from the input data, to the cost reduction assumptions, and only then to how these are implemented in our model.

3) Added a paragraph in the Limitations highlighting that cost reductions are not applied to components of total costs, and suggesting how, in light of this, stakeholders can derive value from our findings.

4) Moved the existing Methods section *Technology Learning* behind the aforementioned section *Modelling Enhanced Geothermal Systems* and added a paragraph to clarify how it relates to that section.

> ***[Added to Methods: "Modelling Enhanced Geothermal Systems" (the first paragraph is not fully new, and instead features parts of a paragraph from the previous revision)]*** *We begin with a discussion of the data inserted, independent of the modelling framework, and only then move to its representation in PyPSA-Eur.*
>
> *We use techno-economic data from [3] to represent cost and the spatial aspects of geological suitability in the model and refer to that paper for a more elaborate methodological description on how the numbers are derived, but provide a brief overview here. Building on the protocol proposed in [27], the* $1^{\circ}\times1^{\circ}$ *gridded data provided in [3] estimates, for each square and based*

on geological data-maps, the cost-optimal EGS depth by optimising the trade-off between higher costs through deeper drilling and lower surface plant costs achieved by higher temperatures of geological resources.

Further, [3] makes assumptions about future cost reductions resulting from technology learning. Based on the projected exponential increase in EGS capacity towards 2050, paired with a technology learning rate of 7.5% at each doubling of built capacity, the paper asserts the cost reductions used in the present paper (see Figure 1, reaching a maximum cost reduction factor of 5 in 2050). To make our results more intuitive, we decouple the cost reduction from the specific year assigned in [3] and instead express it as a factor relative to costs estimated for 2020. While [3] is supplemented only by country-level data, courtesy of the author, we were able to access the underlying gridded data for our experiments. Each region in our model was assigned an EGS potential based on the overlay with its geometry.

The technology-learning assumption in [3] is implemented as a blanket *cost reduction of the* total *EGS capital cost, rather than as separate learning rates for individual sub-components, and that assumption is inherited into the present paper. In particular, the learning factor is multiplied with the aggregated CAPEX (see Supplemental Material Eq. S1 in [3]), which sums well drilling & completion costs and surface power-plant costs (i.e. the ORC plant), alongside other project components. Neither [3] nor our implementation models seismicity-related costs endogenously; such effects can only be represented via an assumed net CAPEX adjustment.*

Analogously, the present paper translates the learning assumption into an aggregated representation: we scale the full EGS cost pipeline, i.e. drilling and the ORC surface plant, by a single cost-reduction factor relative to the 2020 baseline. The results therefore represent net *project cost reductions irrespective of which specific technological advances (e.g. improvements in drilling equipment, reduced seismicity-related costs etc.) ultimately deliver them. Policy risk-sharing could change the effective private cost baseline; we note this in Limitations.*

[Added to Methods: "Technology Learning"] *The techno-economic inputs in our work are taken from [3]. There, the authors exogenously assume an (exponentially) increasing future roll-out along with a learning rate, and infer the resulting cost reduction that scenario would see. The resulting cost levels are what we use here. Our work in contrast, finds EGS roll-out endogenously as a function of exogenously imposed cost reductions. Therefore, the inserted cost-reduction levels are no longer tied to the technology learning mechanism in [3]. Instead, we merely inherit the range of cost reductions deemed plausible by that paper. Given the pair* (cost reduction, deployment)*, we then infer the learning rate required for deployment to be self-sustaining. This implied learning rate is shown in Fig. A.9, and this section provides the context for that figure.*

[Added to Methods: "Limitations"] *Our cost-reduction scenarios are implemented as a multiplicative factor on aggregated EGS CAPEX (well-field/drilling and, where applicable, the ORC surface plant), following [3] where learning is applied to total CAPEX (Supplementary*

> *Eq. S1). Consequently, neither [3] nor our implementation endogenously allocates costs between individual mechanisms, such as higher drilling expenditures from deeper wells versus lower expenditures associated with induced-seismicity mitigation; these effects can only be reflected insofar as they change the* total *project CAPEX captured by the applied factor. Moreover, local regulation could change the effective project cost and thereby complicate comparison to this work. This could for instance apply in case of partial public coverage of unsuccessful exploratory drilling, as implemented in parts of Germany [28]. If this is the case, the relevant comparison to our thresholds is against the developer's expected, policy-adjusted cost.*

References

[1] U.S. Department of Energy, Office of Energy Efficiency and Renewable Energy, Wind Energy Technologies Office, Learning a better way to forecast wind and solar energy costs, blog post (Oct. 2022).
URL https://www.energy.gov/eere/wind/articles/learning-better-way-forecast-wind-and-solar-energy-costs

[2] R. Fukui, et al., Experience curve for natural gas production by hydraulic fracturing, Energy PolicyReports an experience-curve learning rate for shale gas production (2017).

[3] A. Aghahosseini, C. Breyer, From hot rock to useful energy: A global estimate of enhanced geothermal systems potential, Applied Energy 279 (2020) 115769.

[4] S. Butorac, Eu electricity grids, Briefing PE 772.854, European Parliamentary Research Service, accessed 25 June 2025 (May 2025).
URL
https://www.europarl.europa.eu/RegData/etudes/BRIE/2025/772854/EPRS_BRI(2025)772854_EN.pdf

[5] J. Hörsch, F. Hofmann, D. Schlachtberger, T. Brown, PyPSA-Eur: An open optimisation model of the european transmission system, arXiv preprint (2018). arXiv:1806.01613.
URL https://arxiv.org/abs/1806.01613

[6] Project InnerSpace, GeoMap Press Release, PDF, accessed 2025-12-15 (Nov. 2025).
URL https://projectinnerspace.org/news/GeoMap_Europe_Press_Release.pdf

[7] International Energy Agency (IEA), The future of geothermal energy, Tech. rep., International Energy Agency, accessed 2025-12-15 (Dec. 2024).
URL https:
//iea.blob.core.windows.net/assets/cbe6ad3a-eb3e-463f-8b2a-5d1fa4ce39bf/TheFutureofGeothermal.pdf

[8] Renewables 2020: Analysis and forecast to 2025, accessed 5 July 2025 (2020).
URL https://www.iea.org/reports/renewables-2020

[9] International Energy Agency, The future of geothermal energy, executive summary. Published 13 December 2024. Licence: CC BY 4.0 (Dec. 2024).
URL https://www.iea.org/reports/the-future-of-geothermal-energy/executive-summary

[10] W. Ricks, J. D. Jenkins, Pathways to national-scale adoption of enhanced geothermal power through experience-driven cost reductions, Joule (2025).

[11] W. Ricks, K. Voller, G. Galban, J. H. Norbeck, J. D. Jenkins, The role of flexible geothermal power in decarbonized electricity systems, Nature Energy (2024) 1–13.

[12] C. Abesser, A. Gonzalez Quiros, J. Boddy, Evidence report supporting the deep geothermal energy white paper: The case for deep geothermal energy – unlocking investment at scale in the uk, Open Report OR/23/032, British Geological Survey, Nottingham, UK, publication type: Report (Unpublished in the NERC Open Research Archive record) (2023).
URL https://nora.nerc.ac.uk/id/eprint/535567/1/report_OR23032.pdf

[13] YCharts, European union rig count (i:eurr)Monthly rig-count time series; accessed 2025-07-10 (2025).
URL https://ycharts.com/indicators/european_union_rig_count

[14] E. Lundin, et al., Evidence from the swedish district heating sector, Tech. rep., Research Institute of Industrial Economics (IFN), working paper; please verify report number and URL in your library record. (2025).

[15] V. Bürger, et al., Third party access to district heating systems, Energy PolicyPlease verify full author list, volume, pages, and DOI against your library record. (2019).

[16] BRGM, Assessment of the deep geothermal energy sector for heat production since its revival in 2007 (renewable heat fund and geothermal guarantee fund), accessed 2025-12-29 (Sep. 2020).
URL https://www.brgm.fr/en/reference-completed-project/assessment-deep-geothermal-energy-sector-heat-production-period-2007

[17] Netherlands Oil and Gas Portal (NLOG), Rnes (warranty regulation): Guarantee scheme for geothermal energy, accessed 2025-12-29 (2025).
URL https://www.nlog.nl/en/rnes-warranty-regulation

[18] Munich Re, Bundeswirtschaftsministerium, munich re und kfw starten innovatives programm zur förderung der tiefengeothermie (absicherung des fündigkeitsrisikos), press release (18 Dec 2025); accessed 2025-12-29 (Dec. 2025).
URL https://www.munichre.com/de/unternehmen/media-relations/medieninformationen-und-unternehmensnachrichten/unternehmensnachrichten/2025/neue-absicherung-des-fuendigkeitsrisikos-fuer-geothermie-betreiber.html

[19] KfW, Neue förderung von tiefengeothermieanlagen zur kommunalen und industriellen wärmeversorgung (kfw-förderkredit geothermie), press release (18 Dec 2025); accessed 2025-12-29 (Dec. 2025).
URL
https://www.kfw.de/%C3%9Cber-die-KfW/Newsroom/Aktuelles/Pressemitteilungen-Details_875520.html

[20] Ministerstwo Klimatu i Środowiska (Poland), Finansowanie geotermii, accessed 2025-12-29 (2020).
URL https://www.gov.pl/web/klimat/finansowanie-geotermii

[21] T. Kraft, P. Roth, S. Wiemer, Good practice guide for managing induced seismicity in deep geothermal energy projects in switzerland, Report, Swiss Seismological Service (SED), ETH Zurich (10 2020).
URL https://www.research-collection.ethz.ch/handle/20.500.11850/453228

[22] J. Schmittbuhl, S. Lambotte, O. Lengliné, M. Grunberg, H. Jund, J. Vergne, F. Cornet, C. Doubre, F. Masson, Induced and triggered seismicity below the city of strasbourg, france from november 2019

to january 2021, Comptes Rendus. Géoscience 353 (2021) 561–584. doi:10.5802/crgeos.71.
URL https://comptes-rendus.academie-sciences.fr/geoscience/articles/10.5802/crgeos.71/

[23] L. Buijze, L. van Bijsterveldt, H. Cremer, B. Paap, J. Veldkamp, B. Wassing, J. van Wees, J. ter Heege, Review of worldwide geothermal project mechanisms and occurrence of induced seismicity, Tech. Rep. TNO-2019-R10043, TNO, Utrecht (2019).
URL https://repository.tno.nl/SingleDoc?docId=49466

[24] R. Schultz, A. Muntendam-Bos, W. Zhou, G. C. Beroza, W. L. Ellsworth, Induced seismicity red-light thresholds for enhanced geothermal prospects in the netherlands, Geothermics 106 (2022) 102580. doi:10.1016/j.geothermics.2022.102580.
URL https://www.sciencedirect.com/science/article/abs/pii/S0375650522002267

[25] Bundesverband Geothermie e.V. (BVG), Bvg-richtlinie 1101: Seismizität bei geothermieprojekten – teil 2: Reaktionsschema (ampel), Tech. rep., Bundesverband Geothermie e.V. (2023).
URL https://www.geothermie.de/fileadmin/user_upload/Bibliothek/Downloads/BVG_Richtlinie_Seismizitaet_bei_Geothermieprojekten_Teil2_Reaktionsschema__Ampel_.pdf

[26] GeoScience Ltd, Written evidence submitted by geoscience ltd (geo0031) to the uk parliament environmental audit committee inquiry on geothermal technologies, UK Parliament Committees (Written evidence), published 8 September 2022 (9 2022).
URL https://committees.parliament.uk/writtenevidence/110167/pdf/

[27] G. R. Beardsmore, L. Rybach, D. Blackwell, C. Baron, A protocol for estimating and mapping global egs potential, GRC Transactions 34 (2010) 301–312.

[28] Risikoabsicherung hydrothermale geothermie: Häufig gestellte fragen und antworten, Faq, NRW.BANK, fassung 05/25, Stand: 22.05.2025 (May 2025).
URL https://www.nrwbank.de/de/foerderung/dokumente/FAQ_Geothermie_05-25_II.pdf?contentType=application%2Fpdf&pfad=%2F0%2F5%2F10305%2F

Author Response to Reviews of

Market Integration Pathways for Enhanced Geothermal Systems in Europe

Lukas Franken, Elisabeth Zeyen, Orestis Angelidis, Tom Brown, Daniel Friedrich
Nature Communications
Revision #3: March 31, 2026

RC X.Y: Reviewer Comment, **AR:** Author Response, ▍ *Manuscript Text*

General Reply

We thank the reviewers for their comments, and are very happy to hear that we have, mostly, been able to address previous concerns. We also thank the reviewers for their continued support with the manuscript and the suggestions made. We have made the respective adjustments, mostly adding a figure and some context to the discussion and, hopefully, improving the reader's experience and access to the results.

Reviewer 1

General Response to Reviewer 1:

We thank the reviewer for the suggestions for the text, and agree they elevate the context and the overall quality of the work. We have done our best to adjust the content.

RC 1.1 You report a system-wide load-weighted average electricity price of ∼88 €/MWh (without EGS) decreasing to ∼70 €/MWh under strong EGS cost reductions. Please state explicitly (in the main text) that if these are model-implied prices from the optimized decarbonized system, or observed market prices, and specify the modeled year/scenario context when quoting these numbers.

AR: We agree there was residual ambivalence about this point in the text, and have added a sentence with a clarification to the main text.

> ***[Added to Results: "Heat Generating EGS as Market Entry"]*** *We note these are model-implied prices in a carbon-neutral system. They fall within the range reported in related literature (see for instance 2040 electricity price in [1]), but are subject to larger inter-study variation.*

RC 1.2 At present, the manuscript does not clearly benchmark EGS economics (LCOE / implied strike price) against current wholesale/retail electricity price ranges in Europe. Without this, readers may over-interpret the "market integration" framing as near-term feasibility. A short, well-cited sentence (or small table) contextualizing modeled prices relative to recent European market prices would materially strengthen the credibility and interpretation.

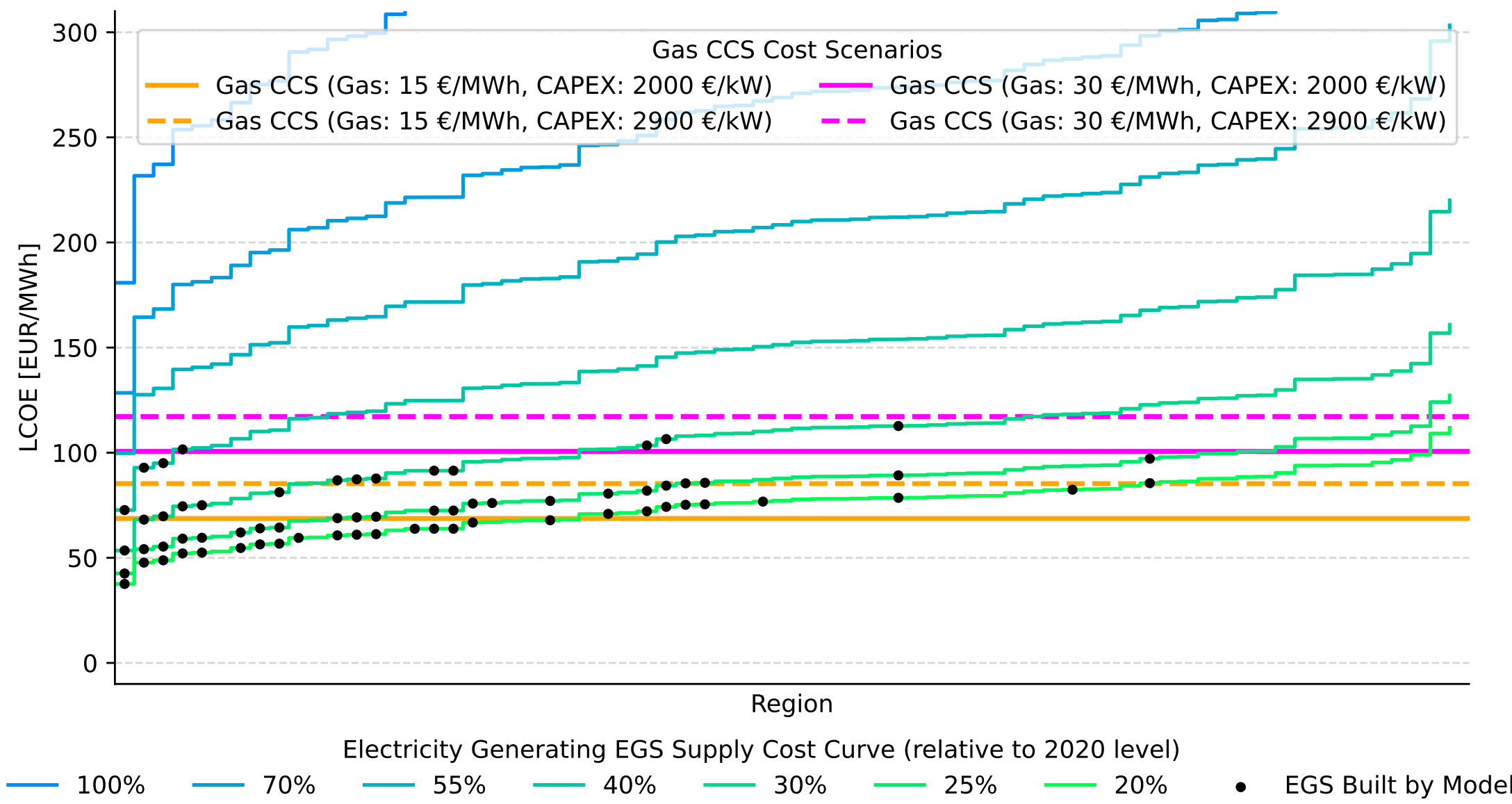

Figure 1: [Not a new figure, only added to this reviewer response for convenience.] Comparison of LCOE values from [3] used in the model to different projections for gas generators with attached carbon capture and storage. The gas generators are assumed to operate at an efficiency of 47% [5] and a capacity factor of 50%. [*Supplementary Figure*]

AR: We agree that a direct comparison between modelled and real prices would be a valuable addition towards putting the findings into perspective. We have added a passage to the Discussion that provides that context.

> ***[Added to Discussion: "Two Phases of EGS Deployment"]*** *The findings also align with a comparison between current European electricity prices[2] and the EGS LCOE used here from [3]. In 2020-2021, central European prices have been around 30-60 €/MWh, for which, to compete, EGS would need cost reductions more extreme than the scenarios assessed here (see Supplementary Figure 1). However, both the European near-complete embargo of Russian gas since 2022 (since then, electricity prices have settled between 50 and 150 €/MWh[2]) and carbon phase-out have led to higher electricity prices[1]. As of March 2026, the German baseload futures for 2027-2030 are in the range between 70 and 100 €/MWh[4]. As a result, the roughly 60% cost reduction, deemed required here, would likely hold in the real system, both in the gas-reliant current system and a future renewable one.*

RC 1.3 The rebuttal points to an LCOE distribution in supplementary, but the main manuscript still lacks a clear, reader-facing presentation of EGS LCOE ranges/distributions and how those relate to modeled prices. Please include (in the main text and/or a main figure) a concise summary of EGS LCOE distributions used/assumed and how they shift under your cost-reduction cases.

AR: We agree the LCOE range explored, regionally and through technology learning, needed to be expressed more clearly in the main text. To that end, we have enhanced the first figure. Its y-axis shows regional CAPEX value by regions, and now has been enhanced with a second

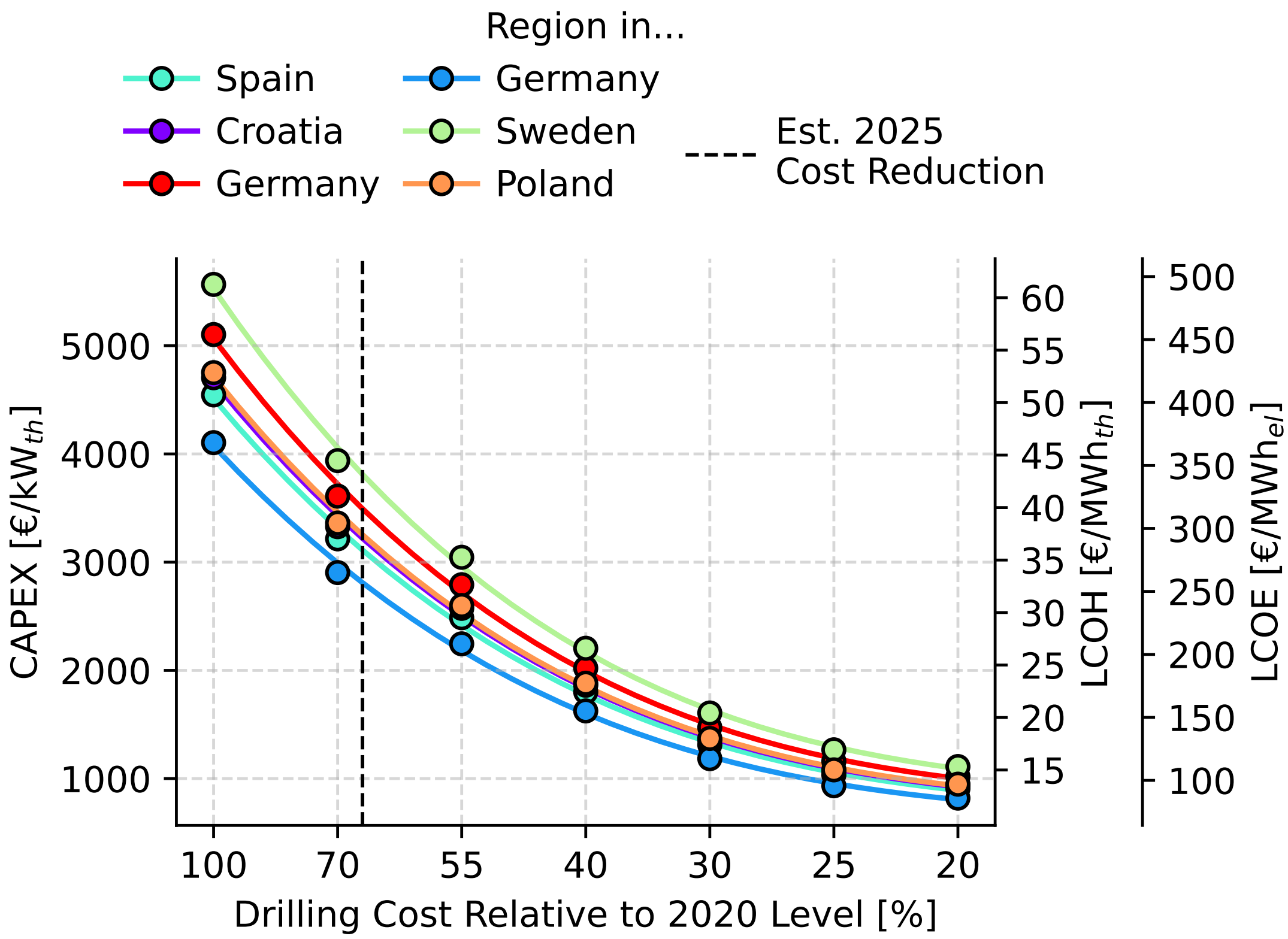

Figure 2: Scenarios of potential drilling cost reductions evaluated in the study, shown here for six out of 72 regions. All costs are assumed to decrease, however preserve the relative magnitude between regions, reflecting geological conditions. The scenarios cover the span of the cost reductions evaluated in [3]. The estimate of cost reductions achieved in 2025 (around 25%) is based on [6]. **(Rev 1; Com 3)** The LCOH and LCOE values are computed directly from the CAPEX assuming a capacity factor of 95%, for a detailed overview we refer to the Methods. [*Main text Figure 1*]

y-axis that maps those values to LCOE and LCOH. We hope this to be an efficient way of communicating the evaluated LCOE and LCOH ranges.

> ***[Added to Introduction: Figure 1 caption]*** *Note the CAPEX values on the left refer to heat, not power capacity, i.e. before application of the efficiency factor. The LCOH and LCOE values are computed directly from the CAPEX assuming a capacity factor of 95%, for a detailed overview we refer to the Methods.*

RC 1.4 The added marginal-price analysis is helpful, but it does not answer whether EGS projects are revenue-adequate under the modeled prices. Please add at least one of the following: (1) fraction of EGS sites with LCOE $\leq$ modeled node price, (2) implied required premium/subsidy (strike price – modeled price), or (3) net present value / payback indicators (even as distributions). This would directly address economic feasibility rather than inferring it indirectly from system-average prices.

AR: This is a good point, and highlights there was some remaining ambiguity about the setup of our study. It appears there was an impression that we forced EGS into the model, regardless of its economic competitiveness. Some related literature appears to be taking that route. In contrast however, the EGS capacities here are fully endogenous. In this framing, all deployed capacity is exactly revenue-adequate by construction. We have added a paragraph to the Methods to make this more explicit.

> ***[Added to Methods: "Energy System Model"]*** *Finally, the model in this work optimises, under constraints, investment decisions for all energy generation and conversion technologies, including EGS. This is in contrast to an alternative study design that could simulate varying technology deployments, and assess economic competitiveness through their effect on system cost. Therefore, the optimal deployment per technology in the present study is an endogenous variable, and all capacity that gets built is exactly revenue-adequate, i.e. profits are zero by design[7]. In this framing, the techno-economics of technologies offered to the model already account for the relevant incentives, most importantly for carbon-neutrality through the carbon budget constraint, and the respective level of EGS cost reductions.*

RC 1.5 One key request was capacity factor (or operating hours). The manuscript should report utilization/dispatch outcomes for EGS (e.g., median and interquartile range of annual generation or capacity factor by case). This is necessary to interpret costs and competitiveness, especially since EGS value depends on dispatchability and system need.

AR: We agree that the previous attempt at responding to this point fell short in showing only the charging and dispatch pattern of flexible EGS in one network. To give a broader perspective on the topic, we have added a discussion of capacity factors to the Results and two supplementary figures showing weighted average capacity factors across EGS modes and cost reduction scenarios.

> ***[Added to Results: "Higher Renewable Costs and Larger Roll-Out of District Heating Maximize Market Opportunity"]*** *Further, flexible operation elevates EGS capacity factors close to their technical limit, optimising its dispatch around renewable availability (Supplementary Figure 3). Thereby, flexible EGS improves on already high capacity factors of inflexible EGS (95%-99%). We find these differences in electricity generating EGS dispatch mostly to occur in times of low marginal electricity price (Supplementary Figure 4). During those times, inflexible EGS ceases production while flexible EGS does not. It appears renewable cannibalisation squeezes inflexible EGS out of the market while flexible EGS can shift output to later markets, increasing market value and resulting in the observed increased capacity expansion. In the model, which renewable power source–wind, solar, hydro or EGS–dispatches during abundant renewable availability is determined by their marginal price which might not replicate real marginal prices. Therefore in the real system, dispatch outcomes during abundant renewable generation could be different. However during these times, as the marginal price vanishes, system value vanishes too, and dispatch does not affect these technologies' investment case. Conversely, flexible EGS's ability to shift generation out of a long market into a short*

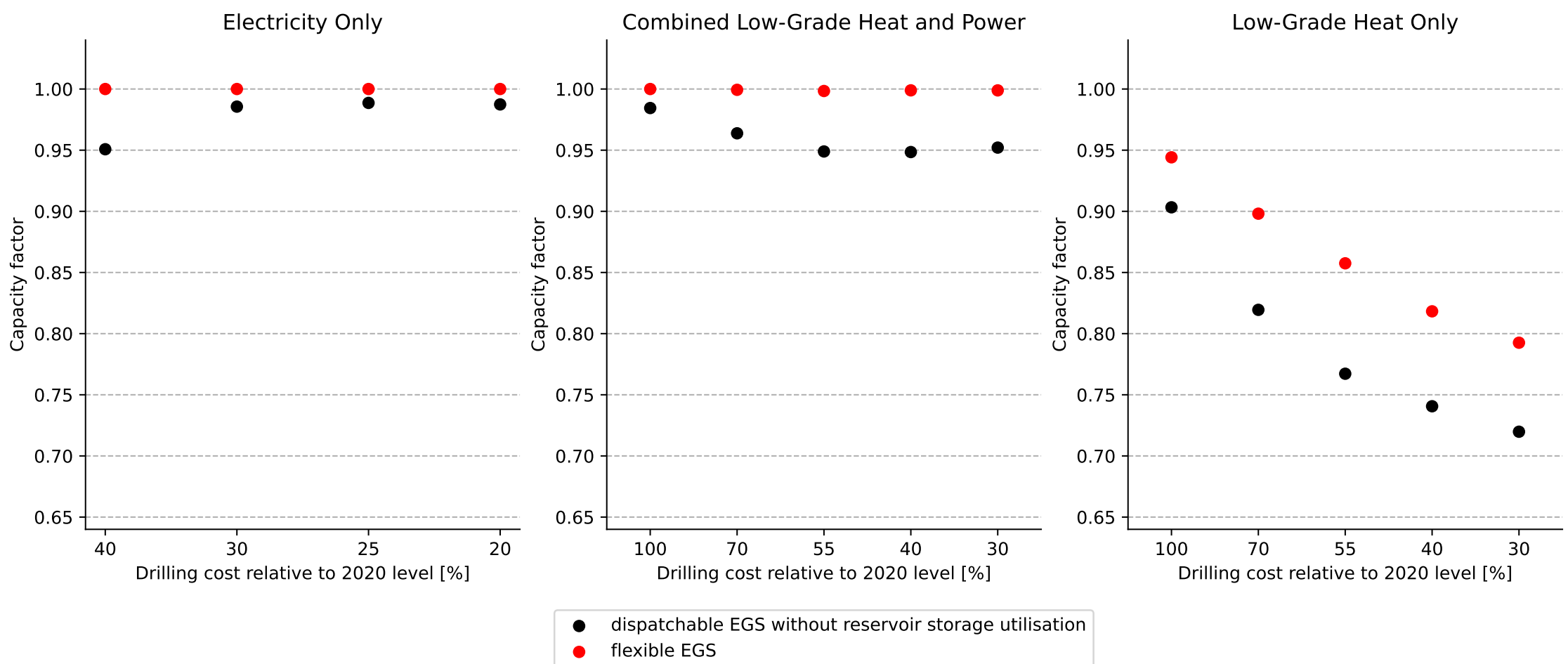

Figure 3: Weighted average capacity factors for different EGS modes and cost reductions. [*Added to Appendix*]

> *one is a real effect that enhances EGS market value. The lowest capacity factors are observed for heat-only EGS under extreme cost reductions, where EGS supply saturates heat demand and constrains supply to the profile of demand.*

RC 1.6 Cost-reduction assumptions need stronger justification and/or disaggregation. You assume large, uniform reductions to total EGS CAPEX (applied broadly, not component-wise). Please either: (1) disaggregate CAPEX reductions (drilling vs stimulation vs surface plant), or (2) provide a stronger evidence-based rationale for applying a blanket scaling to total CAPEX. As written, the results appear highly sensitive to an assumption that is not yet empirically supported at commercial scale.

AR: We agree this is a crucial point. Looking at the existing text, we believe there is some reasoning toward justifying blanket cost reduction assumptions but have added another sentence to bolster the argument. In particular, we believe the aggregate cost assumptions enable the reader to map the findings of the paper to the question they could have about the subject while playing into the strengths of the underlying methodology. Statements about the economic competition between technologies, i.e. EGS aggregate cost against its competitors, is where the model shines, especially in light of geographic and temporal dimensions. Meanwhile, given the uncertainty about which component will be subject to which learning rate, it seems useful to increase the reader's utility of the paper, regardless of the outcome that will actually manifest. We do acknowledge that modelling disaggregated cost reductions has upsides, but due to the uncertainty we believe the aggregated approach is a reasonable one. This is what we aim to express through the new sentence. We show the relevant passages below for convenience, and highlight the new sentence in bold.

> ***[(Mostly) previously added to Methods: "Modelling Enhanced Geothermal Systems"]*** *The technology-learning assumption in [3] is implemented as a* blanket *cost reduction of the* total *EGS capital cost, rather than as separate learning rates for individual sub-components,*

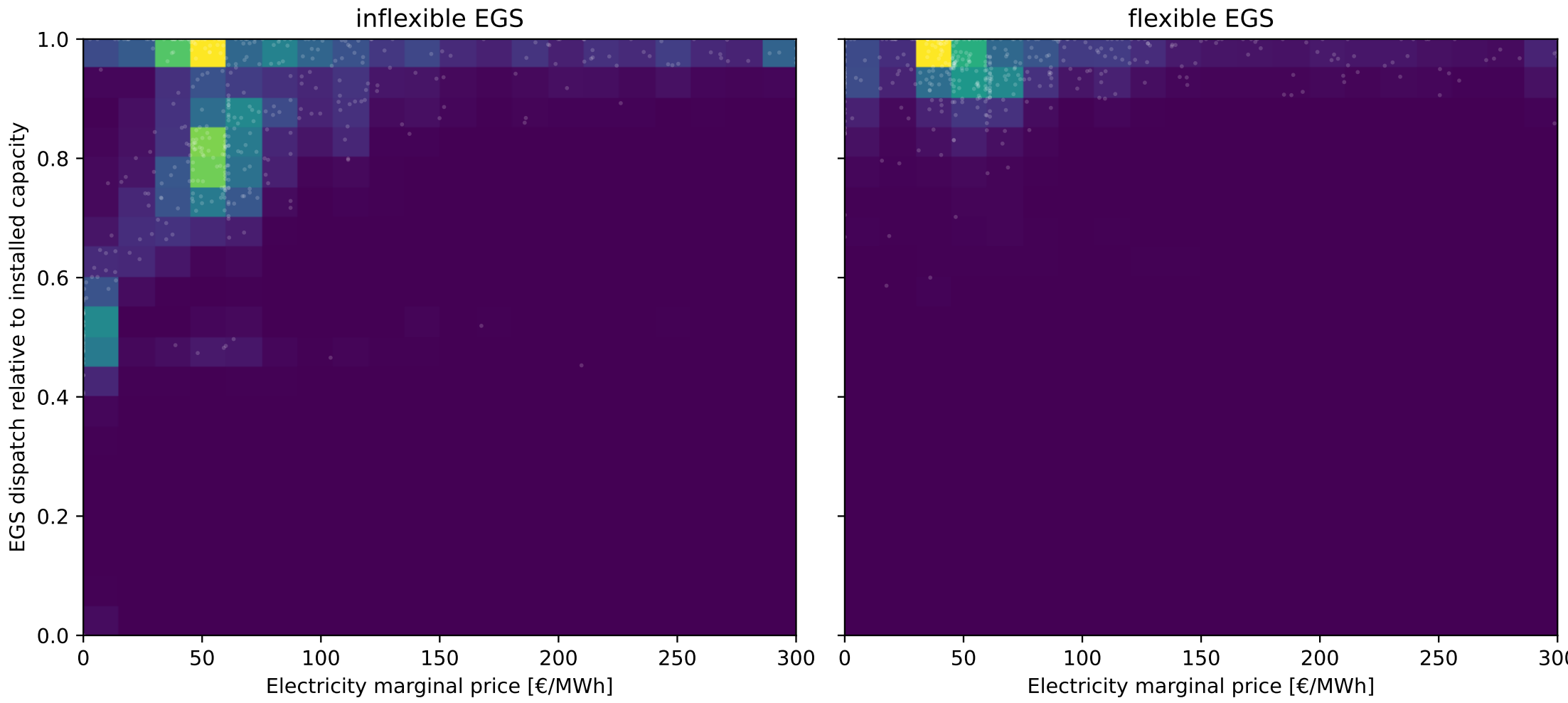

Figure 4: Capacity factors of electricity-generating EGS versus electricity marginal price. We compare inflexible and flexible EGS for 60% drilling cost reduction relative to 2020 cost. Each point counted in the contour represents one unique snapshot-bus pair in the model. [*Added to Appendix*]

> *and that assumption is inherited into the present paper. In particular, the learning factor is multiplied with the aggregated CAPEX (see Supplemental Material Eq. S1 in [3]), which sums well drilling & completion costs and surface power-plant costs (i.e. the ORC plant), alongside other project components.* ***This approach enables readers to map the cost reduction they are interested in, regardless of the component responsible for the reduction, to the findings here.*** *Neither [3] nor our implementation models seismicity-related costs endogenously; such effects can only be represented via an assumed net CAPEX adjustment. Analogously, the present paper translates the learning assumption into an aggregated representation: we scale the full EGS cost pipeline, i.e. drilling and the ORC surface plant, by a single cost-reduction factor relative to the 2020 baseline. The results therefore represent* net *project cost reductions irrespective of which specific technological advances (e.g. improvements in drilling equipment, reduced seismicity-related costs etc.) ultimately deliver them. Policy risk-sharing could change the effective private cost baseline; we note this in Limitations.*

> ***[Added in Revision 2 to Methods: "Limitations"]*** *Our cost-reduction scenarios are implemented as a multiplicative factor on aggregated EGS CAPEX (well-field/drilling and, where applicable, the ORC surface plant), following [3] where learning is applied to total CAPEX (Supplementary Eq. S1). Consequently, neither [3] nor our implementation endogenously allocates costs between individual mechanisms, such as higher drilling expenditures from deeper wells versus lower expenditures associated with induced-seismicity mitigation; these effects can only be reflected insofar as they change the* total *project CAPEX captured by the applied factor. Moreover, local regulation could change the effective project cost and thereby complicate comparison to this work.*

RC 1.7 Avoid overstating feasibility; keep conclusions aligned with "threshold/scenario" nature. The manuscript correctly notes that high drilling costs currently make EGS economically uncompetitive. However, some "market integration pathway" phrasing reads as if large-scale feasibility is imminent. Please tighten the framing to emphasize that your results identify conditions/thresholds under which EGS could become competitive, rather than suggesting competitiveness under current conditions.

AR: The EGS cost reduction scenarios evaluated here are hypothetical, and our paper is designed around that uncertainty. We agree in that context, the tone in some passages sounds off. We have adjusted the respective passages and hope the resulting text is more appropriate.

> ***[Added to Discussion: "Spatial Distribution of EGS Adoption"]*** *In summary, district heat and low-temperature industry demands, under a carbon-neutral constraint, could offer EGS a geographically widespread market opportunity, even at current or close-to-current drilling cost.*

> ***[Added to Discussion: closing paragraph]*** *In such a system, we find that EGS could already be cost-competitive for heat generation in regions with highly suitable geology and district heating networks or low-temperature industry heat demands.*

RC 1.8 If your implied EGS LCOE remains well above plausible market prices (today or in-model), that indicates economic non-competitiveness, not that EGS is "not technically feasible." Please ensure wording throughout distinguishes technical feasibility (can it be built/operated) from commercial feasibility (will it compete without support).

AR: We appreciate this distinction and have adjusted the wording throughout.

> ***[Added to Results: "Spatial Aspects of EGS Adoption"]*** *In contrast, heat (co-)generating EGS is economically viable across a broader range of regions, even for low cost reductions.*

> ***[Added to Results: "Spatial Aspects of EGS Adoption"]*** *Echoing the findings of the previous section, electricity-generating EGS is only cost-competitive in scenarios with significant cost reductions (Figure 5).*

> ***[Added to Results: "Spatial Aspects of EGS Adoption"]*** *Supplementary Figure A.13 shows a more system-centric perspective by showing the drilling CAPEX at which EGS becomes economically competitive.*

RC 1.9 Explicitly state the competitiveness threshold implied by your study. You state that electricity-only EGS competitiveness requires drilling costs to fall to $\sim$40% of 2020 levels. This

is an important takeaway and should be highlighted clearly in the abstract/discussion, along with the associated uncertainty and realism of achieving that reduction.

AR: We agree this is a key result and have made it more explicit. The threshold is now stated explicitly in both the Abstract and the closing paragraph of the Discussion.

> ***[Added to Abstract]*** *If drilling costs were to decrease by approximately 60% relative to 2020 costs, EGS would become competitive in electricity markets, greatly expanding its market opportunity.*

> ***[Added to Discussion: closing paragraph]*** *If drilling costs reduce to around 40% of 2020 levels, EGS that generates only electricity would become cost-competitive with other renewable resources, potentially enabling large-scale adoption in Europe of 20–100 GW_{el} power capacity.*

Reviewer 2

General Response to Reviewer 2:

We thank the reviewer for the response and are glad to hear the clarity has improved. The concern that is raised here is a valid one that we partially share.

RC 2.1 The quality of the paper was improved with the last version and my concerns in former reviews were addressed. The restrictions of the findings are given and the reader can now better understand for what the conclusions are given. However, the paper lost focus by the now given structure. It is hard to follow that the conclusions are made and the methods are described in a later chapter after the discussions. Nevertheless, I recommend minor revision of this manuscript because a more focused revision requires a totally new written article.

AR: Between the co-authors and myself, there are diverging views on the optimal ordering of sections, however ultimately our hand was forced by the formatting requirements of the journal. One change we have made since the last round of corrections is the removal of a dedicated Conclusions section, and have instead folded a tightened version into the Discussion. We do not believe this is sufficient to altogether address the concern expressed here, but it could still help towards improving clarity.

References

[1] J. Mann, J. Perkins, M. Lindovska, S. Harvey, Assessment of locational wholesale electricity market design options in GB, Tech. rep., FTI Consulting, commissioned by Ofgem, supported by Energy Systems Catapult (Oct. 2023).
URL https://www.ofgem.gov.uk/publications/assessment-locational-wholesale-pricing-great-britain

[2] Ember, European electricity prices and costs, data tool. Accessed: 2026-03-29 (2026).
URL https://ember-energy.org/data/european-electricity-prices-and-costs/

[3] A. Aghahosseini, C. Breyer, From hot rock to useful energy: A global estimate of enhanced geothermal systems potential, Applied Energy 279 (2020) 115769.

[4] European Energy Exchange AG, Market data hub, accessed: 2 April 2026 (2026).
URL https://www.eex.com/en/market-data/market-data-hub

[5] U.S. Energy Information Administration, Electricity, Coal, and Renewables Long-Term Modeling Team, Discussion on capital cost and performance characteristic estimates for new generating technologies, Technical report, U.S. Energy Information Administration, Washington, D.C., accessed 11 July 2025 (Sep. 2023).
URL https://www.eia.gov/

[6] M. J. Aljubran, R. N. Horne, Power supply characterization of baseload and flexible enhanced geothermal systems, Scientific Reports 14 (2024) 17619. doi:10.1038/s41598-024-17619-0.
URL https://www.nature.com/articles/s41598-024-17619-0

[7] T. Brown, J. Hörsch, D. Schlachtberger, Pypsa: Python for power system analysis, arXiv preprint arXiv:1707.09913 (2017).
